# Identification and characterization of miRNAome in root, stem, leaf and tuber developmental stages of potato (*Solanum tuberosum* L.) by high-throughput sequencing

**DOI:** 10.1186/1471-2229-14-6

**Published:** 2014-01-07

**Authors:** Nisha Lakhotia, Gopal Joshi, Ankur R Bhardwaj, Surekha Katiyar-Agarwal, Manu Agarwal, Arun Jagannath, Shailendra Goel, Amar Kumar

**Affiliations:** 1 Department of Botany, University of Delhi, Delhi 110007, India; 2 Department of Plant Molecular Biology, University of Delhi South Campus, New Delhi 110021, India

**Keywords:** Potato, Tuberization, microRNA, High-throughput sequencing, Digital expression profiling, qRT-PCR, Target, Gene ontology, RLM-RACE

## Abstract

**Background:**

MicroRNAs (miRNAs) are ubiquitous components of endogenous plant transcriptome. miRNAs are small, single-stranded and ~21 nt long RNAs which regulate gene expression at the post-transcriptional level and are known to play essential roles in various aspects of plant development and growth. Previously, a number of miRNAs have been identified in potato through *in silico* analysis and deep sequencing approach. However, identification of miRNAs through deep sequencing approach was limited to a few tissue types and developmental stages. This study reports the identification and characterization of potato miRNAs in three different vegetative tissues and four stages of tuber development by high throughput sequencing.

**Results:**

Small RNA libraries were constructed from leaf, stem, root and four early developmental stages of tuberization and subjected to deep sequencing, followed by bioinformatics analysis. A total of 89 conserved miRNAs (belonging to 33 families), 147 potato-specific miRNAs (with star sequence) and 112 candidate potato-specific miRNAs (without star sequence) were identified. The digital expression profiling based on TPM (Transcripts Per Million) and qRT-PCR analysis of conserved and potato-specific miRNAs revealed that some of the miRNAs showed tissue specific expression (leaf, stem and root) while a few demonstrated tuberization stage-specific expressions. Targets were predicted for identified conserved and potato-specific miRNAs, and predicted targets of four conserved miRNAs, miR160, miR164, miR172 and miR171, which are *ARF16* (*Auxin Response Factor 16*), *NAM* (*NO APICAL MERISTEM*), *RAP1* (*Relative to APETALA2 1*) and *HAM* (*HAIRY MERISTEM*) respectively, were experimentally validated using 5′ RLM-RACE (RNA ligase mediated rapid amplification of cDNA ends). Gene ontology (GO) analysis for potato-specific miRNAs was also performed to predict their potential biological functions.

**Conclusions:**

We report a comprehensive study of potato miRNAs at genome-wide level by high-throughput sequencing and demonstrate that these miRNAs have tissue and/or developmental stage-specific expression profile. Also, predicted targets of conserved miRNAs were experimentally confirmed for the first time in potato. Our findings indicate the existence of extensive and complex small RNA population in this crop and suggest their important role in pathways involved in diverse biological processes, including tuber development.

## Background

In recent years, small non coding RNAs (small ncRNAs) have emerged as key regulators of gene expression in both plants and animals [1,2]. In plants, there are many classes of small ncRNAs and the two major classes that have been well-studied and defined are miRNAs and siRNAs [1,2]. Although both are functionally similar, they have different mode of biogenesis. siRNAs, 20–24 nt long, are processed from perfect double stranded RNA. They are mainly involved in heterochromatin modification, maintaining genome integrity by silencing of transgenes and transposons, defence against viruses and regulation of gene expression [2]. There are diverse siRNA species depending on the Dicer-like (DCL) proteins involved in the production, such as natural antisense transcript-derived siRNAs (24 nt), heterochromatic siRNAs (24 nt) and trans-acting siRNAs (21 nt) [1]. miRNAs are small, endogenous, single-stranded and ~21 nt long RNAs that regulate gene expression at the post-transcriptional level by degrading or repressing translation of targeted mRNAs, depending on the extent of complementarity between miRNA and mRNA [1,2].

miRNAs are transcribed by RNA polymerase II into an imperfect long folded back structure, called as primary miRNA (pri-miRNA). The pri-miRNA is cleaved by Dicer-like 1 (DCL1) protein (endoribonuclease having RNase III activity), in association with other protein factors (HYL1 and others), into a precursor sequence with hairpin structure (pre-miRNA), which is further processed by DCL1 into a miRNA:miRNA* duplex [3]. One of the strands of this duplex (guide strand or mature miRNA) gets incorporated into the silencing complex or effector complex (RISC- RNA Induced Silencing Complex) containing argonaute proteins, while the other strand (miRNA* or passenger strand) gets degraded. Within the complex, miRNA guides the target mRNA cleavage or inhibits translation with the help of one of the argonaute proteins (AGO1) [3]. There is increasing evidence which indicates that miRNAs play vital roles in stress responses and various developmental processes, including shoot apical meristem formation, leaf morphogenesis and polarity, floral organ identity, root development, vegetative phase change and vascular development [2,4].

A large number of miRNAs are evolutionary conserved among diverse species within the plant kingdom while several miRNAs, that are considered to be recently evolved show species-specificity and often express at lower levels relative to conserved miRNAs [5,6]. Due to their low expression levels, most of the species-specific miRNAs remained unidentified in many plant species. However, in recent years, with the advent of high-throughput sequencing technology, both species-specific and conserved miRNAs have been identified in diverse plant species [6-15].

Potato (*Solanum tuberosum* L.) is the fourth most important crop worldwide, after rice, wheat and barley. The main reasons for the increasing popularity of potato are high nutritional value combined with the simplicity of its propagation by vegetative reproduction. Potato tubers are derived from the swollen tips of specialized basal lateral juvenile shoots, called stolons. Tuber development is a highly systematic and well-coordinated morpho-physiological process that involves interactions between several environmental, biochemical and genetic factors [16-18]. Since control of tuber initiation impacts many commercial aspects of crop improvement, including scheduling, size grade distribution, yield and expansion of climate zone suitable for propagation, this is a high priority area for potato research. Several transcription factors, hormones, environmental and molecular signals have been studied that regulate tuberization [16-18]. Although, the potato genome sequencing project [19] has revealed that both gene family expansion and recruitment of existing genes for new pathways have contributed to the evolution of tuber development in potato, this phenomena still remains a challenge for plant biologists.

To date, only miR172 and miR156 have been shown to play a role in tuberization process [20,21] and only a few miRNAs have been shown to regulate defense genes in potato [22]. Since the potato genome has been completely sequenced recently [19], it will greatly help in identifying the entire population of small RNAs and their target genes that might be involved in various developmental processes in potato. In previous studies, through an *in silico* approach, many conserved miRNAs were identified in potato based on miRBase repository by using potato EST, GSS and nr (non-redundant) databases (existing in the National Centre for Biotechnology Information) [23-26]. Earlier, large scale sequencing studies were limited to the identification of only conserved miRNAs in potato [27]. A recent study reported 28 conserved and 120 potato-specific miRNA families in potato by deep sequencing of sRNA libraries from leaf and stolon tissues [28]. However, knowledge about the potato-specific miRNA population in different vegetative tissues and developmental stages of tuberization remain largely unknown.

In this study, we report a population of potato miRNAs from vegetative organs (leaf, stem and root) and four early development stages of tuberization that might be involved in regulating developmental processes, including tuberization. By taking advantage of the recently sequenced potato genome [19], 33 conserved miRNA families and 147 potato-specific miRNA were identified using high-throughput sequencing and bioinformatics pipeline. We also report the expression profiles of both conserved and potato-specific miRNAs in different tissues (leaf, stem and root) and different developmental stages of tuberization based on digital and qRT-PCR expression analyses. Additionally, the putative target genes for the conserved and potato-specific miRNAs were predicted and 5′ RLM-RACE was carried out to map the cleavage site of four conserved miRNAs targets. GO analysis of potato-specific miRNAs was also performed to gain a better understanding of their functions in various developmental processes in potato.

## Results

### Analysis of small RNA population

**Figure 1:**
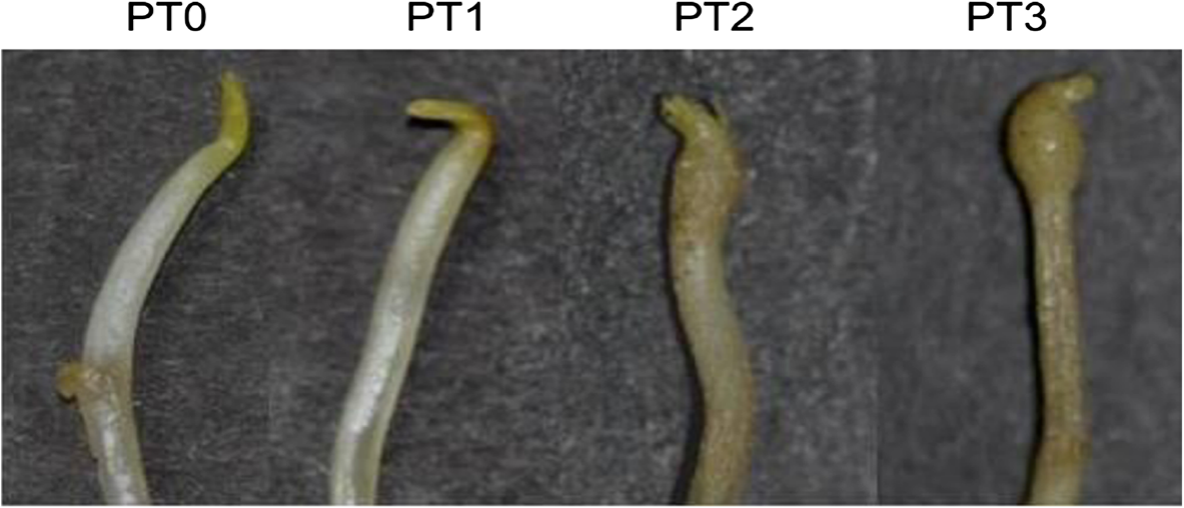
**Early developmental stages of potato tuber.** Samples were collected from field-grown potato plants during the period of tuberization. PT0 - unhooked stolon tip (0 stage), PT1 - hooked stolon tip (1^st^ stage), PT2 - sub-apical stolon swelling (2^nd^ stage), PT3 - tuber initiation (3^rd^ stage). See methods for details of physiological and environmental conditions in which potato plants were grown.

**Figure 2:**
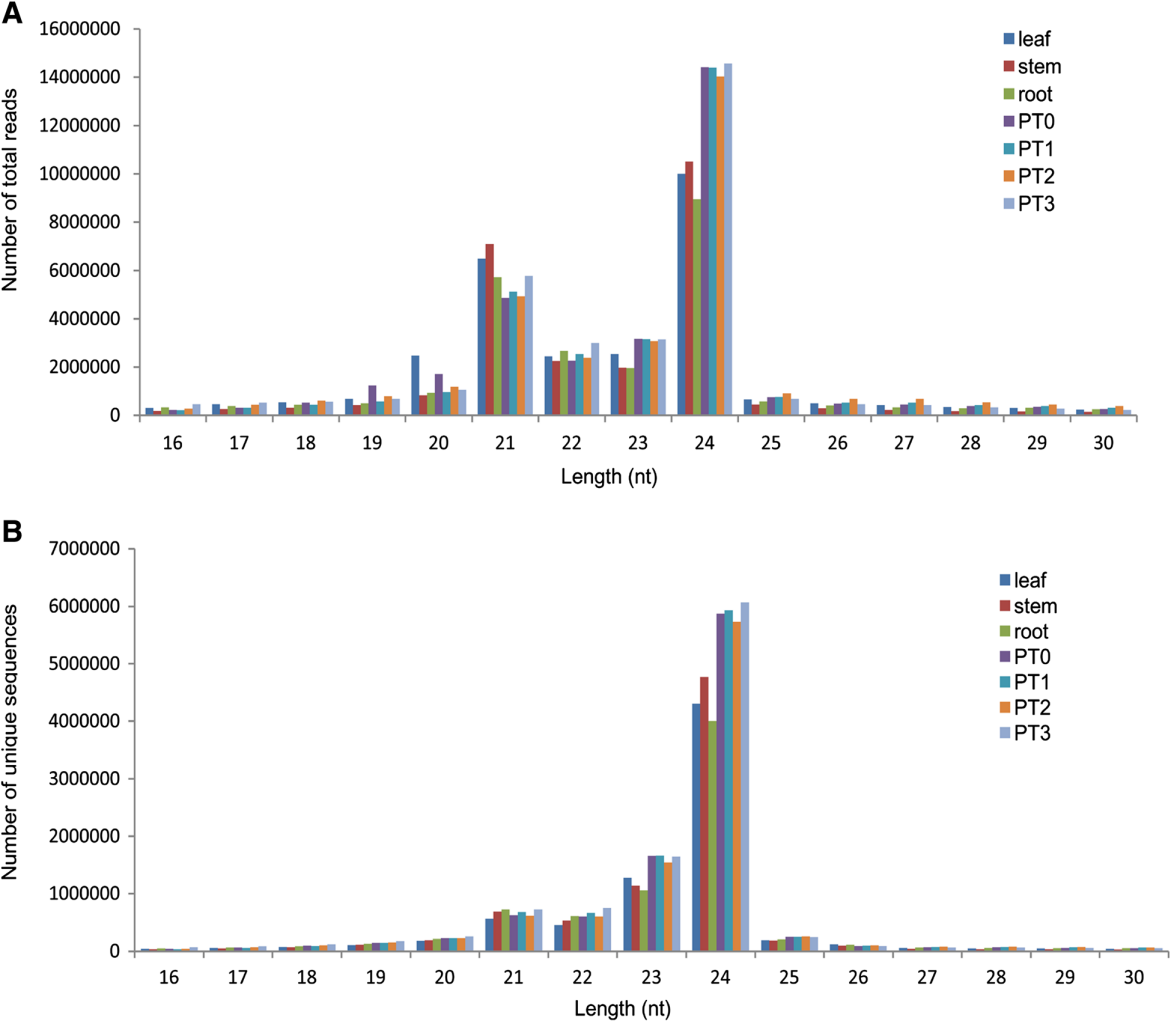
**Length distribution of small RNA sequences identified in all the small RNA libraries. A)** Size distribution of total reads. 24 and 21 nt length are most abundant reads among the small RNAs. **B)** Size distribution of unique small RNA sequences where 24 and 23 nt are the most abundant. nt - nucleotide, PT0 - 0 stage of tuberization, PT1 - 1st stage of tuberization, PT2 - 2^nd^ stage of tuberization, PT3 - 3^rd^ stage of tuberization.

To identify miRNAs in potato (*Solanum tuberosum* cv. Kufri Chandramukhi), seven small RNA libraries from leaf, stem, root and four early development stages of tuberization (Figure 1) were constructed and sequenced independently. Small RNA sequencing results yielded a total of 216 million raw reads from these seven libraries. The raw sequences were processed and filtered through several criteria to identify conserved and potato-specific miRNAs. After removing adaptor sequences, sequences smaller than 16 nt and larger than 30 nt, poor quality sequences and invalid sequences, a total of approximately 202 million raw reads, corresponding to approximately 39 million unique sequences, were obtained (Table 1). High quality trimmed sequences were further subjected to removal of non coding RNAs such as transfer or ribosomal rRNAs (t/rRNAs). The remaining sequences represented the small RNA population, which accounted for 83% of reads (Table 1), suggesting a useful set of sRNAs with reasonable sequencing depth. The size distribution analysis of these small RNA sequences showed that they exhibit similar pattern of distribution in length in all the libraries (Figure 2). The length of the sRNAs in each library varied from 16 nt to 30 nt and the majority of reads were 21 to 24 nt in length, as expected for DCLs cleaved products [1] (Figure 2). In all the small RNA libraries the 24 nt long sequence size class was the most abundant, followed by 21 nt (Figure 2A). This result was consistent with previous studies in other species such as *Arabidopsis thaliana*, tomato, pepper, cucumber, maize, peanut, pepper, *Citrus trifoliata* and rice [6-15] but in contrast to grapevine, wheat and Chinese yew where 21 nt or 23 nt class predominated the sRNA transcriptome [29-31]. Analysis of the size distribution of unique sequences revealed that 24 nt length read was the most abundant, followed by 23 nt class (Figure 2B). Thus, for the 21 nt size class, which is characteristic of an authentic miRNA, the number of total reads was found to be more compared to total number of unique sequences. The 23 nt size class might represent the degraded products of 24 nt small RNAs. The high abundance of 24 nt long small RNAs might indicate the complexity of the potato genome as they are mainly siRNAs that are associated with repeats and heterochromatic modifications [1]. These results suggest the existence of complex and diverse sRNA population in potato.

**Table 1:** Summary of the reads obtained from all the potato small RNA libraries

**Types of reads**	**Redundant**	**Non-redundant**
Raw reads	216540436^a^	45558960^e^
Filter by sequence length	12792087^b^	4757221^f^
Filter low-complexity sequences	12108^c^	6868^g^
Filter invalid sequences	1445447^d^	869520^h^
Filter by t/rRNA (matches out)	20965836	143079
Putative Small RNA Population	181324958	39782272

Note: a - (b + c + d) = 202290794, e - (f + g + h) = 3992535.

### Identification of conserved and potato-specific miRNAs in potato

**Figure 3:**
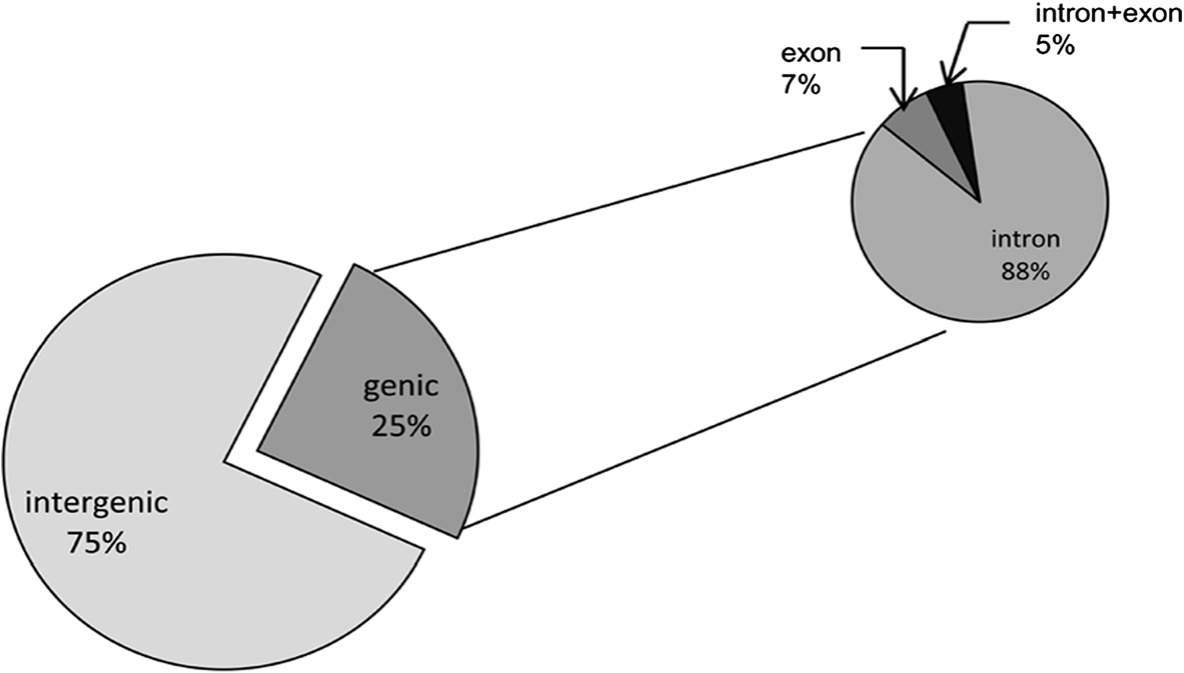
**Genomic locus distribution of predicted miRNA precursors.** Most of the miRNA precursors originate from intergenic regions, and in genic regions majority of them are encoded by introns.

In order to identify miRNAs, all unique reads obtained after filtering were submitted to the UEA sRNA toolkit-Plant version miRCat pipeline (http://srna-tools.cmp.uea.ac.uk/) [32] and were mapped onto the potato reference genome (PGSC DM assembly version 3) (http://potatogenomics.plantbiology.msu.edu/) with no mismatch. The 100 nt flanking regions of sRNA loci were excised from the genome. The secondary structures of these extracted regions were predicted using RNAfold (http://rna.tbi.univie.ac.at/cgi-bin/RNAfold.cgi) [33] to identify the potential precursor sequences. The resulting secondary structures were then trimmed and analysed by miRCat [32]. To recover the putative miRNA candidate, the potential precursors were screened based on the plant miRNAs prediction criterion as described by Meyers et al. [34]. To identify the conserved miRNAs in potato, all the predicted miRNA sequences were mapped against the known plant miRNAs, deposited in miRBase v20 [35], allowing up to 2 mismatches during the alignment. Only those sequences that matched to known plant miRNAs with 0–2 mismatches were considered as conserved miRNAs. The remaining sequences that had 3 or more than 3 mismatches or showed no homology to any previously known plant miRNAs were regarded as novel miRNAs or potato-specific miRNAs. The predicted pre-miRNAs loci were explored further to find out their distribution among the intergenic and genic regions of the genome. It was found that 262 pre-miRNAs were encoded by intergenic regions and only 86 were encoded by genic regions (Figure 3). In the genic regions, 76 of the pre-miRNAs were located in introns, 6 in exon regions and 4 in both intron and exon regions (Figure 3). These results suggests that majority of the pre-miRNAs in potato are transcribed from non-coding part of the genome.

#### Digital expression profiling of predicted miRNAs

The read abundance of predicted miRNAs in different libraries was normalized and expressed as transcripts per million (TPM) to measure the expression levels of these miRNAs in different samples (Additional file 1; Additional file 2; Additional file 3).

#### Conserved miRNAs

**Figure 4:**
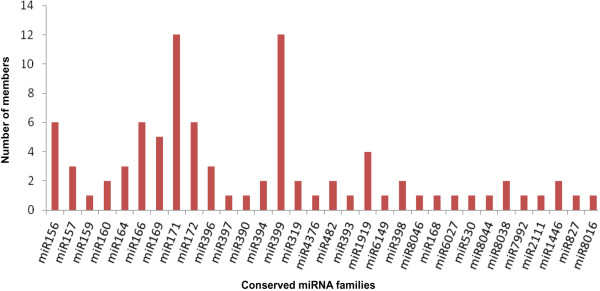
**Conserved miRNA families and their members identified in potato.** Graphical representation of the different members of conserved miRNA families identified by deep sequencing of small RNA libraries prepared from tissues of leaf, root, stem and four developmental stages of potato tuber.

A total of 89 miRNAs, belonging to 33 families, were identified as conserved miRNAs in potato since these were also detected in other plant species. Star sequences were detected for 75 conserved miRNAs (Additional file 1). The star sequences for the remaining conserved miRNAs were not detected which could be either due to their low expression count in the sequencing dataset or poor stability. A significant difference in the number of members was detected for each conserved miRNA family (Figure 4). The largest families identified were miR399 and miR171 with 12 members each. The second largest families, with 6 members each, were miR156, miR166 and miR172. Of the remaining families, 12 comprised of 2–5 members while 16 were represented by a single member (Figure 4). A considerable variation in expression levels was found among miRNA families. Of the 33 conserved miRNA families, miR156, miR157, miR166 and miR168 showed high abundance, similar to those observed in other species [11,12], each with total TPM >1,00,000 (Additional file 1). Among these, miR166 was the most abundantly expressed miRNA family (total TPM > 6,00,000). While miR8016, miR530, miR1446, miR8038 and miR7992 were found to be least abundant, each with total TPM less than 10 and amongst these poorly expressed miRNAs, miR7992 had lowest read number (Additional file 1). It was also observed that different members of a given family showed variations in their expression levels which suggest functional divergence within the family (Additional file 1). For example, the sequencing frequency of the miR172 family varied from 0.1–5778 TPM. Similar variations in read abundance were also observed among members of the miRNA families such as miR171 (0.1–1269 TPM), miR164 (0.2–6089 TPM), miR399 (0.5–232 TPM) and miR166 (23888–82963 TPM). Most of the miRNAs identified in potato were also found across various plant species, which suggests their highly conserved function.

#### Potato-specific miRNAs

**Figure 5:**
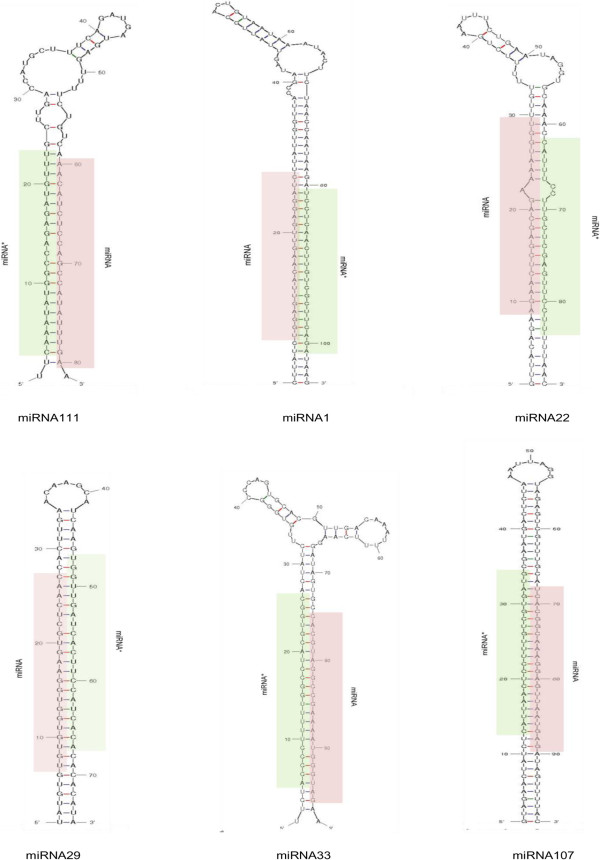
**Predicted secondary structure of precursors (Mfold) of few potato-specific miRNAs.** The mature miRNA sequences are highlighted in light red colour while the star sequences are highlighted in light green colour. Hairpin structure of all pre-miRNAs of potato-specific miRNAs, predicted using RNAfold, is given in Additional file 4.

A total of 3631 novel miRNA candidates were identified in potato that met our criteria and had no matches to any previously known plant miRNAs. These were therefore termed as potato-specific miRNAs and further categorised into (I) high-confidence potato-specific miRNAs - that had their star sequences detected in our sequencing dataset (Additional file 2) and (II) candidate potato-specific miRNAs - for which star sequences were not detected (Additional file 3). Of these miRNAs, 147 belonged to category I while the remaining 3484 sequences belonged to category II. Out of the 3484 category II sequences, 3372 candidate miRNAs were sequenced with less than 10 reads and thus excluded from the analysis resulting in 112 candidate potato-specific miRNAs. As the detection of star sequence is considered to increase the authenticity of predicted miRNAs [6], potato-specific miRNAs with star sequences (category I) were selected for further analysis and termed as potato-specific miRNAs in this study. The stem loop structure of precursors of all potato-specific miRNAs were predicted using RNAfold [33], and are provided in Additional file 4. Figure 5 shows the secondary structure of precursors of a few potato-specific miRNAs, predicted using Mfold web server [36]. The predicted hairpin length of these precursors were found to be in the range of 64–220 nt, similar to that observed in other plant species [12,37] (Additional file 2). The adjusted minimum free energy (MFE) of miRNA precursors ranged from -82.3 to -25.9 kcal mol^-1^, with an average of about -54 kcal mol^-1^, which was in accordance with the free energy values reported for miRNA precursors in other plant species (-59.5 kcal mol^-1^ in *A. thaliana*, -71.0 kcal mol^-1^ in *O. sativa* and -50.01 kcal mol^-1^ in peanut) [12] (Additional file 2). This indicates a high stability of the hairpin structures. Thus, the pre-miRNAs had MFE much lower than the free folding energies of transfer RNA (-27.5 kcal/mol) and ribosomal RNA (-33 kcal/mol) [38].

The sequencing frequencies of a majority of potato-specific miRNAs were found to be low, consistent with the hypothesis that species-specific miRNAs are often expressed at a lower level as compared to conserved miRNAs [5,6]. Most of these miRNAs (about 65%) were detected with total TPM less than 5 (Additional file 2). However, a few miRNAs (14) were found to have total TPM more than 100. Among all the potato-specific miRNAs, miRNA53 and miRNA72 were the most abundant, with total TPM more than 15000 (Additional file 2).

Some predicted conserved and potato-specific miRNAs displayed significant variation in expression level among the different tissues (Additional file 1 and Additional file 2). For example, conserved miRNA - miR156 exhibited high expression during tuberization stages, with highest level at PT0 (Additional file 1). Among potato-specific miRNAs, miRNA72 had highest sequencing frequencies in stem and displayed significant change in expression level during tuberization stages (with highest expression at PT1) (Additional file 2). These findings suggest the probable role of these regulatory miRNAs in specific tissues or during specific developmental stages in potato. In this study, biological replicates were not used for sequencing. Hence, the digital expression data only gives an indication of variation in expression level between different tissues. This could be further confirmed by qRT-PCR as has been done for some of the miRNAs in this study (see below).

### Expression profile of conserved and potato-specific miRNAs in different tissues by qRT-PCR

**Figure 6:**
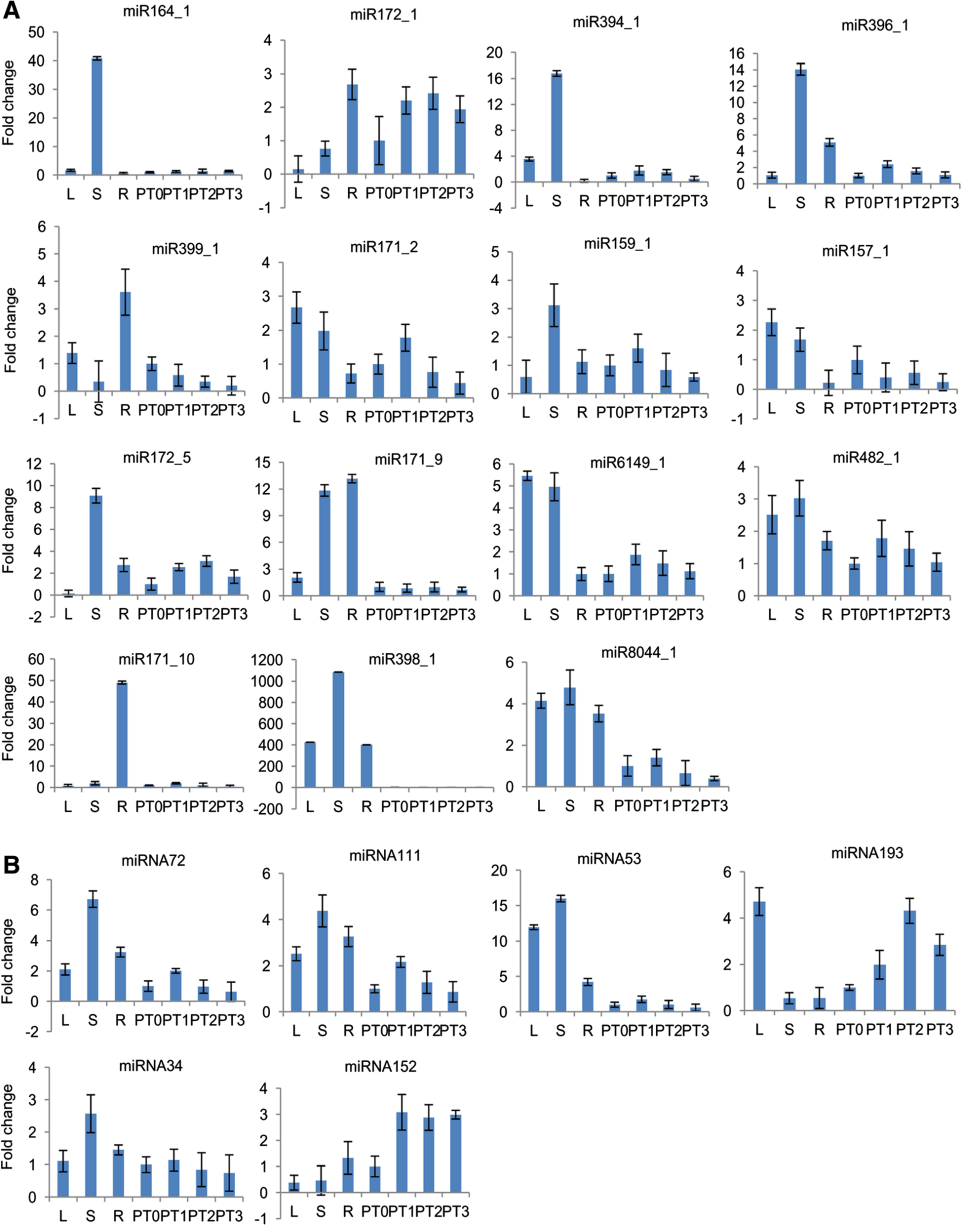
**Expression analysis of miRNAs in different tissues and different development stages of tuberization in potato by qRT-PCR. A)** The expression profile of 15 conserved miRNA identified in this study in potato (miR164_1, miR172_1, miR394_1, miR396_1, miR399_1, miR171_2, miR159_1, miR157_1, miR172_5, miR171_9, miR6149_1, miR482_1, miR171_10, miR398_1, miR8044_1). **B)** The expression profile of 6 potato-specific miRNAs identified in this study (miRNA72, miRNA111, miRNA53, miRNA193, miRNA34, miRNA152). The expression level of each miRNA in PT0 was set as control and taken as 1, and expression level in all other tissues was quantified relative to it. 18S rRNA was taken as an endogenous control. L - Leaf, S - Stem, R - Root, PT0 - 0 stage of tuberization, PT1 - 1^st^ stage of tuberization, PT2 - 2^nd^ stage of tuberization, PT3 - 3rd stage of tuberization (for details of the tuberization stages see Figure 1).

Quantitative real time PCR was performed to determine the expression levels of conserved and potato-specific miRNAs in different vegetative tissues (leaf, stem and root) and four early developmental stages of tuberization (PT0–PT3; Figure 1) to validate results obtained through digital expression profiling of potato miRNAs (described above). In this study, based on the TPM values, we selected 15 conserved miRNAs and 6 potato-specific miRNAs (with TPM > 10). The expression level of each miRNA in PT0 was set as control, taken as 1 and expression level in all other tissues was quantified relative to it. The expression profiles of conserved and potato-specific miRNAs have been shown in Figure 6A and Figure 6B, respectively.

#### Expression profile of miRNAs in vegetative tissues

Among conserved miRNAs, the expression of miR164_1 and miRNA171_10 appeared to be restricted to stem and root, respectively while miR8044_1 showed abundant expression in all the examined vegetative tissues. miR394_1 exhibited the highest expression in stem and moderate expression in leaf. miR171_2 and miR157_1 showed low expression in root whereas relatively high expression was observed in leaf and stem. The expression of miR396_1 and miR172_5 was found to be most abundant in stem and moderate in root whereas the expression of miR399_1 was relatively high in root. miR172_1 had high expression level in root, low expression in stem and could rarely be detected in leaf while miR159_1 demonstrated highest expression in stem but low expression in leaf and root. The expression of miR6149_1 and miR482_1 was higher in leaf, stem and lower in root. miR171_9 showed strong expression in stem and root but weak expression in leaf and miR398_1 was found to be expressed predominantly in stem, followed by leaf and root.

Among potato-specific miRNAs, miRNA53 had higher level of expression in leaf and stem, and moderate level in root. miRNA193 appeared to be highly expressed in leaf and weakly in stem and root. The expression pattern of miRNA111, miRNA72, miRNA34 were found to be very similar - high expression in stem, followed by root and leaf while miRNA152 exhibited moderate level of expression in root and low expression in leaf, stem.

#### Expression profile of miRNAs during tuber developmental stages

The expression of miR164_1, miR399_1, miR157_1, miR398_1, miR171_10, miR171_9 (conserved miRNAs) and miRNA53 (potato-specific miRNAs) could barely be detected during tuberization stages. Among potato-specific miRNAs, miRNA111, miRNA72 and among conserved miRNAs, miR171_2, miR396_1, miR159_1, miR6149_1, miR8044_1 and miR482_1 had very similar pattern of expression during different tuberization stages. They showed a slight increase in expression at PT1 stage and then a gradual decrease during later stages whereas miRNA34 and miR394_1 was found to be expressed at low level in all the stages with no variation in expression levels. miR172_5 showed moderate level of expression during tuberization - similar expression level at PT1 and PT2 stage, then a slight decrease in abundance at PT3 stage. The expression of miRNA193, miRNA152 (potato-specific miRNAs) and miR172_1 was relatively high during tuberization. miRNA193 and miR172_1 showed a gradual increase in abundance from PT1–PT2, reaching the maximum expression at PT2, then a decrease thereafter whereas miRNA152 showed similar level of expression during all the stages.

It was found that digital expression data and qRT-PCR expression profiles of miRNAs in different tissues and developmental stages of tuberization do not correlate with each other. The probable reason for this variation could be the biasness of RNA ligase activity during small RNA library preparation [39].

### Target prediction for conserved and potato-specific miRNAs

In order to explore the functional role of all the identified miRNAs in diverse biological processes, their putative target genes were predicted using the open source web server psRNATarget [40] with default parameters (see Methods). The transcript sequences of the potato genome (Group Phureja DM1-3 516R44) were used as a reference set. Additional file 5 and Additional file 6 provide detailed information regarding the predicted targets for conserved and potato-specific miRNAs respectively. A majority of the miRNAs were found to target more than one transcript.

Among conserved miRNA targets, most of them were found to be transcripts coding for transcription factors (Additional file 5), such as Squamosa promoter-binding protein (regulated by miR156), GRAS family transcription factors (targeted by miR171), GAMYB-like2 (targeted by miR159), APETALA2 (target of miR172), NAC domain containing protein (targeted by miR164), Auxin response factors (regulated by miR160), PHAVOLUTA-like HD-ZIPIII protein (target of miR166) and nuclear transcription factors - YA4, YA5, YA6 (targeted by miR169). In addition to transcription factors, other targets included mRNA coding for F-box family protein (miR394), disease resistance protein and fiber expressed protein (miR159), laccase (miR397), salt tolerance protein (miR157), UDP-glucoronate decarboxylase 2 (miR164), DNA binding protein (miR166 and miR396), NL25 disease resistance protein (miR482), protein phosphatase and kinase (miR390), AGO1-1 (miR168), galactose oxidase (miR6149) and proteins with unknown functions (Additional file 5). Most of the conserved miRNA targeted plant transcription factors (SBP, NAC, ARF, GRAS, YA, AP2) were found to be similar to the conserved miRNA targets predicted in *Arabidopsis* and other plants [2,9,41], underlining the role of conserved miRNAs in essential biological processes.

For potato-specific miRNAs, targets were predicted successfully for 142 out of 147 miRNAs. Target genes were not found for the remaining five miRNAs which could be due to stringent cut-off used in our prediction analysis to reduce the number of false positives and to obtain reliable results. Their targets included mRNA coding for tropinone reductase I, Cdk10/11, endo-1,4-beta-D-glucanase, XH/XS domain containing protein, ankyrin repeat containing protein, cytochrome P450, alpha amylase, glucosyltransferase and others (Additional file 6). A few potato-specific miRNAs showed lipoxygenase and protein phosphatase type 2A, that are known to be involved in potato tuberization [42,43], as their predictive targets (Additional file 6). In addition, some potato-specific miRNAs were also found to target transcription factors including NAC domain protein, GRAS family transcription factors, MADS-box family protein and ARF domain class transcription factor (Additional file 6). These transcription factors have been known to be involved in regulating developmental processes in plants [2]. These results imply that these potato-specific miRNAs might be involved in some specific developmental as well as essential biological processes in potato. Many of the potato-specific miRNA target transcripts that code for proteins of unknown function.

**Figure 7:**
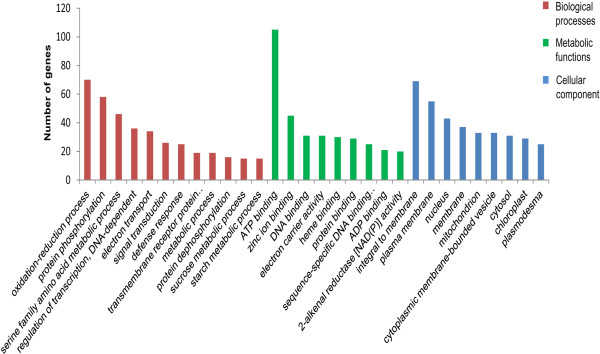
**GO analysis and categorization of putative targets of potato**-**specific miRNAs.** According to GO annotation, the putative targets of potato-specific miRNAs are categorized into biological process, molecular function and cellular component.

Further, all the predicted targets of potato-specific miRNAs, identified in this study, were subjected to GO analysis using Blast2GO [44] to gain a better understanding of their functions. GO terms were categorized into biological processes, molecular functions and cellular component. The GO results revealed that within the cellular component category, most of the genes are localized in membrane and nucleus (Figure 7). In the category of molecular functions, majority of targets are involved in binding activities (Figure 7). With respect to biological processes, most of the genes participate in oxidation-reduction processes, metabolic processes, regulation of transcription, transport, signal transduction and defense responses (Figure 7). Detailed information of GO annotation is provided in Additional file 7.

### Experimental validation of conserved miRNA targets

**Figure 8:**
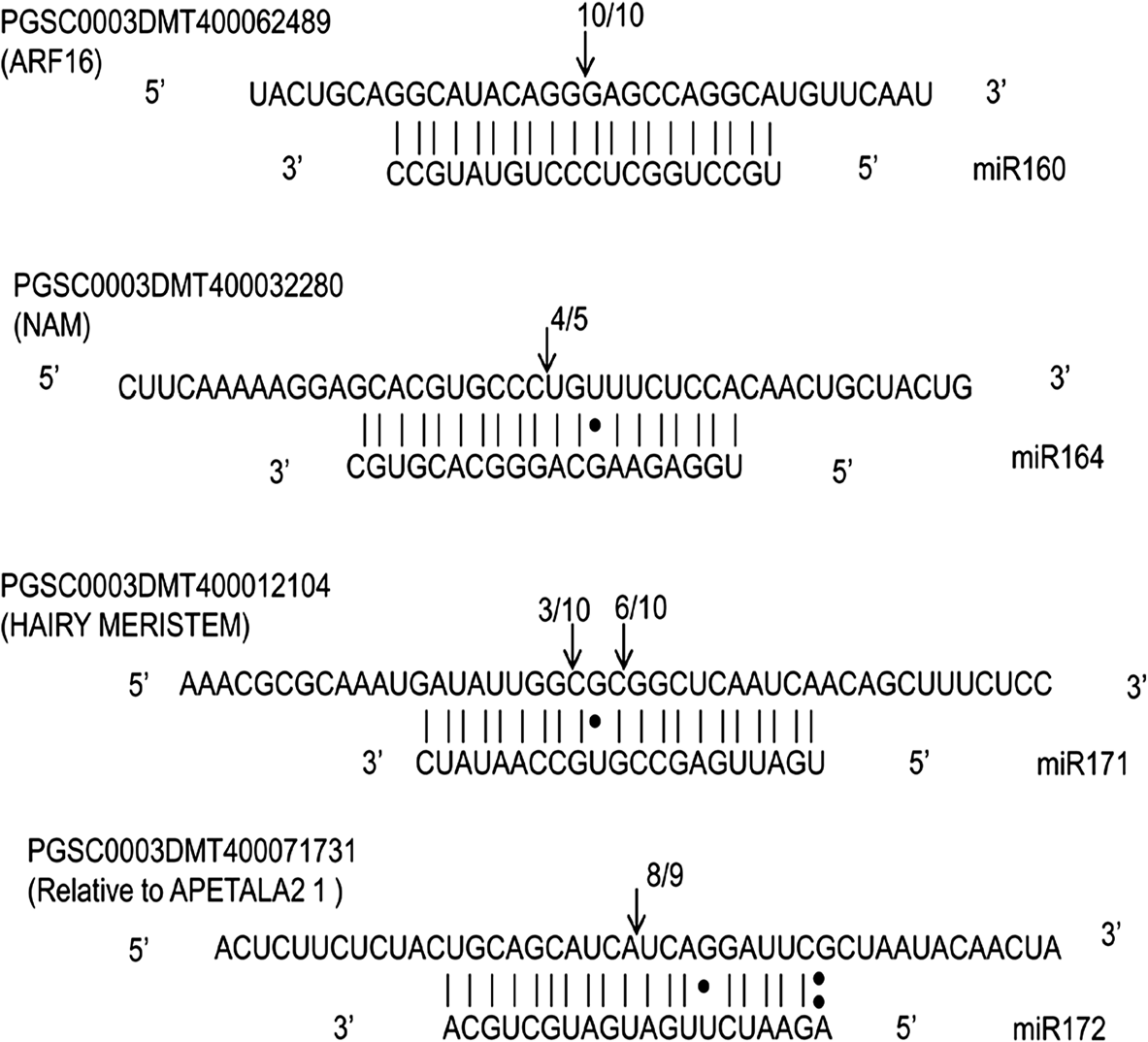
**Target validation of conserved miRNAs by 5′ RLM-RACE.** Cleavage site of four conserved miRNAs targets were experimentally verified. The arrow indicates the cleavage site with the frequency of cloned RACE products shown above it. The vertical lines indicates matched base pairs and dot denotes G:U pairs, ‘:’ symbolize mismatches.

Cleavage site of predicted targets of four conserved miRNAs in potato were further verified by 5′ RLM-RACE. The predicted targets of miR160, miR164, miR172 and miR171 were *ARF16*, *NAM*, *RAP1* and *HAM*, respectively. The cleavage site was found to be between 10–11 bases, from the 5′ end of the miRNA, for *ARF16* (PGSC0003DMT400062489), *NAM* (PGSC0003DMT400032280) and *RAP1* (PGSC0003DMT400071731) (Figure 8). However, *HAM* ((PGSC0003DMT400012104) was found to be cleaved between 10–11 and 13–14 bases from the 5′ end of the miRNA binding site (Figure 8).

## Discussion

Since the discovery of sRNAs (miRNAs & siRNAs) as regulators of gene expression in *C. elegans*[45], there has been intense interest in the sRNA biology in eukaryotic organisms. In plants, extensive studies in the past several years have clearly shown that miRNAs regulate gene expression at the post-transcriptional level by degrading or repressing translation of targeted mRNAs and play important roles during plant development and growth [2]. Here, we report identification and characterization of a population of both conserved and potato-specific miRNAs from different vegetative tissues and various well-defined stages of tuberization. Also, we discuss the potential role of these validated miRNAs in various developmental processes in potato.

In this study, with the application of high-throughput sequencing technology, we identified 89 conserved miRNA (belonging to 33 families), 147 potato-specific miRNAs and 112 candidate potato-specific miRNAs, based on the sequenced potato genome dataset [19]. Consistent with previous reports [5,6], a majority of the potato-specific miRNAs predicted in our analysis, showed low sequencing frequencies as compared to conserved miRNAs in the dataset. The majority of the pre-miRNAs were found to be encoded by intergenic regions which is also consistent with earlier findings [46].

The analysis of sRNA population in potato revealed that 24 nt long sRNAs (which mainly contains heterochromatic siRNAs) dominated the sRNA transcriptome, in both total reads and unique sequences, as observed in many other plant species [6-15]. The high percentage of 24 nt sRNAs suggests an important role of this class in maintaining genome integrity by heterochromatic-histone modification [1]. The high ratio of total reads/unique sequences for 21 nt length class indicated the high abundance of this size class. The majority of the identified miRNAs were 21 nt in length, which is the canonical size for miRNAs generated from DCL1 processing. Only a few miRNAs showed minor variations in size (20 or 22 nt long) which could be due to inaccuracy of DCL1 processing [7].

Previously, many conserved miRNAs were reported in potato by a computational approach using available EST, GSS and nr databases [23-26]. A recent study by Zhang et al. (2013) has led to the identification of 120 potato-specific miRNA families and 28 conserved miRNA families in leaf and stolon tissues by high-throughput sequencing [28]. However, majority of potato-specific miRNAs predicted by Zhang et al. (2013) were also detected in our sequencing dataset but could not meet the criteria for miRNA annotation employed in our analysis and thus excluded from further analysis.

To observe the expression profile of identified miRNAs, qRT-PCR was performed for 15 conserved and 6 potato-specific miRNAs. Our qRT-PCR results showed that majority of potato-specific (4) and conserved miRNAs (13) expressed at relatively high level in the stem tissues. This indicates that either these miRNAs express highly in the stem tissues or most of these miRNAs are being continuously transported through the conductive vascular tissues. In case of their high expression in stem it is possible that they might play an important role in stem development and differentiation. On the other hand, potato miR172 and other miRNAs (miR399, miR395) have been shown to be involved in graft transmissible movement through the conductive vascular tissues [20,47]. Interestingly, potato-specific miRNAs miRNA193, miRNA152 and the conserved miR172_1, miR172_5 showed significant expression during developmental stages of tuberization. This indicates a probable role for these miRNAs in potato tuberization. In our analysis, the expression profile of miR172 (known to be involved in potato tuberization) during developmental stages of tuberization was consistent with the previous report [20]. Also, we found that miR172_1 expressed at a high level in potato roots, which was not reported previously.

To investigate the functional importance of the identified miRNAs, targets were predicted using psRNA target finder server [40] and the annotated potato genome transcript data [19]. Among conserved miRNAs, miR164 was predicted to target mRNA encoding for NAC domain containing proteins. It has been also reported that miR164 is a key regulator in diverse developmental processes including floral, embryonic, vegetative organs and lateral root development [48,49]. PHAVOULTA like HD-ZIP III transcription factor was one of the predicted targets of miR166 in our study. Previous studies showed that miR166/miR165 targets HD-ZIP III transcription factor genes and regulate a wide range of developmental processes including meristem formation, vascular development, lateral organ polarity, root and nodule development [50-52]. In our analysis, miR159 and miR319 were predicted to target mRNA coding for GAMYB like transcription factor. GAMYB transcription factors are involved in GA signalling and it has been proven that miR159-mediated regulation of GAMYB affects plant anther development and flowering time in Arabidopsis [53]. Auxin response factor was found as a predicted target of miR160. It has been observed that miR160 targets ARF10, ARF16 and ARF17 and regulate various aspects of plant development in Arabidopsis [54]. Squamosa-promoter binding protein was predicted as the target of miR156 and miR157. Previous studies have demonstrated that miR156 plays a vital role in controlling leaf development, apical dominance, floral transition and development [55-57] by targeting several members of the Squamosa promoter binding protein like (SPL) family of plant-specific transcription factors. Interestingly, a recent study has shown that overexpression of miR156 in potato leads to development of stolon-borne aerial tubers from distal nodes [21]. The digital expression profile reported in this study also showed high abundance of miR156 in tuber developmental stages with highest level at PT0 (stolon development), suggesting a role of miR156 in the phase transition from basal lateral juvenile shoots to a storage organ in the process of tuber development. In our analysis, miR171 was found to target GRAS family transcription factors and HAIRY MERISTEM (a member of GRAS family transcription factor). They have been shown to be involved in regulating various aspects of plant growth and development [58]. In potato, our qRT-PCR data showed that all the three members of miR171 expressed highly in vegetative tissues (leaf and stem-miR171_2, stem and root-miR171_9, root-miR171_10), indicating a role of miR171 in regulating potato developmental process. Previous studies have indicated that miR172 largely targets gene encoding members of APETALA2 (AP2) and AP2-like transcription factor family and plays a major role in the regulation of flowering time, vegetative phase change and floral organ identity, by cleaving and repressing the translation of its target mRNAs [59-61]. Recently, miR172 has been shown to induce tuberization by regulating long distance signals and targeting *RAP1* mRNA (*Relative to APETALA2 1*) [20]. Our prediction analysis showed *RAP1*, which is a known target of miR172 in potato [20], as a predicted target of miR172. This data supports the efficacy of our target prediction analysis.

Further, miRNA-mediated cleavage of target of conserved miRNAs was confirmed by 5′ RLM-RACE. Several targets of conserved miRNAs have been validated using RACE analysis in tomato, pepper and rice [8,13,62]. However, this is the first study in potato where 5′ RLM-RACE was performed to authenticate predicted targets of conserved miRNAs. Our RACE results confirmed that transcripts of *AFR16, NAM, RAP1 and HAM* were cleaved *in vivo* by miR160, miR164, miR172 and miR171, respectively. For potato-specific miRNAs, degradome sequencing approach is being used for mapping of the cleavage site.

Our target prediction analysis in potato and other studies on plant species, especially rice and Arabidopsis, strongly suggest that most of the conserved miRNAs target plant transcription factors (SBP, NAC, ARF, GRAS and AP2) [2,9,41], thereby emphasizing the role of conserved miRNA in essential biological processes in plants, including potato tuberization.

GO was performed on potato-specific miRNAs targets to gain a better understanding of their possible function [44]. GO results revealed that majority of the targets are involved in oxidation-reduction processes, metabolic processes, regulation of transcription, transport, signal transduction and defense responses. These results suggest that they covered a broad range of physiological and metabolic processes. Thus, in contrast to conserved miRNA targets, most of the putative targets of potato-specific miRNAs were not transcription factors. A few potato-specific miRNAs were predicted to target transcripts encoding transcription factors, including NAC domain protein, GRAS family transcription factors, MADS-box family protein and ARF domain class transcription factor. These transcription factors are known to be targeted by conserved miRNAs and play a major role in various aspects of plant growth and development [2]. Thus, it appears that the potato-specific miRNAs might play important roles in essential biological processes as well. Future studies are essential to explain the functional significance of the inverse relationship between miRNA and their target which will improve our knowledge about the regulatory role of miRNAs in potato.

With respect to potato tuberization, expression profiling by qRT-PCR and target prediction studies indicated that a few miRNAs might play a role during transition from stolon to tuber development. qRT-PCR results indicated that miR172_1, miR172_5 (conserved miRNAs) and miRNA193, miRNA152 (potato-specific miRNAs) showed significant expression during developmental stages of tuberization. Lipoxygenase (LOX) was predicted as one of the targets of miRNA150, miRNA163, miRNA37, miRNA78 and miRNA108. Protein phosphatase type 2A (PP2A) was found to be a predicted target of miRNA90. Previous studies have suggested the role of miR172, LOX and PP2A in potato tuber developmental process [20,42,43]. Therefore, these potential miRNAs and their target genes are being further investigated to determine their roles in potato tuber development.

## Conclusions

In summary, we report a comprehensive study of potato miRNAs at genome-wide level using three different vegetative tissues and four early developmental stages of tuberization by high-throughput sequencing and high-end bioinformatics methods. We have identified a population of potato-specific miRNAs that could fill the gap in understanding the regulatory role of miRNAs in potato. The digital expression data was further quantified by qRT-PCR to determine up- and down-regulations of potato miRNAs in various vegetative tissues and developmental stages of tuberization. The putative target genes were predicted for conserved and potato-specific miRNAs to gain a better understanding of their function. Further, a few conserved miRNA targets were also validated by 5′ RLM-RACE and the predicted target genes for potato-specific miRNAs were subjected to GO annotation to infer the functional role of target genes.

## Methods

### Plant material

The plantlets of *Solanum tuberosum* (cv. Kufri Chandramukhi) were raised *in vitro* on MS medium [63] and maintained under the 16 hr light/8 hr dark photoperiod conditions at 22°C. The leaves, stem and root were collected from 45 days old whole *in vitro* grown potato plantlets and immediately frozen in liquid nitrogen, subsequently stored at -80°C until used. These *in vitro* grown plantlets were then potted and grown under natural field conditions, during the period of November-January, in the net house at the Department of Botany, University of Delhi, India. Tissues from the four early developmental stages of tuberization were harvested from these naturalized plants, frozen in liquid nitrogen and stored at -80°C until used.

### Total RNA isolation

Total RNA was isolated from the above mentioned tissues (0.2 grams each) using TRI reagent (Sigma, USA) as per the manufacturer’s instructions. The quality and quantity of RNA isolates was verified on denaturing agarose gel and by optical density (OD) measurement, respectively.

### Small RNA library construction

Small RNA libraries for each tissue type were prepared independently using an Illumina Small RNA sample prep kit (Illumina, U.S.A), according to manufacturer’s instructions. Briefly, small RNAs (20–40 nt) were separated on 15% denaturing polyacrylamide gel and purified. Next, small RNAs were ligated with the 5′ and 3′adapters sequentially, eluted and gel purified. The 5′and 3' ligated small RNAs were reverse transcribed using SuperScript II Reverse Transcriptase (Invitrogen, U.S.A) and PCR amplified with Phusion DNA Polymerase. The quality and quantity of cDNA library was assessed using Agilent Bioanalyzer (Agilent Technologies, U.S.A). Small RNA library preparation and their high-throughput sequencing was performed using an Illumina Genome Analyzer IIx (Illumina, U.S.A) at DBT-funded High Throughput Sequencing Facility at University of Delhi South Campus, India.

### Bioinformatic analysis of sRNA sequences

The removal of poor quality sequences and trimming of adaptor sequences from the raw sequence data was carried out using the UEA sRNA workbench 2.4 - Plant version sequence file pre-processing (http://srna-tools.cmp.uea.ac.uk/) [32]. Sequences, smaller than 16 nt and larger than 30 nt were also removed. High quality trimmed sequences (16–30 nt in length, reads with no “N”, no more than 4 bases with quality score <10 and no more than 6 bases with quality score <13) were mapped onto plant t/rRNAs sequences from “Rfam” (excepted miRNAs), Arabidopsis tRNAs from “The Genomic tRNA Database”, and plant t/rRNA sequences from “EMBL” release 95. The matched sequences were removed from downstream analysis. UEA sRNA toolkit-Plant version filter pipeline (http://srna-tools.cmp.uea.ac.uk/) was also used to remove any t/rRNA matchouts [32].

### Identification of conserved and potato-specific miRNAs

The unique reads were submitted to the UEA sRNA toolkit-Plant version miRCat pipeline (http://srna-tools.cmp.uea.ac.uk/) [32] and default criteria were used to predict potato-specific and conserved miRNAs in potato. The reads were aligned to the potato reference genome (PGSC DM assembly version 3) (http://potatogenomics.plantbiology.msu.edu/) with no mismatch. The 100 nt windows around the aligned reads were extracted from the genome and folded using RNAfold (http://rna.tbi.univie.ac.at/cgi-bin/RNAfold.cgi) [33] to identify precursor sequences. The resulting secondary structures were then trimmed and analysed by miRCat to confirm the characteristic miRNA hairpin structure. These potential precursors were then screened based on updated criteria for annotation of plant miRNAs developed by Meyers et al. [34] to recover the potential miRNA candidates - 1) The miRNA and miRNA* are derived from opposite stem-arms such that they form a duplex with two nucleotide 3′ overhangs 2) Base-pairing between the miRNA and miRNA* is extensive such that there are typically four or fewer mismatches. 3) The frequency of asymmetric bulges is one or none and size of the bulge is no more than 2 nucleotides within the miRNA/ miRNA* duplex. To identify the conserved miRNAs in potato, all the predicted miRNA sequences were mapped to known mature plant miRNAs deposited in miRBase v20 [35]. No more than 2 mismatches were allowed during the alignment. The remaining sequences that showed 3 or more than 3 mismatches or no homology to any previously known plant miRNAs were considered as novel or potato-specific miRNAs.

### Experimental validation of miRNAs by Quantitative Real-time PCR (qRT-PCR)

The expression level of miRNAs was analyzed using poly(T) adaptor RT-PCR method [64]. Total RNA was treated with RNase-free DNase I (NEB, England) and polyadenylated using Poly(A) Tailing kit (Ambion, U.S.A) at 37°C for 60 min in a 50 μl reaction volume containing 2 μg total RNA and 0.08 units poly (A) polymerase. The polyadenylated RNA was then reverse transcribed in a 20 μl reaction mix with SuperScript II Reverse Transcriptase (Invitrogen, U.S.A) and Poly(T) adaptor. Real time PCR was carried out on CFX Connect Real-Time System (Bio-Rad, U.S.A) using hydrolysis probe and probe fast chemistry (KAPA Biosystems, U.S.A). qRT-PCR was performed containing KAPA PROBE FAST Universal 1X qPCR Master Mix (KAPA Biosystems, U.S.A), 0.5 μl cDNA template (10X), 0.5 μM each of the miRNA specific forward primer and universal reverse primer and 0.2 μM universal hydrolysis probe. The cycling conditions were as follows - denaturation at 95°C for 5 min, followed by 40 cycles of denaturation at 95°C for 20 s, annealing and extension together at 60°C for 10 s. The PCR products were further run on 2% agarose gel to verify the presence of a single band of expected size. All the reactions were run with 3 biological replicates of each sample and 2 technical replicates of each biological replicate. Two controls (no template control and no Reverse Transcription control) were included for each miRNA. For normalization, 18S rRNA was used as an endogenous control. The quantitative PCR data was analyzed using 2^-∆∆Cq^ method where C_q_ is the threshold cycle [65]. The forward primers were designed taking full mature miRNA sequence. The detail of the primers, used in this study, is provided in Additional file 8. All the oligonucleotide sequences were synthesized from IDT (Integrated DNA technologies, U.S.A).

### Prediction of miRNA targets and GO analysis

Putative target genes of conserved and potato-specific miRNAs were predicted using psRNATarget: A plant small RNA target analysis server using default parameters [40]. The parameters were as follows - Maximum expectation: 3, Length for complementarity scoring: 20, Allowed maximum energy to unpair the target site (UPE): 25, Flanking length around target site for target accessibility analysis: 17 bp, Range of central mismatch leading to translation inhibition: 9–11 nt. Target with a low expectation score and UPE is considered to be highly potential target for miRNA. The transcript sequences of the potato genome (Group Phureja DM1-3 516R44) were used as a reference set. The putative target genes of the potato-specific miRNAs were further subjected to GO analysis to better understand their function. Blast2GO was used to investigate the GO of the identified potato-specific miRNA target [44].

### Target validation of conserved miRNAs by RLM-RACE

5′ RLM-RACE was used to determine the cleavage site of predicted targets. Briefly, polyA^+^ RNA was isolated using PolyATract mRNA Isolation System IV (Promega, USA). PolyA^+^ mRNA (25 ng) was ligated to RNA adapter provided in First Choice RLM-RACE kit (Ambion, USA) using T4 RNA ligase, followed by nested PCR. Gene-specific reverse primers and 5' RACE adapter specific primers were used for amplification of the target gene. The PCR products were eluted, cloned in pGEMT Easy vector (Promega, USA) and sequenced to map the cleavage site. The primers used for amplification were synthesized from IDT (Integrated DNA technologies, U.S.A) and is provided in Additional file 9.

### Availability of supporting data

“The data set supporting the results of this article are available in the Gene Expression Omnibus repository under accession no GSE52599 (http://www.ncbi.nlm.nih.gov/geo/query/acc.cgi?acc=GSE52599)”. The mature miRNA and precursor sequences will be submitted to miRBase registry and assigned final names after final acceptance of the manuscript.

## Competing interests

The authors declare that they have no competing interests.

## Authors’ contributions

AK conceived and designed the experiment, NL performed the experiments, GJ carried out the bioinformatics analysis, ARB constructed the sRNA libraries, MA and SK-A supervised the library preparation and sequencing experiments work, NL and AK wrote the manuscript, and all authors contributed towards improving and finalising the manuscript. All authors read and approved the final manuscript.

## Supplementary Material

Additional file 1 List of conserved miRNAs identified in potato in this study and their normalized reads (on the second sheet) in all the libraries. Click here for file

Additional file 2 List of potato-specific miRNAs identified in this study and their normalized reads (on the second sheet) in all the libraries. Click here for file

Additional file 3 List of candidate potato-specific miRNAs identified in this study and their normalized reads. Click here for file

Additional file 4 **Predicted secondary structures of pre-miRNAs of potato-specific miRNAs.** Secondary structures of precursors of potato-specific miRNAs were predicted using RNAfold. The mature sequence is highlighted with green colour while star sequence is highlighted with red colour. 5′end is marked by a circle. Click here for file

Additional file 5 Predicted targets for conserved miRNAs identified in this study. Click here for file

Additional file 6 Predicted targets for potato-specific miRNAs identified in this study. Click here for file

Additional file 7 **GO annotation and classification of predicted target genes of potato-specific miRNAs (on the first and second sheet).** The first sheet shows the number of genes in biological, metabolic and cellular processes. The second sheet shows the GO annotation of individual target gene. Click here for file

Additional file 8 List of primers used in this study for reverse transcription and qRT-PCR. Click here for file

Additional file 9 List of primers used in this study for 5′ RLM-RACE. Click here for file
