# Supplementary figures and images for: Identification and characterization of miRNAome in root, stem, leaf and tuber developmental stages of potato (Solanum tuberosum L.) by high-throughput sequencing (part 2 of 2)
Source: BMC Plant Biol. 2014 Jan 7;14:6. doi: 10.1186/1471-2229-14-6 (PMC3913621; doi:10.1186/1471-2229-14-6)

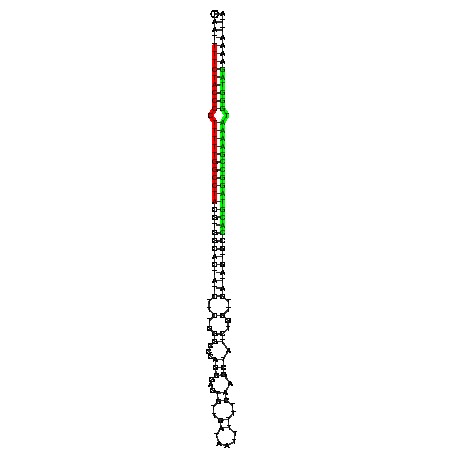

Supplement: Additional file 4 — Predicted secondary structures of pre-miRNAs of potato-specific miRNAs. Secondary structures of precursors of potato-specific miRNAs were predicted using RNAfold. The mature sequence is highlighted with green colour while star sequence is highlighted with red colour. 5′end is marked by a circle. [file 1471-2229-14-6-S4.zip › Additional file 4/miRNA 43.jpeg]

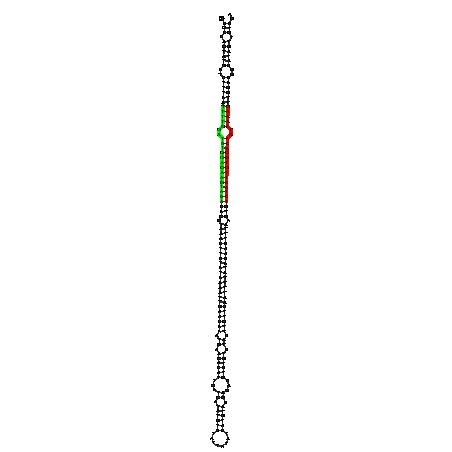

Supplement: Additional file 4 — Predicted secondary structures of pre-miRNAs of potato-specific miRNAs. Secondary structures of precursors of potato-specific miRNAs were predicted using RNAfold. The mature sequence is highlighted with green colour while star sequence is highlighted with red colour. 5′end is marked by a circle. [file 1471-2229-14-6-S4.zip › Additional file 4/miRNA 44.jpeg]

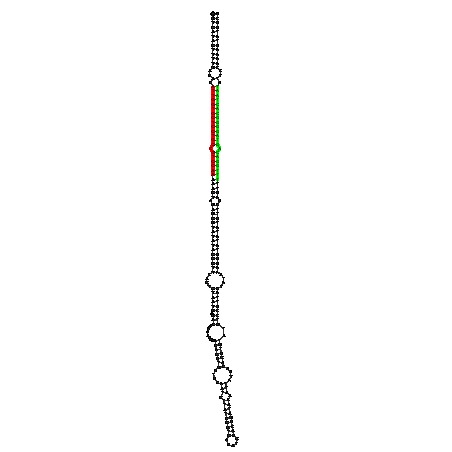

Supplement: Additional file 4 — Predicted secondary structures of pre-miRNAs of potato-specific miRNAs. Secondary structures of precursors of potato-specific miRNAs were predicted using RNAfold. The mature sequence is highlighted with green colour while star sequence is highlighted with red colour. 5′end is marked by a circle. [file 1471-2229-14-6-S4.zip › Additional file 4/miRNA 45.jpeg]

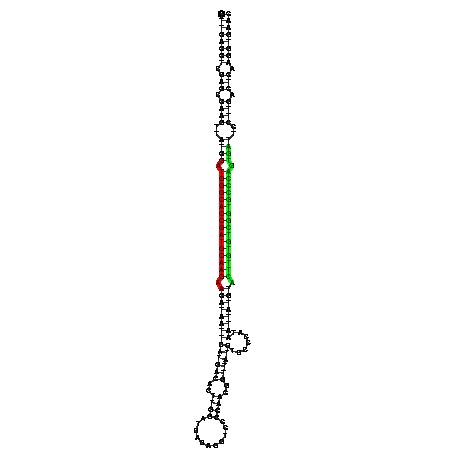

Supplement: Additional file 4 — Predicted secondary structures of pre-miRNAs of potato-specific miRNAs. Secondary structures of precursors of potato-specific miRNAs were predicted using RNAfold. The mature sequence is highlighted with green colour while star sequence is highlighted with red colour. 5′end is marked by a circle. [file 1471-2229-14-6-S4.zip › Additional file 4/miRNA 46.jpeg]

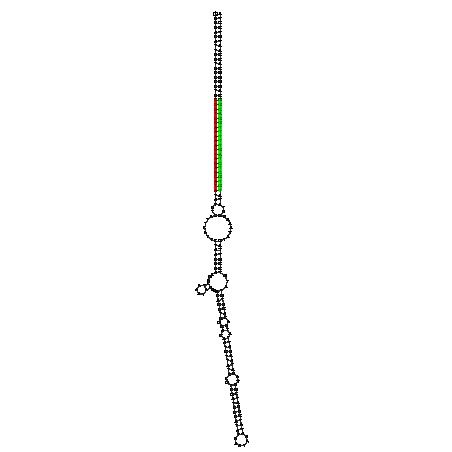

Supplement: Additional file 4 — Predicted secondary structures of pre-miRNAs of potato-specific miRNAs. Secondary structures of precursors of potato-specific miRNAs were predicted using RNAfold. The mature sequence is highlighted with green colour while star sequence is highlighted with red colour. 5′end is marked by a circle. [file 1471-2229-14-6-S4.zip › Additional file 4/miRNA 47.jpeg]

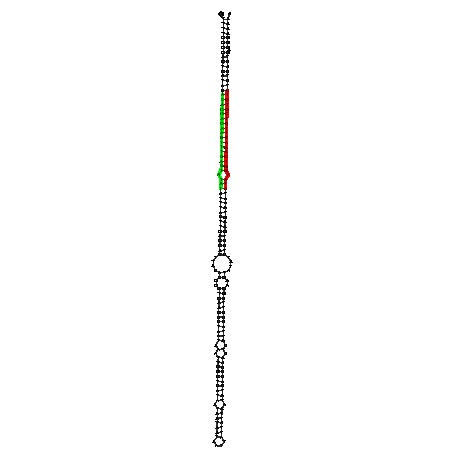

Supplement: Additional file 4 — Predicted secondary structures of pre-miRNAs of potato-specific miRNAs. Secondary structures of precursors of potato-specific miRNAs were predicted using RNAfold. The mature sequence is highlighted with green colour while star sequence is highlighted with red colour. 5′end is marked by a circle. [file 1471-2229-14-6-S4.zip › Additional file 4/miRNA 48.jpeg]

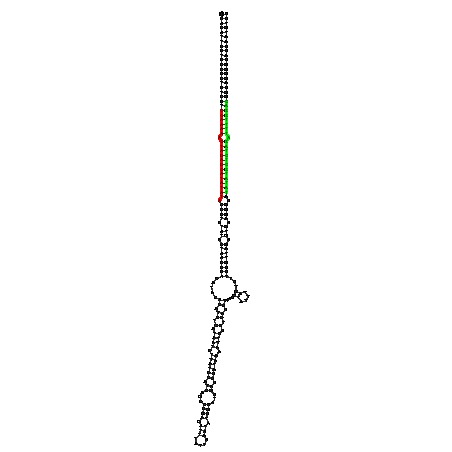

Supplement: Additional file 4 — Predicted secondary structures of pre-miRNAs of potato-specific miRNAs. Secondary structures of precursors of potato-specific miRNAs were predicted using RNAfold. The mature sequence is highlighted with green colour while star sequence is highlighted with red colour. 5′end is marked by a circle. [file 1471-2229-14-6-S4.zip › Additional file 4/miRNA 5.jpeg]

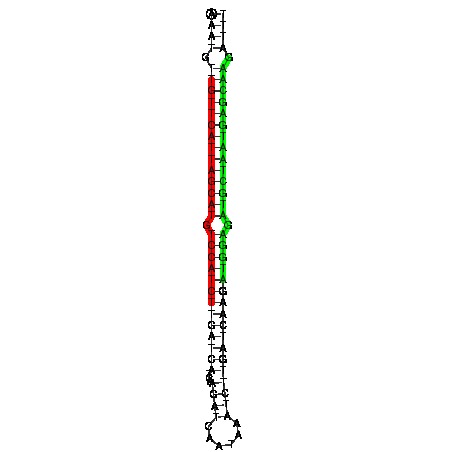

Supplement: Additional file 4 — Predicted secondary structures of pre-miRNAs of potato-specific miRNAs. Secondary structures of precursors of potato-specific miRNAs were predicted using RNAfold. The mature sequence is highlighted with green colour while star sequence is highlighted with red colour. 5′end is marked by a circle. [file 1471-2229-14-6-S4.zip › Additional file 4/miRNA 50.jpeg]

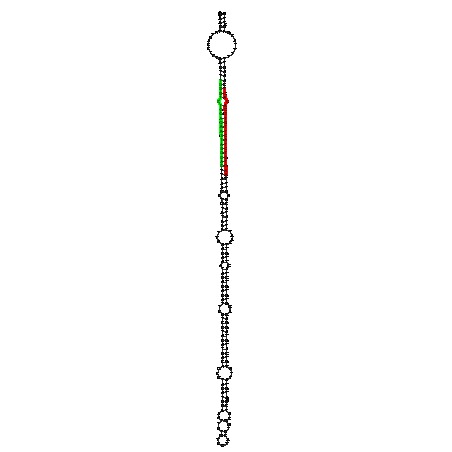

Supplement: Additional file 4 — Predicted secondary structures of pre-miRNAs of potato-specific miRNAs. Secondary structures of precursors of potato-specific miRNAs were predicted using RNAfold. The mature sequence is highlighted with green colour while star sequence is highlighted with red colour. 5′end is marked by a circle. [file 1471-2229-14-6-S4.zip › Additional file 4/miRNA 52.jpeg]

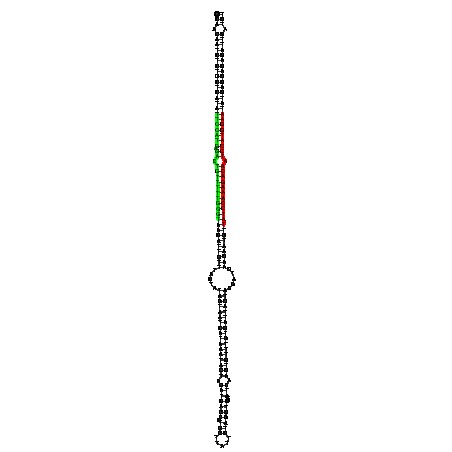

Supplement: Additional file 4 — Predicted secondary structures of pre-miRNAs of potato-specific miRNAs. Secondary structures of precursors of potato-specific miRNAs were predicted using RNAfold. The mature sequence is highlighted with green colour while star sequence is highlighted with red colour. 5′end is marked by a circle. [file 1471-2229-14-6-S4.zip › Additional file 4/miRNA 53.jpeg]

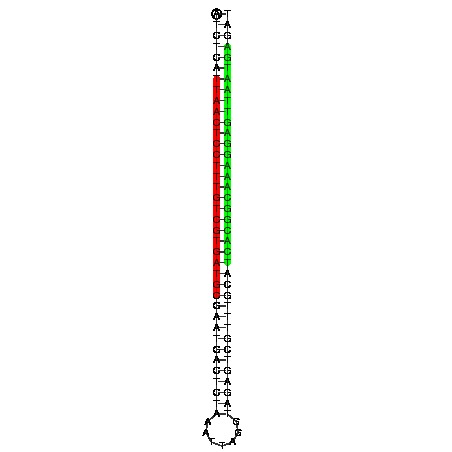

Supplement: Additional file 4 — Predicted secondary structures of pre-miRNAs of potato-specific miRNAs. Secondary structures of precursors of potato-specific miRNAs were predicted using RNAfold. The mature sequence is highlighted with green colour while star sequence is highlighted with red colour. 5′end is marked by a circle. [file 1471-2229-14-6-S4.zip › Additional file 4/miRNA 55.jpeg]

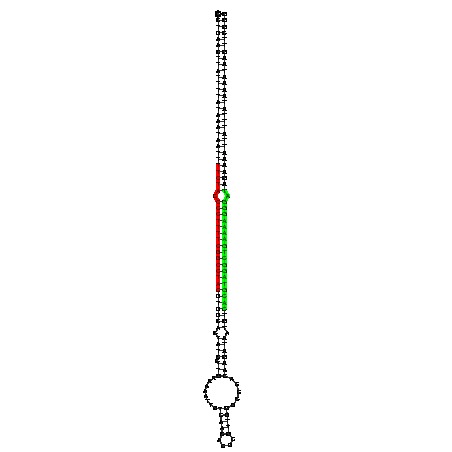

Supplement: Additional file 4 — Predicted secondary structures of pre-miRNAs of potato-specific miRNAs. Secondary structures of precursors of potato-specific miRNAs were predicted using RNAfold. The mature sequence is highlighted with green colour while star sequence is highlighted with red colour. 5′end is marked by a circle. [file 1471-2229-14-6-S4.zip › Additional file 4/miRNA 56.jpeg]

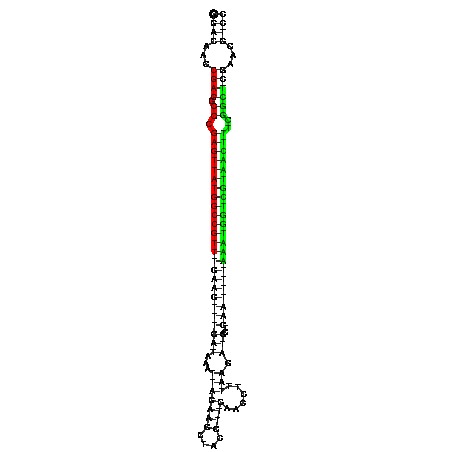

Supplement: Additional file 4 — Predicted secondary structures of pre-miRNAs of potato-specific miRNAs. Secondary structures of precursors of potato-specific miRNAs were predicted using RNAfold. The mature sequence is highlighted with green colour while star sequence is highlighted with red colour. 5′end is marked by a circle. [file 1471-2229-14-6-S4.zip › Additional file 4/miRNA 57.jpeg]

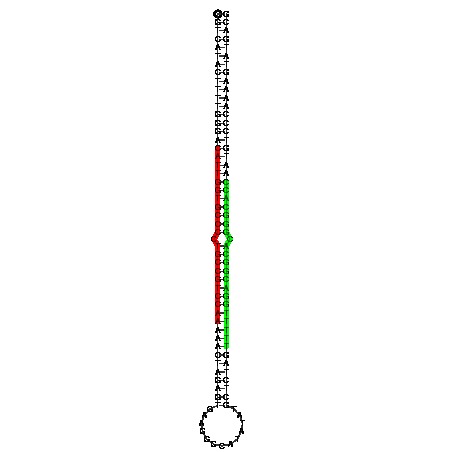

Supplement: Additional file 4 — Predicted secondary structures of pre-miRNAs of potato-specific miRNAs. Secondary structures of precursors of potato-specific miRNAs were predicted using RNAfold. The mature sequence is highlighted with green colour while star sequence is highlighted with red colour. 5′end is marked by a circle. [file 1471-2229-14-6-S4.zip › Additional file 4/miRNA 58.jpeg]

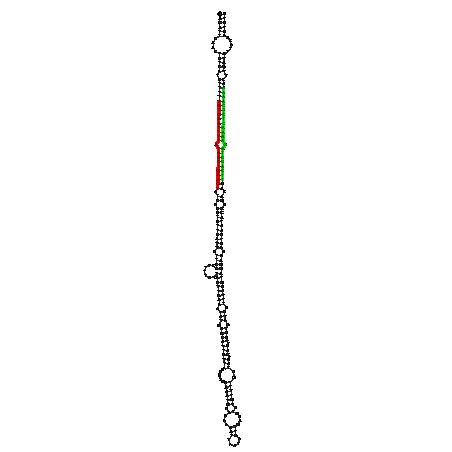

Supplement: Additional file 4 — Predicted secondary structures of pre-miRNAs of potato-specific miRNAs. Secondary structures of precursors of potato-specific miRNAs were predicted using RNAfold. The mature sequence is highlighted with green colour while star sequence is highlighted with red colour. 5′end is marked by a circle. [file 1471-2229-14-6-S4.zip › Additional file 4/miRNA 60.jpeg]

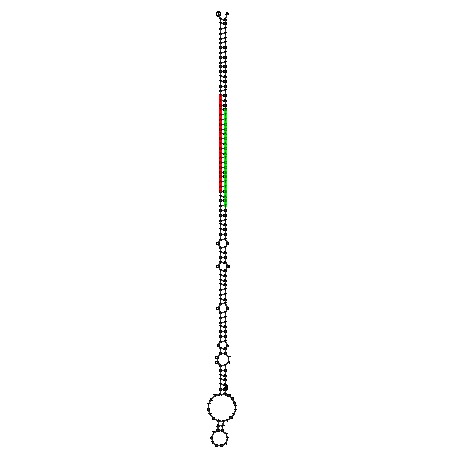

Supplement: Additional file 4 — Predicted secondary structures of pre-miRNAs of potato-specific miRNAs. Secondary structures of precursors of potato-specific miRNAs were predicted using RNAfold. The mature sequence is highlighted with green colour while star sequence is highlighted with red colour. 5′end is marked by a circle. [file 1471-2229-14-6-S4.zip › Additional file 4/miRNA 62.jpeg]

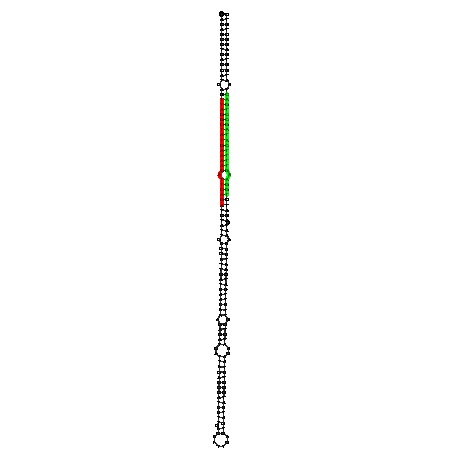

Supplement: Additional file 4 — Predicted secondary structures of pre-miRNAs of potato-specific miRNAs. Secondary structures of precursors of potato-specific miRNAs were predicted using RNAfold. The mature sequence is highlighted with green colour while star sequence is highlighted with red colour. 5′end is marked by a circle. [file 1471-2229-14-6-S4.zip › Additional file 4/miRNA 63.jpeg]

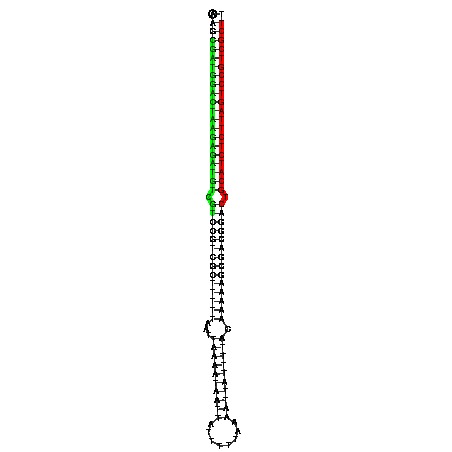

Supplement: Additional file 4 — Predicted secondary structures of pre-miRNAs of potato-specific miRNAs. Secondary structures of precursors of potato-specific miRNAs were predicted using RNAfold. The mature sequence is highlighted with green colour while star sequence is highlighted with red colour. 5′end is marked by a circle. [file 1471-2229-14-6-S4.zip › Additional file 4/miRNA 64.jpeg]

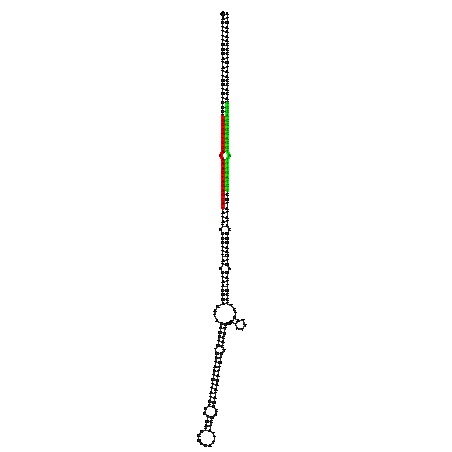

Supplement: Additional file 4 — Predicted secondary structures of pre-miRNAs of potato-specific miRNAs. Secondary structures of precursors of potato-specific miRNAs were predicted using RNAfold. The mature sequence is highlighted with green colour while star sequence is highlighted with red colour. 5′end is marked by a circle. [file 1471-2229-14-6-S4.zip › Additional file 4/miRNA 66.jpeg]

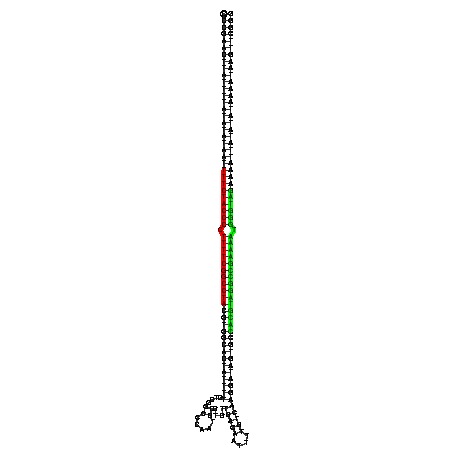

Supplement: Additional file 4 — Predicted secondary structures of pre-miRNAs of potato-specific miRNAs. Secondary structures of precursors of potato-specific miRNAs were predicted using RNAfold. The mature sequence is highlighted with green colour while star sequence is highlighted with red colour. 5′end is marked by a circle. [file 1471-2229-14-6-S4.zip › Additional file 4/miRNA 67.jpeg]

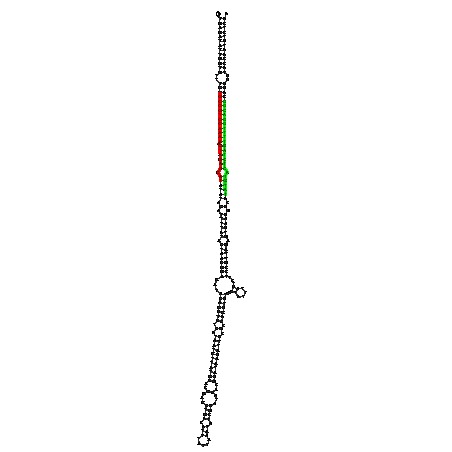

Supplement: Additional file 4 — Predicted secondary structures of pre-miRNAs of potato-specific miRNAs. Secondary structures of precursors of potato-specific miRNAs were predicted using RNAfold. The mature sequence is highlighted with green colour while star sequence is highlighted with red colour. 5′end is marked by a circle. [file 1471-2229-14-6-S4.zip › Additional file 4/miRNA 7.jpeg]

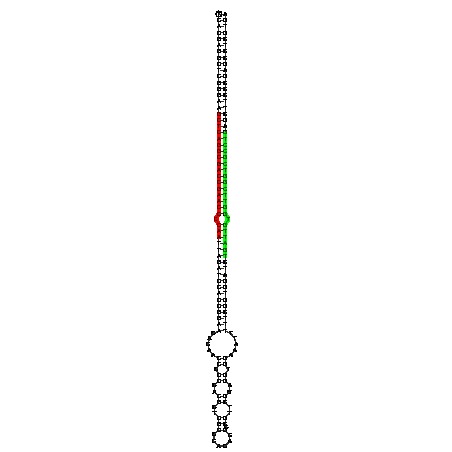

Supplement: Additional file 4 — Predicted secondary structures of pre-miRNAs of potato-specific miRNAs. Secondary structures of precursors of potato-specific miRNAs were predicted using RNAfold. The mature sequence is highlighted with green colour while star sequence is highlighted with red colour. 5′end is marked by a circle. [file 1471-2229-14-6-S4.zip › Additional file 4/miRNA 70.jpeg]

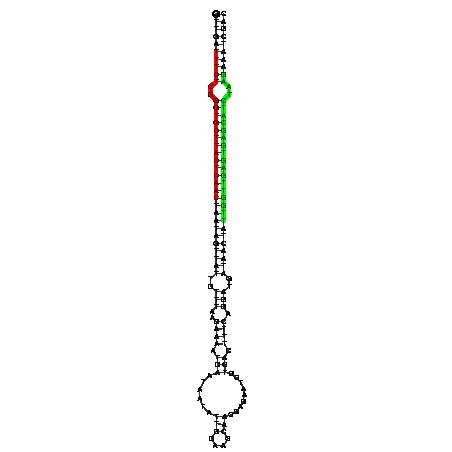

Supplement: Additional file 4 — Predicted secondary structures of pre-miRNAs of potato-specific miRNAs. Secondary structures of precursors of potato-specific miRNAs were predicted using RNAfold. The mature sequence is highlighted with green colour while star sequence is highlighted with red colour. 5′end is marked by a circle. [file 1471-2229-14-6-S4.zip › Additional file 4/miRNA 72.jpeg]

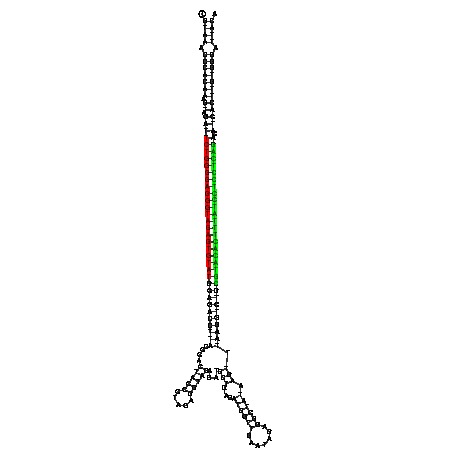

Supplement: Additional file 4 — Predicted secondary structures of pre-miRNAs of potato-specific miRNAs. Secondary structures of precursors of potato-specific miRNAs were predicted using RNAfold. The mature sequence is highlighted with green colour while star sequence is highlighted with red colour. 5′end is marked by a circle. [file 1471-2229-14-6-S4.zip › Additional file 4/miRNA 74.jpeg]

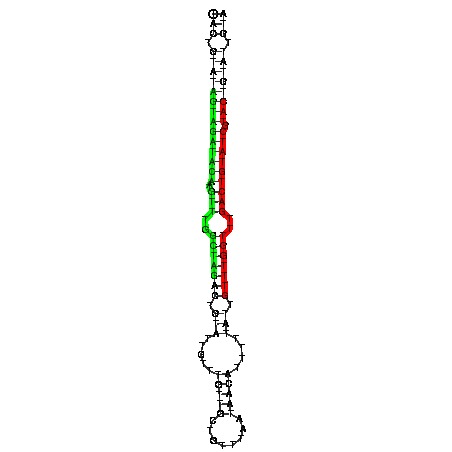

Supplement: Additional file 4 — Predicted secondary structures of pre-miRNAs of potato-specific miRNAs. Secondary structures of precursors of potato-specific miRNAs were predicted using RNAfold. The mature sequence is highlighted with green colour while star sequence is highlighted with red colour. 5′end is marked by a circle. [file 1471-2229-14-6-S4.zip › Additional file 4/miRNA 75.jpeg]

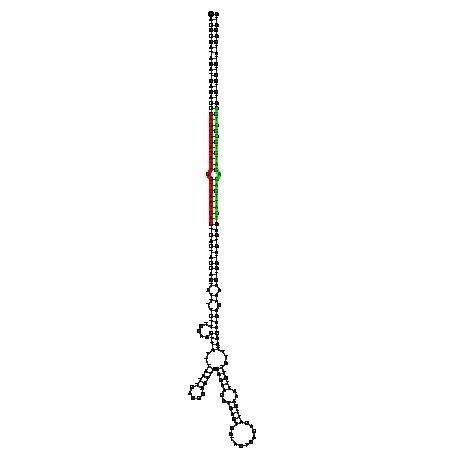

Supplement: Additional file 4 — Predicted secondary structures of pre-miRNAs of potato-specific miRNAs. Secondary structures of precursors of potato-specific miRNAs were predicted using RNAfold. The mature sequence is highlighted with green colour while star sequence is highlighted with red colour. 5′end is marked by a circle. [file 1471-2229-14-6-S4.zip › Additional file 4/miRNA 76.jpeg]

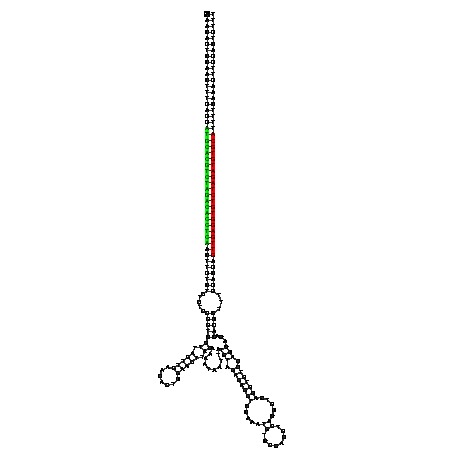

Supplement: Additional file 4 — Predicted secondary structures of pre-miRNAs of potato-specific miRNAs. Secondary structures of precursors of potato-specific miRNAs were predicted using RNAfold. The mature sequence is highlighted with green colour while star sequence is highlighted with red colour. 5′end is marked by a circle. [file 1471-2229-14-6-S4.zip › Additional file 4/miRNA 77.jpeg]

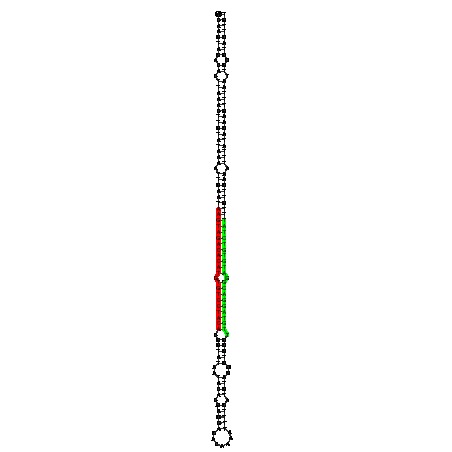

Supplement: Additional file 4 — Predicted secondary structures of pre-miRNAs of potato-specific miRNAs. Secondary structures of precursors of potato-specific miRNAs were predicted using RNAfold. The mature sequence is highlighted with green colour while star sequence is highlighted with red colour. 5′end is marked by a circle. [file 1471-2229-14-6-S4.zip › Additional file 4/miRNA 78.jpeg]

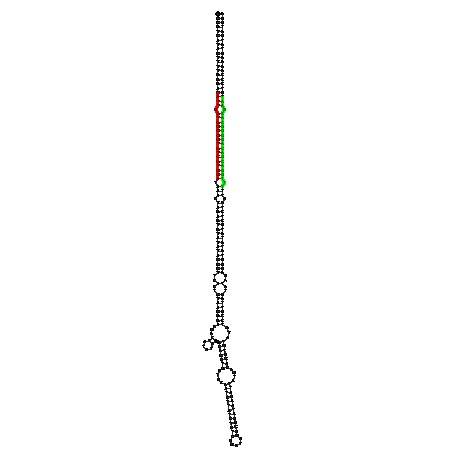

Supplement: Additional file 4 — Predicted secondary structures of pre-miRNAs of potato-specific miRNAs. Secondary structures of precursors of potato-specific miRNAs were predicted using RNAfold. The mature sequence is highlighted with green colour while star sequence is highlighted with red colour. 5′end is marked by a circle. [file 1471-2229-14-6-S4.zip › Additional file 4/miRNA 79.jpeg]

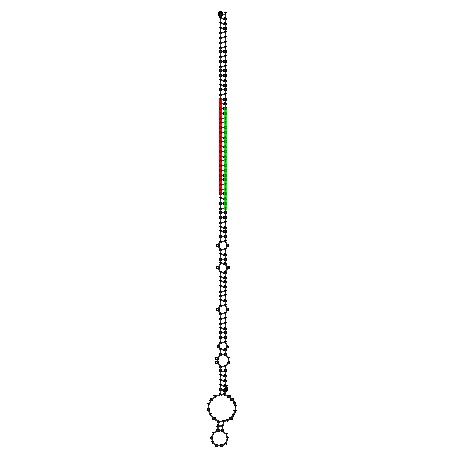

Supplement: Additional file 4 — Predicted secondary structures of pre-miRNAs of potato-specific miRNAs. Secondary structures of precursors of potato-specific miRNAs were predicted using RNAfold. The mature sequence is highlighted with green colour while star sequence is highlighted with red colour. 5′end is marked by a circle. [file 1471-2229-14-6-S4.zip › Additional file 4/miRNA 80.jpeg]

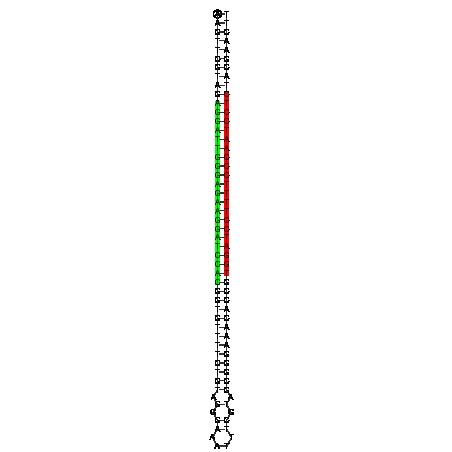

Supplement: Additional file 4 — Predicted secondary structures of pre-miRNAs of potato-specific miRNAs. Secondary structures of precursors of potato-specific miRNAs were predicted using RNAfold. The mature sequence is highlighted with green colour while star sequence is highlighted with red colour. 5′end is marked by a circle. [file 1471-2229-14-6-S4.zip › Additional file 4/miRNA 82.jpeg]

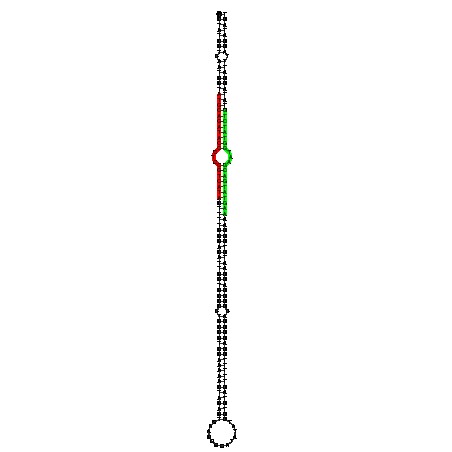

Supplement: Additional file 4 — Predicted secondary structures of pre-miRNAs of potato-specific miRNAs. Secondary structures of precursors of potato-specific miRNAs were predicted using RNAfold. The mature sequence is highlighted with green colour while star sequence is highlighted with red colour. 5′end is marked by a circle. [file 1471-2229-14-6-S4.zip › Additional file 4/miRNA 85.jpeg]

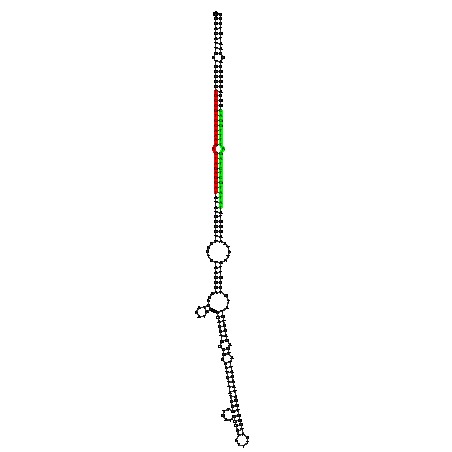

Supplement: Additional file 4 — Predicted secondary structures of pre-miRNAs of potato-specific miRNAs. Secondary structures of precursors of potato-specific miRNAs were predicted using RNAfold. The mature sequence is highlighted with green colour while star sequence is highlighted with red colour. 5′end is marked by a circle. [file 1471-2229-14-6-S4.zip › Additional file 4/miRNA 87.jpeg]

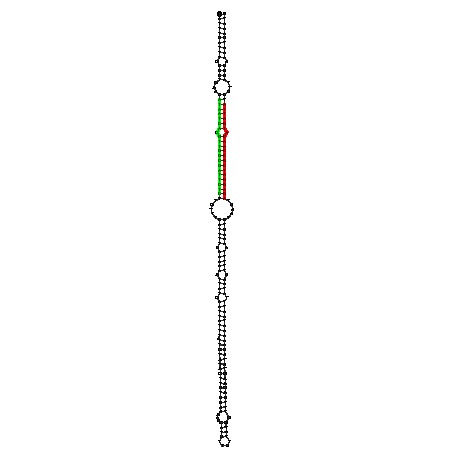

Supplement: Additional file 4 — Predicted secondary structures of pre-miRNAs of potato-specific miRNAs. Secondary structures of precursors of potato-specific miRNAs were predicted using RNAfold. The mature sequence is highlighted with green colour while star sequence is highlighted with red colour. 5′end is marked by a circle. [file 1471-2229-14-6-S4.zip › Additional file 4/miRNA 88.jpeg]

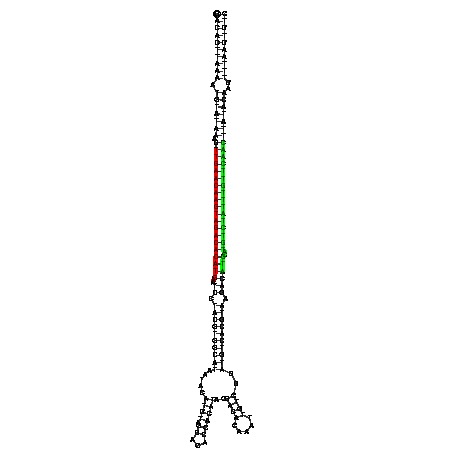

Supplement: Additional file 4 — Predicted secondary structures of pre-miRNAs of potato-specific miRNAs. Secondary structures of precursors of potato-specific miRNAs were predicted using RNAfold. The mature sequence is highlighted with green colour while star sequence is highlighted with red colour. 5′end is marked by a circle. [file 1471-2229-14-6-S4.zip › Additional file 4/miRNA 90.jpeg]

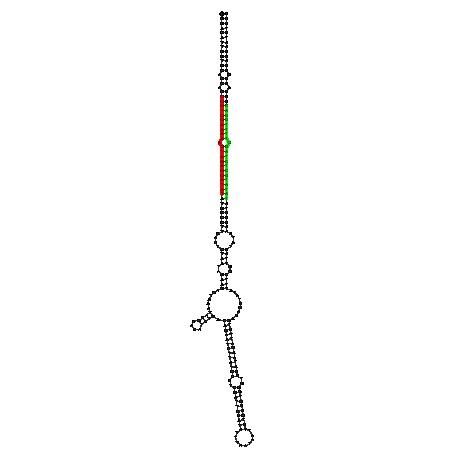

Supplement: Additional file 4 — Predicted secondary structures of pre-miRNAs of potato-specific miRNAs. Secondary structures of precursors of potato-specific miRNAs were predicted using RNAfold. The mature sequence is highlighted with green colour while star sequence is highlighted with red colour. 5′end is marked by a circle. [file 1471-2229-14-6-S4.zip › Additional file 4/miRNA 91.jpeg]

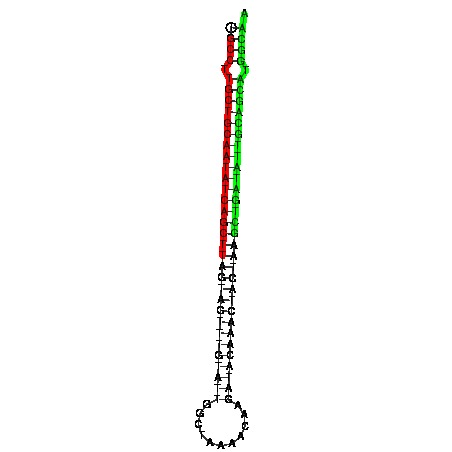

Supplement: Additional file 4 — Predicted secondary structures of pre-miRNAs of potato-specific miRNAs. Secondary structures of precursors of potato-specific miRNAs were predicted using RNAfold. The mature sequence is highlighted with green colour while star sequence is highlighted with red colour. 5′end is marked by a circle. [file 1471-2229-14-6-S4.zip › Additional file 4/miRNA 92.jpeg]

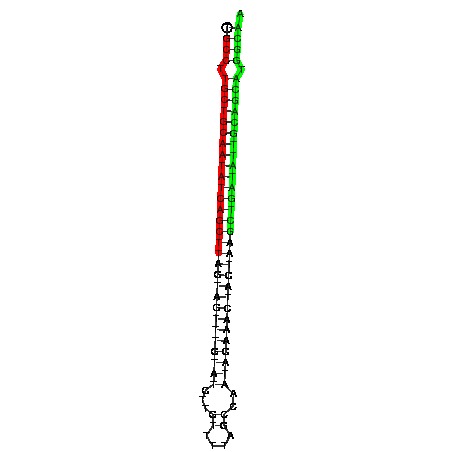

Supplement: Additional file 4 — Predicted secondary structures of pre-miRNAs of potato-specific miRNAs. Secondary structures of precursors of potato-specific miRNAs were predicted using RNAfold. The mature sequence is highlighted with green colour while star sequence is highlighted with red colour. 5′end is marked by a circle. [file 1471-2229-14-6-S4.zip › Additional file 4/miRNA 93.jpeg]

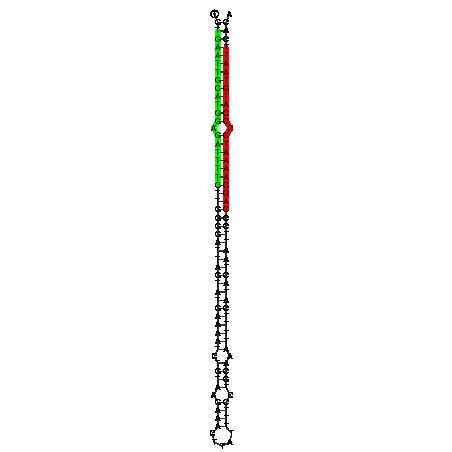

Supplement: Additional file 4 — Predicted secondary structures of pre-miRNAs of potato-specific miRNAs. Secondary structures of precursors of potato-specific miRNAs were predicted using RNAfold. The mature sequence is highlighted with green colour while star sequence is highlighted with red colour. 5′end is marked by a circle. [file 1471-2229-14-6-S4.zip › Additional file 4/miRNA 94.jpeg]

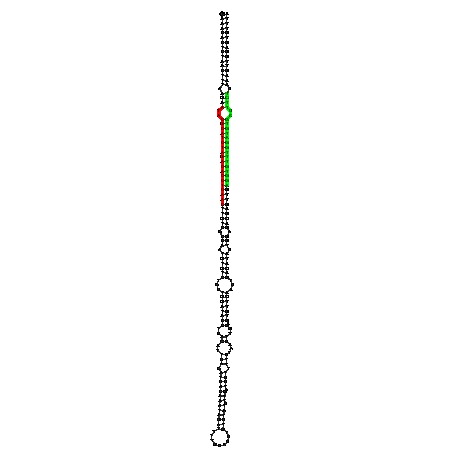

Supplement: Additional file 4 — Predicted secondary structures of pre-miRNAs of potato-specific miRNAs. Secondary structures of precursors of potato-specific miRNAs were predicted using RNAfold. The mature sequence is highlighted with green colour while star sequence is highlighted with red colour. 5′end is marked by a circle. [file 1471-2229-14-6-S4.zip › Additional file 4/miRNA 95.jpeg]

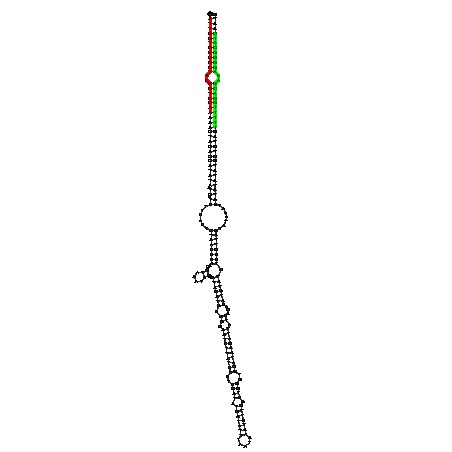

Supplement: Additional file 4 — Predicted secondary structures of pre-miRNAs of potato-specific miRNAs. Secondary structures of precursors of potato-specific miRNAs were predicted using RNAfold. The mature sequence is highlighted with green colour while star sequence is highlighted with red colour. 5′end is marked by a circle. [file 1471-2229-14-6-S4.zip › Additional file 4/miRNA 97.jpeg]

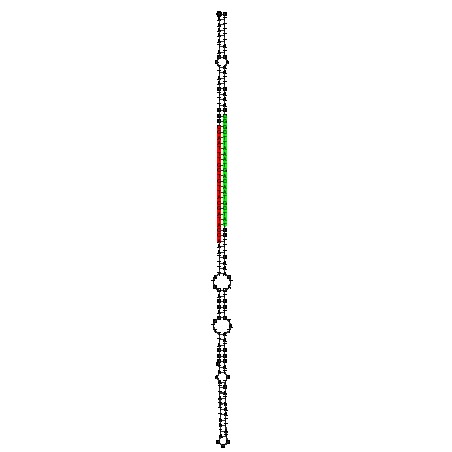

Supplement: Additional file 4 — Predicted secondary structures of pre-miRNAs of potato-specific miRNAs. Secondary structures of precursors of potato-specific miRNAs were predicted using RNAfold. The mature sequence is highlighted with green colour while star sequence is highlighted with red colour. 5′end is marked by a circle. [file 1471-2229-14-6-S4.zip › Additional file 4/miRNA 98.jpeg]

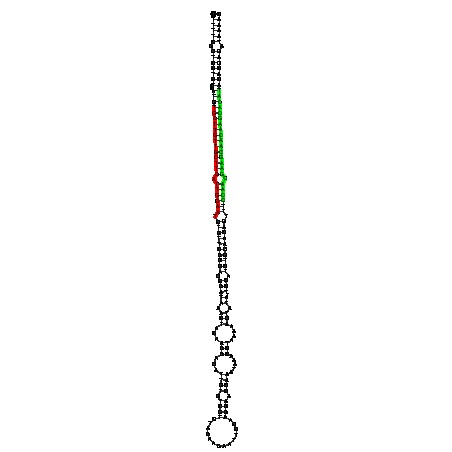

Supplement: Additional file 4 — Predicted secondary structures of pre-miRNAs of potato-specific miRNAs. Secondary structures of precursors of potato-specific miRNAs were predicted using RNAfold. The mature sequence is highlighted with green colour while star sequence is highlighted with red colour. 5′end is marked by a circle. [file 1471-2229-14-6-S4.zip › Additional file 4/miRNA 99.jpeg]

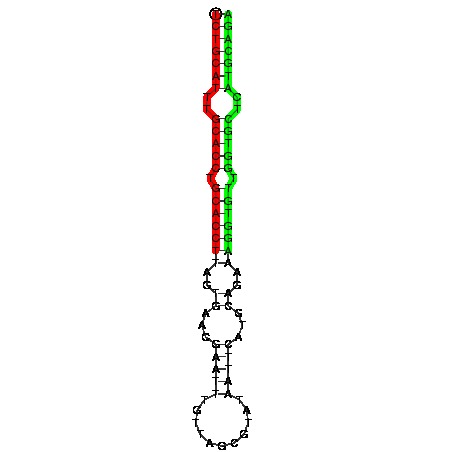

Supplement: Additional file 4 — Predicted secondary structures of pre-miRNAs of potato-specific miRNAs. Secondary structures of precursors of potato-specific miRNAs were predicted using RNAfold. The mature sequence is highlighted with green colour while star sequence is highlighted with red colour. 5′end is marked by a circle. [file 1471-2229-14-6-S4.zip › Additional file 4/miRNA153.jpeg]

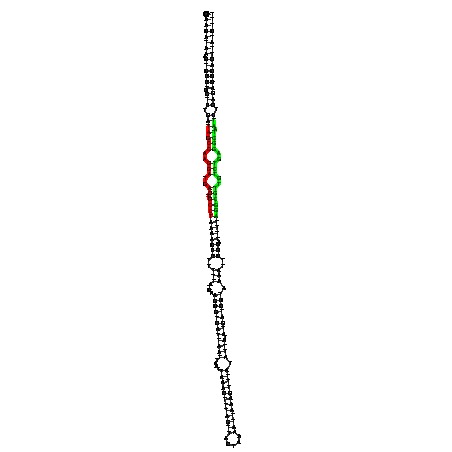

Supplement: Additional file 4 — Predicted secondary structures of pre-miRNAs of potato-specific miRNAs. Secondary structures of precursors of potato-specific miRNAs were predicted using RNAfold. The mature sequence is highlighted with green colour while star sequence is highlighted with red colour. 5′end is marked by a circle. [file 1471-2229-14-6-S4.zip › Additional file 4/miRNA154.jpeg]

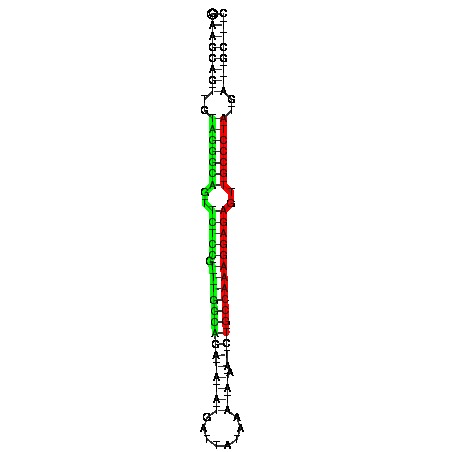

Supplement: Additional file 4 — Predicted secondary structures of pre-miRNAs of potato-specific miRNAs. Secondary structures of precursors of potato-specific miRNAs were predicted using RNAfold. The mature sequence is highlighted with green colour while star sequence is highlighted with red colour. 5′end is marked by a circle. [file 1471-2229-14-6-S4.zip › Additional file 4/miRNA42.jpeg]

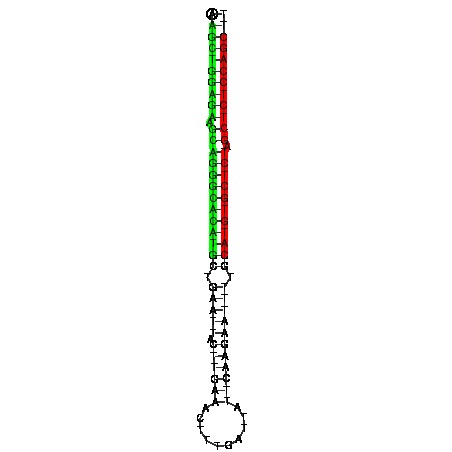

Supplement: Additional file 4 — Predicted secondary structures of pre-miRNAs of potato-specific miRNAs. Secondary structures of precursors of potato-specific miRNAs were predicted using RNAfold. The mature sequence is highlighted with green colour while star sequence is highlighted with red colour. 5′end is marked by a circle. [file 1471-2229-14-6-S4.zip › Additional file 4/miRNA123.jpeg]
