# Supplementary figures and images for: Identification and characterization of miRNAome in root, stem, leaf and tuber developmental stages of potato (Solanum tuberosum L.) by high-throughput sequencing (part 1 of 2)
Source: BMC Plant Biol. 2014 Jan 7;14:6. doi: 10.1186/1471-2229-14-6 (PMC3913621; doi:10.1186/1471-2229-14-6)

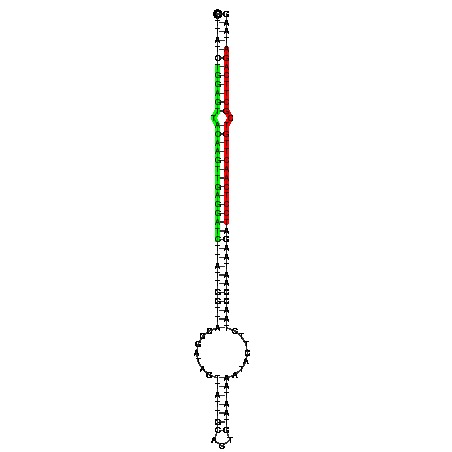

Supplement: Additional file 4 — Predicted secondary structures of pre-miRNAs of potato-specific miRNAs. Secondary structures of precursors of potato-specific miRNAs were predicted using RNAfold. The mature sequence is highlighted with green colour while star sequence is highlighted with red colour. 5′end is marked by a circle. [file 1471-2229-14-6-S4.zip › Additional file 4/miRNA 1.jpeg]

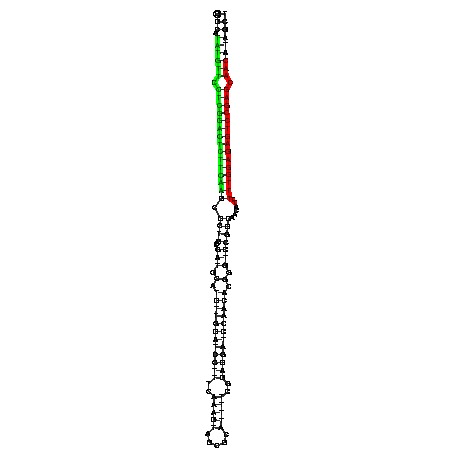

Supplement: Additional file 4 — Predicted secondary structures of pre-miRNAs of potato-specific miRNAs. Secondary structures of precursors of potato-specific miRNAs were predicted using RNAfold. The mature sequence is highlighted with green colour while star sequence is highlighted with red colour. 5′end is marked by a circle. [file 1471-2229-14-6-S4.zip › Additional file 4/miRNA 100.jpeg]

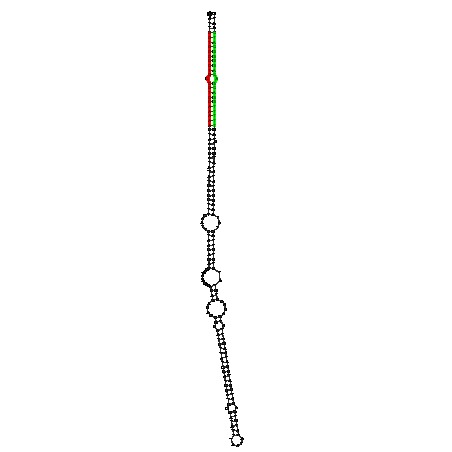

Supplement: Additional file 4 — Predicted secondary structures of pre-miRNAs of potato-specific miRNAs. Secondary structures of precursors of potato-specific miRNAs were predicted using RNAfold. The mature sequence is highlighted with green colour while star sequence is highlighted with red colour. 5′end is marked by a circle. [file 1471-2229-14-6-S4.zip › Additional file 4/miRNA 101.jpeg]

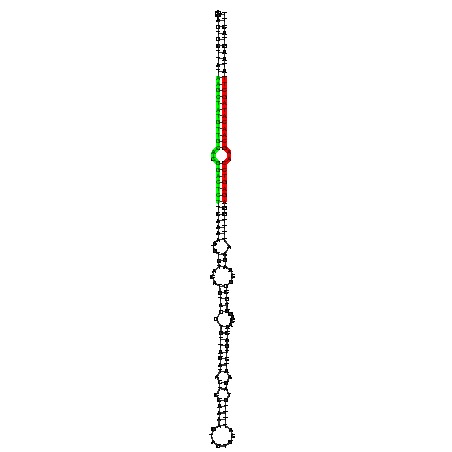

Supplement: Additional file 4 — Predicted secondary structures of pre-miRNAs of potato-specific miRNAs. Secondary structures of precursors of potato-specific miRNAs were predicted using RNAfold. The mature sequence is highlighted with green colour while star sequence is highlighted with red colour. 5′end is marked by a circle. [file 1471-2229-14-6-S4.zip › Additional file 4/miRNA 102.jpeg]

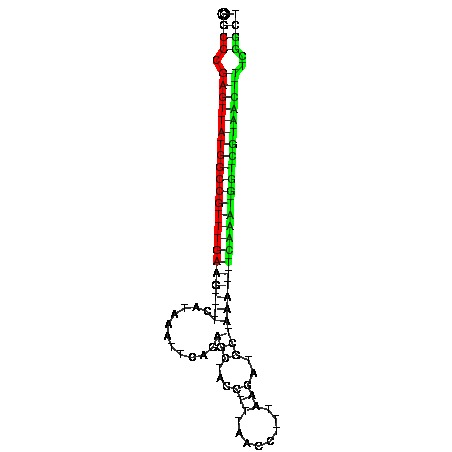

Supplement: Additional file 4 — Predicted secondary structures of pre-miRNAs of potato-specific miRNAs. Secondary structures of precursors of potato-specific miRNAs were predicted using RNAfold. The mature sequence is highlighted with green colour while star sequence is highlighted with red colour. 5′end is marked by a circle. [file 1471-2229-14-6-S4.zip › Additional file 4/miRNA 103.jpeg]

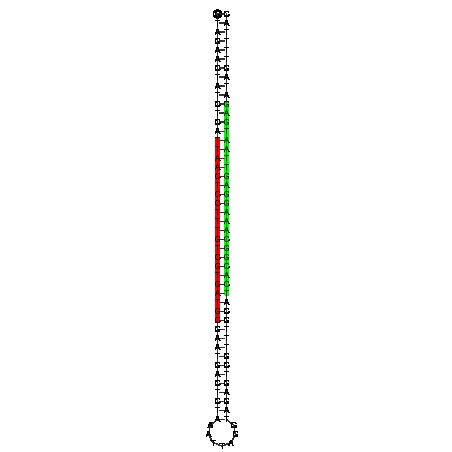

Supplement: Additional file 4 — Predicted secondary structures of pre-miRNAs of potato-specific miRNAs. Secondary structures of precursors of potato-specific miRNAs were predicted using RNAfold. The mature sequence is highlighted with green colour while star sequence is highlighted with red colour. 5′end is marked by a circle. [file 1471-2229-14-6-S4.zip › Additional file 4/miRNA 107.jpeg]

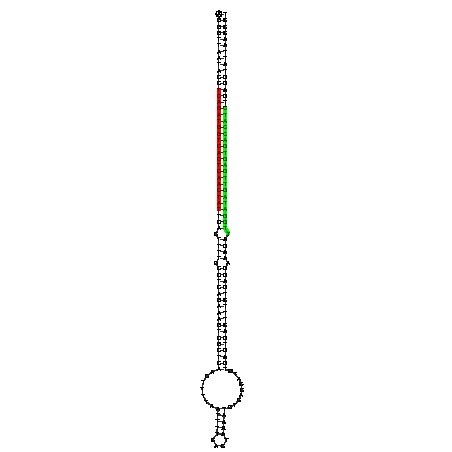

Supplement: Additional file 4 — Predicted secondary structures of pre-miRNAs of potato-specific miRNAs. Secondary structures of precursors of potato-specific miRNAs were predicted using RNAfold. The mature sequence is highlighted with green colour while star sequence is highlighted with red colour. 5′end is marked by a circle. [file 1471-2229-14-6-S4.zip › Additional file 4/miRNA 108.jpeg]

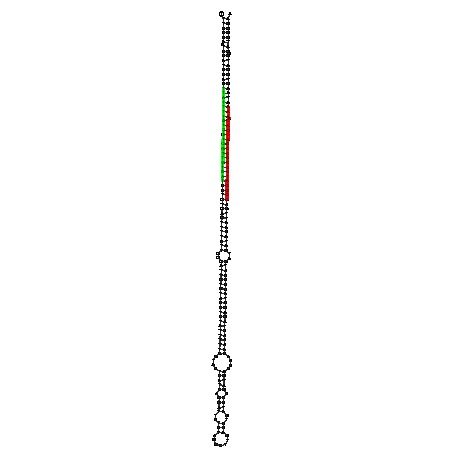

Supplement: Additional file 4 — Predicted secondary structures of pre-miRNAs of potato-specific miRNAs. Secondary structures of precursors of potato-specific miRNAs were predicted using RNAfold. The mature sequence is highlighted with green colour while star sequence is highlighted with red colour. 5′end is marked by a circle. [file 1471-2229-14-6-S4.zip › Additional file 4/miRNA 109.jpeg]

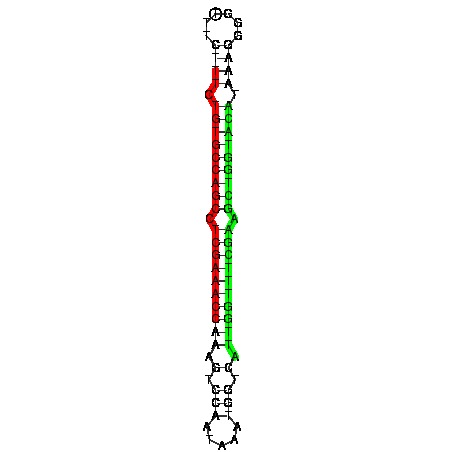

Supplement: Additional file 4 — Predicted secondary structures of pre-miRNAs of potato-specific miRNAs. Secondary structures of precursors of potato-specific miRNAs were predicted using RNAfold. The mature sequence is highlighted with green colour while star sequence is highlighted with red colour. 5′end is marked by a circle. [file 1471-2229-14-6-S4.zip › Additional file 4/miRNA 110.jpeg]

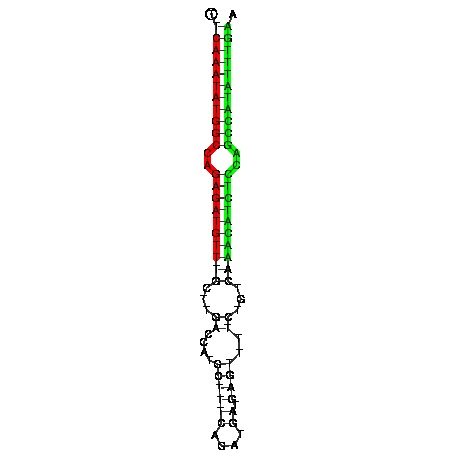

Supplement: Additional file 4 — Predicted secondary structures of pre-miRNAs of potato-specific miRNAs. Secondary structures of precursors of potato-specific miRNAs were predicted using RNAfold. The mature sequence is highlighted with green colour while star sequence is highlighted with red colour. 5′end is marked by a circle. [file 1471-2229-14-6-S4.zip › Additional file 4/miRNA 111.jpeg]

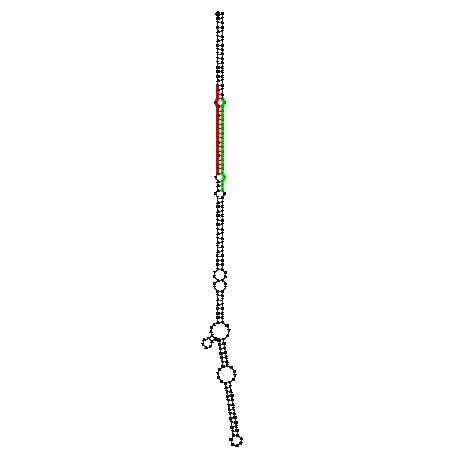

Supplement: Additional file 4 — Predicted secondary structures of pre-miRNAs of potato-specific miRNAs. Secondary structures of precursors of potato-specific miRNAs were predicted using RNAfold. The mature sequence is highlighted with green colour while star sequence is highlighted with red colour. 5′end is marked by a circle. [file 1471-2229-14-6-S4.zip › Additional file 4/miRNA 112.jpeg]

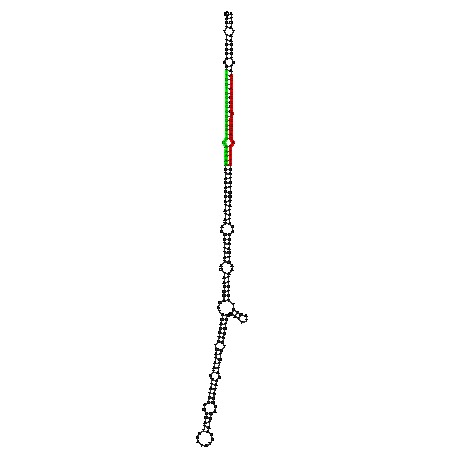

Supplement: Additional file 4 — Predicted secondary structures of pre-miRNAs of potato-specific miRNAs. Secondary structures of precursors of potato-specific miRNAs were predicted using RNAfold. The mature sequence is highlighted with green colour while star sequence is highlighted with red colour. 5′end is marked by a circle. [file 1471-2229-14-6-S4.zip › Additional file 4/miRNA 113.jpeg]

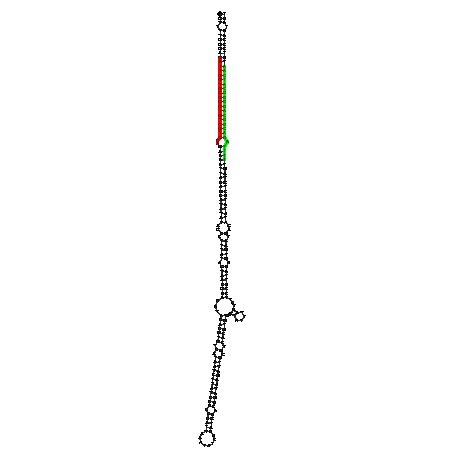

Supplement: Additional file 4 — Predicted secondary structures of pre-miRNAs of potato-specific miRNAs. Secondary structures of precursors of potato-specific miRNAs were predicted using RNAfold. The mature sequence is highlighted with green colour while star sequence is highlighted with red colour. 5′end is marked by a circle. [file 1471-2229-14-6-S4.zip › Additional file 4/miRNA 117.jpeg]

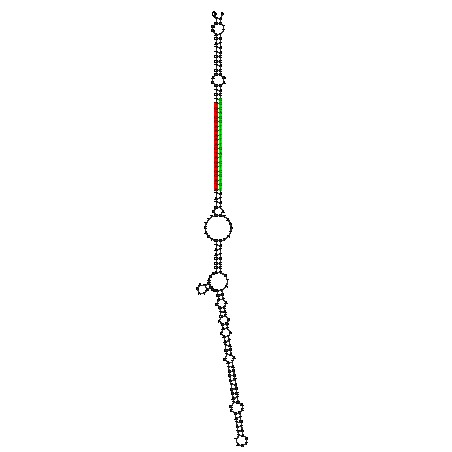

Supplement: Additional file 4 — Predicted secondary structures of pre-miRNAs of potato-specific miRNAs. Secondary structures of precursors of potato-specific miRNAs were predicted using RNAfold. The mature sequence is highlighted with green colour while star sequence is highlighted with red colour. 5′end is marked by a circle. [file 1471-2229-14-6-S4.zip › Additional file 4/miRNA 119.jpeg]

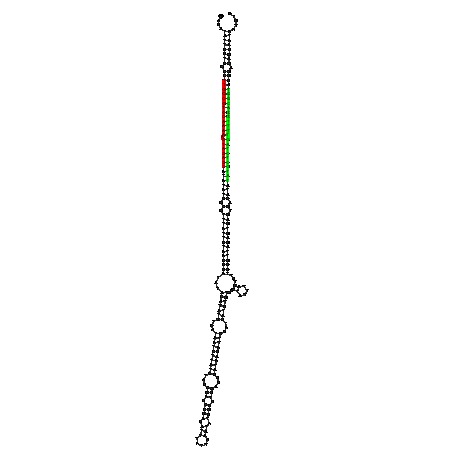

Supplement: Additional file 4 — Predicted secondary structures of pre-miRNAs of potato-specific miRNAs. Secondary structures of precursors of potato-specific miRNAs were predicted using RNAfold. The mature sequence is highlighted with green colour while star sequence is highlighted with red colour. 5′end is marked by a circle. [file 1471-2229-14-6-S4.zip › Additional file 4/miRNA 12.jpeg]

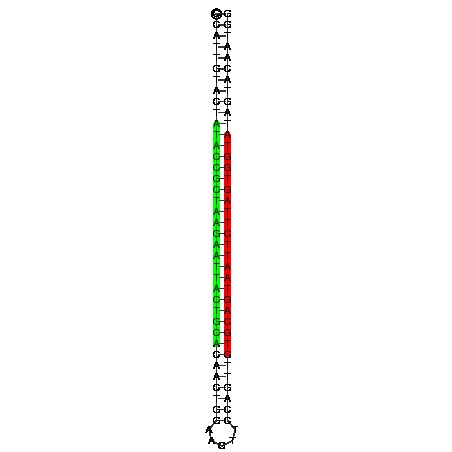

Supplement: Additional file 4 — Predicted secondary structures of pre-miRNAs of potato-specific miRNAs. Secondary structures of precursors of potato-specific miRNAs were predicted using RNAfold. The mature sequence is highlighted with green colour while star sequence is highlighted with red colour. 5′end is marked by a circle. [file 1471-2229-14-6-S4.zip › Additional file 4/miRNA 121.jpeg]

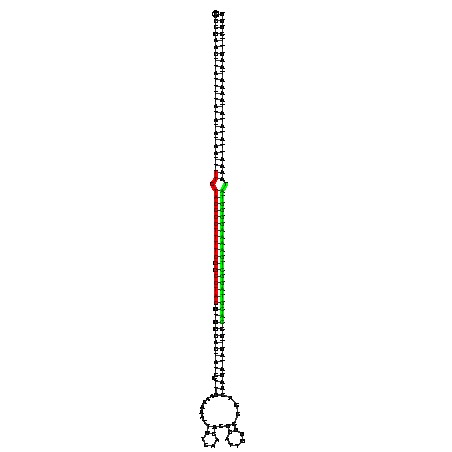

Supplement: Additional file 4 — Predicted secondary structures of pre-miRNAs of potato-specific miRNAs. Secondary structures of precursors of potato-specific miRNAs were predicted using RNAfold. The mature sequence is highlighted with green colour while star sequence is highlighted with red colour. 5′end is marked by a circle. [file 1471-2229-14-6-S4.zip › Additional file 4/miRNA 122.jpeg]

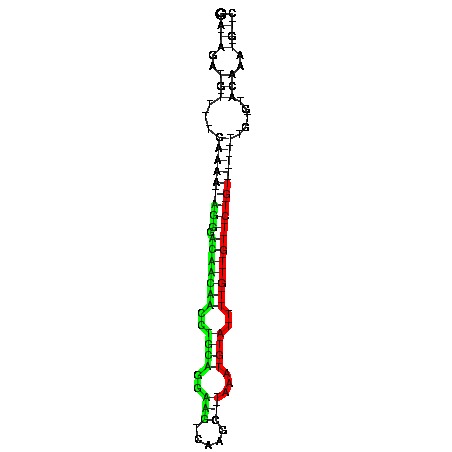

Supplement: Additional file 4 — Predicted secondary structures of pre-miRNAs of potato-specific miRNAs. Secondary structures of precursors of potato-specific miRNAs were predicted using RNAfold. The mature sequence is highlighted with green colour while star sequence is highlighted with red colour. 5′end is marked by a circle. [file 1471-2229-14-6-S4.zip › Additional file 4/miRNA 124.jpeg]

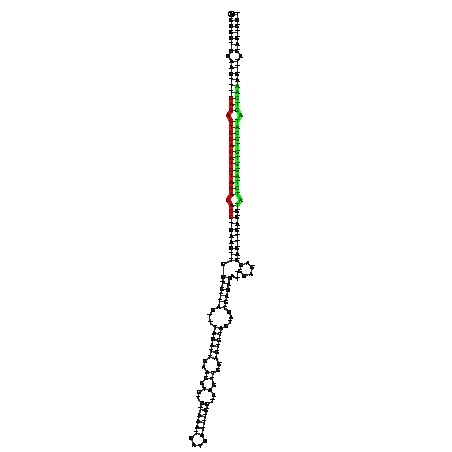

Supplement: Additional file 4 — Predicted secondary structures of pre-miRNAs of potato-specific miRNAs. Secondary structures of precursors of potato-specific miRNAs were predicted using RNAfold. The mature sequence is highlighted with green colour while star sequence is highlighted with red colour. 5′end is marked by a circle. [file 1471-2229-14-6-S4.zip › Additional file 4/miRNA 126.jpeg]

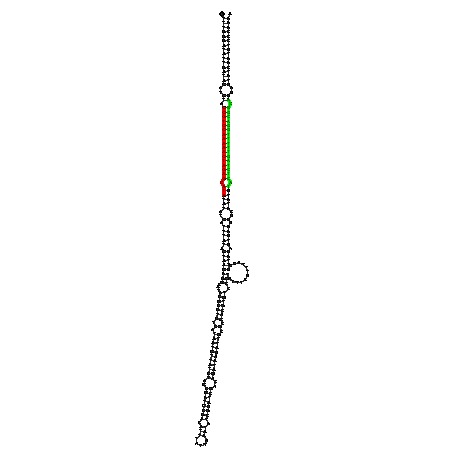

Supplement: Additional file 4 — Predicted secondary structures of pre-miRNAs of potato-specific miRNAs. Secondary structures of precursors of potato-specific miRNAs were predicted using RNAfold. The mature sequence is highlighted with green colour while star sequence is highlighted with red colour. 5′end is marked by a circle. [file 1471-2229-14-6-S4.zip › Additional file 4/miRNA 132.jpeg]

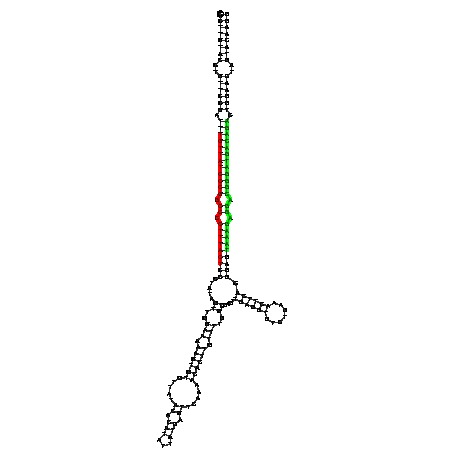

Supplement: Additional file 4 — Predicted secondary structures of pre-miRNAs of potato-specific miRNAs. Secondary structures of precursors of potato-specific miRNAs were predicted using RNAfold. The mature sequence is highlighted with green colour while star sequence is highlighted with red colour. 5′end is marked by a circle. [file 1471-2229-14-6-S4.zip › Additional file 4/miRNA 134.jpeg]

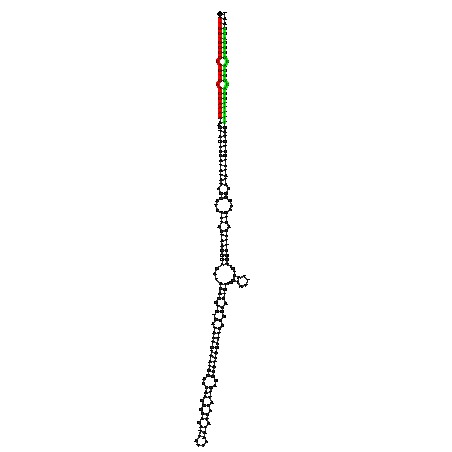

Supplement: Additional file 4 — Predicted secondary structures of pre-miRNAs of potato-specific miRNAs. Secondary structures of precursors of potato-specific miRNAs were predicted using RNAfold. The mature sequence is highlighted with green colour while star sequence is highlighted with red colour. 5′end is marked by a circle. [file 1471-2229-14-6-S4.zip › Additional file 4/miRNA 135.jpeg]

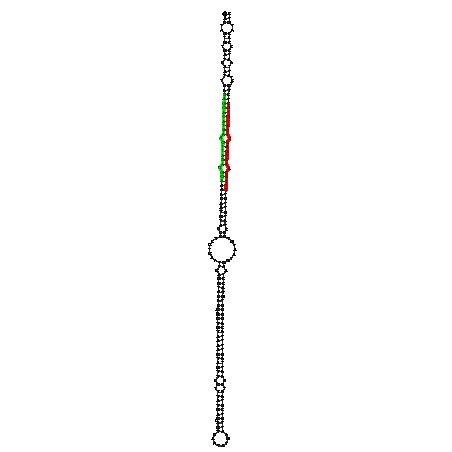

Supplement: Additional file 4 — Predicted secondary structures of pre-miRNAs of potato-specific miRNAs. Secondary structures of precursors of potato-specific miRNAs were predicted using RNAfold. The mature sequence is highlighted with green colour while star sequence is highlighted with red colour. 5′end is marked by a circle. [file 1471-2229-14-6-S4.zip › Additional file 4/miRNA 136.jpeg]

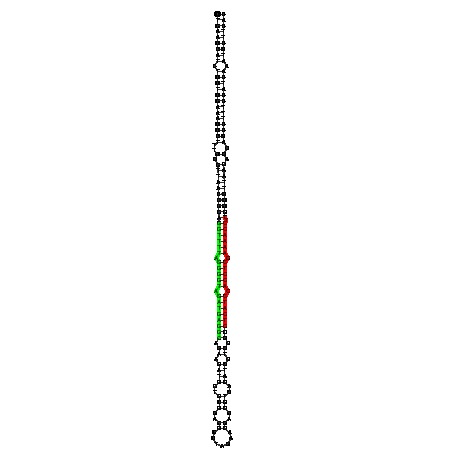

Supplement: Additional file 4 — Predicted secondary structures of pre-miRNAs of potato-specific miRNAs. Secondary structures of precursors of potato-specific miRNAs were predicted using RNAfold. The mature sequence is highlighted with green colour while star sequence is highlighted with red colour. 5′end is marked by a circle. [file 1471-2229-14-6-S4.zip › Additional file 4/miRNA 137.jpeg]

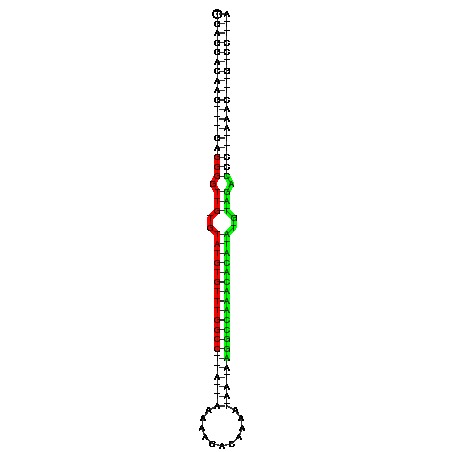

Supplement: Additional file 4 — Predicted secondary structures of pre-miRNAs of potato-specific miRNAs. Secondary structures of precursors of potato-specific miRNAs were predicted using RNAfold. The mature sequence is highlighted with green colour while star sequence is highlighted with red colour. 5′end is marked by a circle. [file 1471-2229-14-6-S4.zip › Additional file 4/miRNA 144.jpeg]

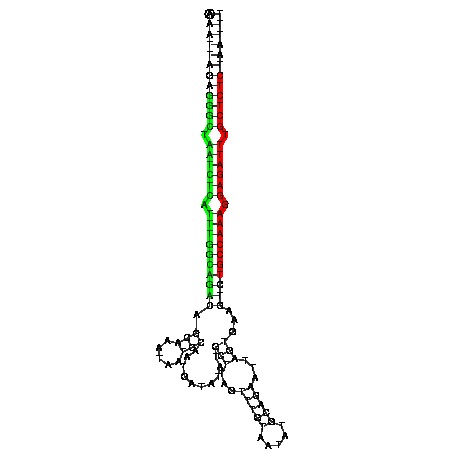

Supplement: Additional file 4 — Predicted secondary structures of pre-miRNAs of potato-specific miRNAs. Secondary structures of precursors of potato-specific miRNAs were predicted using RNAfold. The mature sequence is highlighted with green colour while star sequence is highlighted with red colour. 5′end is marked by a circle. [file 1471-2229-14-6-S4.zip › Additional file 4/miRNA 145.jpeg]

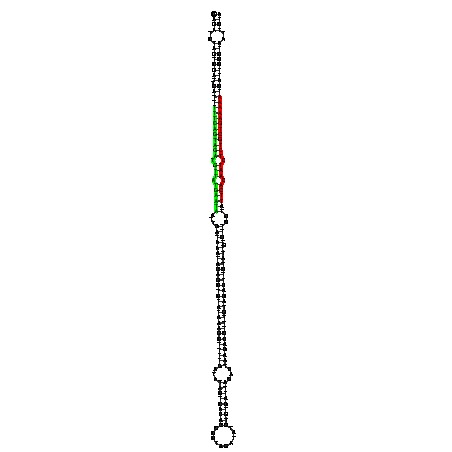

Supplement: Additional file 4 — Predicted secondary structures of pre-miRNAs of potato-specific miRNAs. Secondary structures of precursors of potato-specific miRNAs were predicted using RNAfold. The mature sequence is highlighted with green colour while star sequence is highlighted with red colour. 5′end is marked by a circle. [file 1471-2229-14-6-S4.zip › Additional file 4/miRNA 146.jpeg]

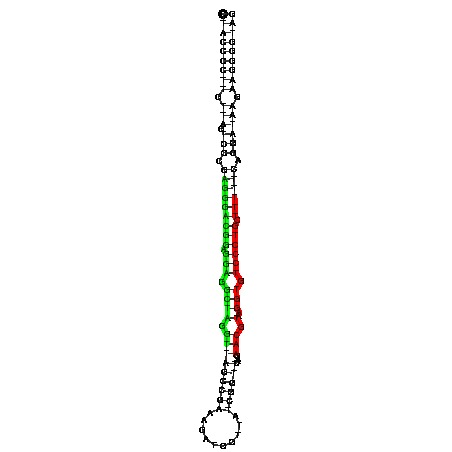

Supplement: Additional file 4 — Predicted secondary structures of pre-miRNAs of potato-specific miRNAs. Secondary structures of precursors of potato-specific miRNAs were predicted using RNAfold. The mature sequence is highlighted with green colour while star sequence is highlighted with red colour. 5′end is marked by a circle. [file 1471-2229-14-6-S4.zip › Additional file 4/miRNA 147.jpeg]

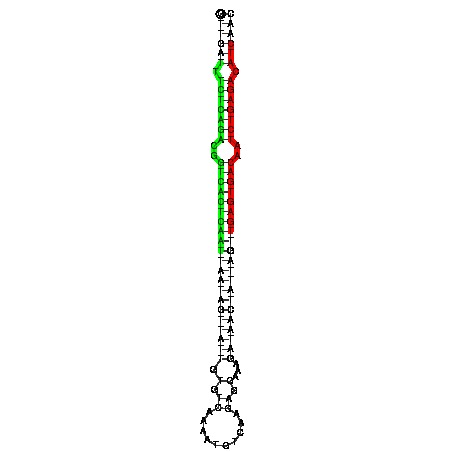

Supplement: Additional file 4 — Predicted secondary structures of pre-miRNAs of potato-specific miRNAs. Secondary structures of precursors of potato-specific miRNAs were predicted using RNAfold. The mature sequence is highlighted with green colour while star sequence is highlighted with red colour. 5′end is marked by a circle. [file 1471-2229-14-6-S4.zip › Additional file 4/miRNA 148.jpeg]

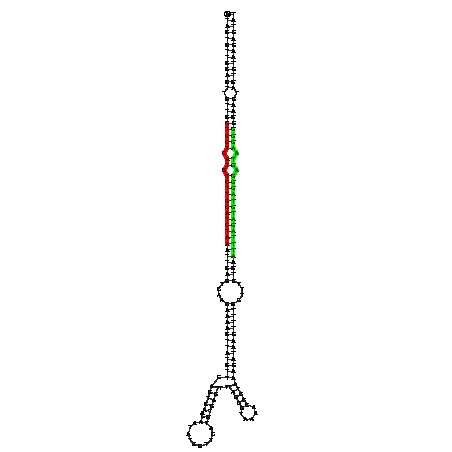

Supplement: Additional file 4 — Predicted secondary structures of pre-miRNAs of potato-specific miRNAs. Secondary structures of precursors of potato-specific miRNAs were predicted using RNAfold. The mature sequence is highlighted with green colour while star sequence is highlighted with red colour. 5′end is marked by a circle. [file 1471-2229-14-6-S4.zip › Additional file 4/miRNA 149.jpeg]

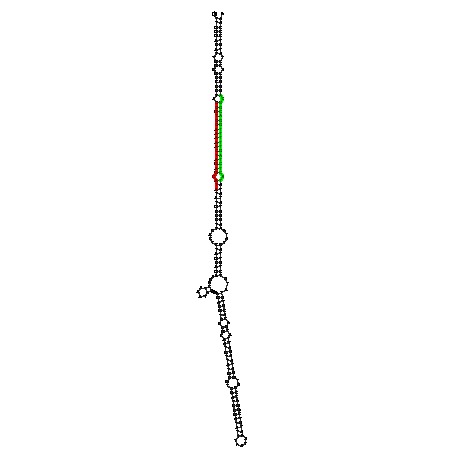

Supplement: Additional file 4 — Predicted secondary structures of pre-miRNAs of potato-specific miRNAs. Secondary structures of precursors of potato-specific miRNAs were predicted using RNAfold. The mature sequence is highlighted with green colour while star sequence is highlighted with red colour. 5′end is marked by a circle. [file 1471-2229-14-6-S4.zip › Additional file 4/miRNA 15.jpeg]

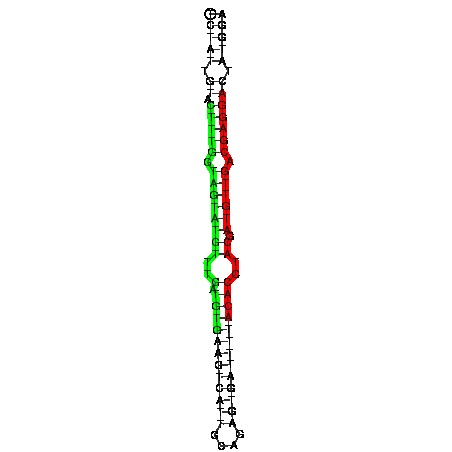

Supplement: Additional file 4 — Predicted secondary structures of pre-miRNAs of potato-specific miRNAs. Secondary structures of precursors of potato-specific miRNAs were predicted using RNAfold. The mature sequence is highlighted with green colour while star sequence is highlighted with red colour. 5′end is marked by a circle. [file 1471-2229-14-6-S4.zip › Additional file 4/miRNA 150.jpeg]

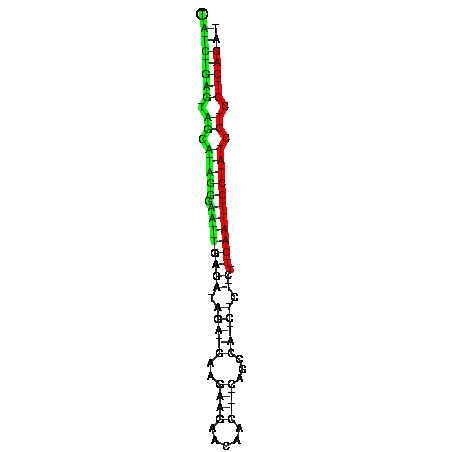

Supplement: Additional file 4 — Predicted secondary structures of pre-miRNAs of potato-specific miRNAs. Secondary structures of precursors of potato-specific miRNAs were predicted using RNAfold. The mature sequence is highlighted with green colour while star sequence is highlighted with red colour. 5′end is marked by a circle. [file 1471-2229-14-6-S4.zip › Additional file 4/miRNA 152.jpeg]

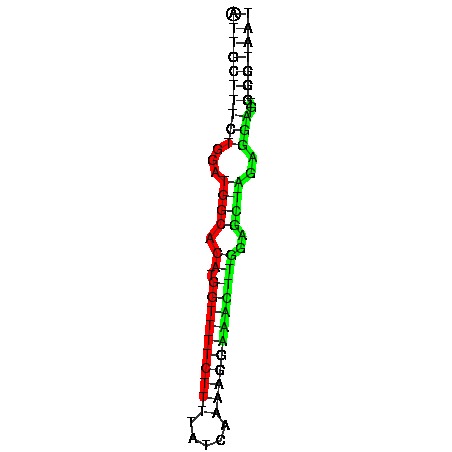

Supplement: Additional file 4 — Predicted secondary structures of pre-miRNAs of potato-specific miRNAs. Secondary structures of precursors of potato-specific miRNAs were predicted using RNAfold. The mature sequence is highlighted with green colour while star sequence is highlighted with red colour. 5′end is marked by a circle. [file 1471-2229-14-6-S4.zip › Additional file 4/miRNA 157.jpeg]

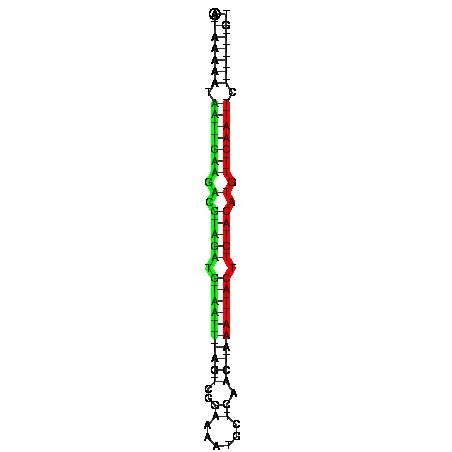

Supplement: Additional file 4 — Predicted secondary structures of pre-miRNAs of potato-specific miRNAs. Secondary structures of precursors of potato-specific miRNAs were predicted using RNAfold. The mature sequence is highlighted with green colour while star sequence is highlighted with red colour. 5′end is marked by a circle. [file 1471-2229-14-6-S4.zip › Additional file 4/miRNA 159.jpeg]

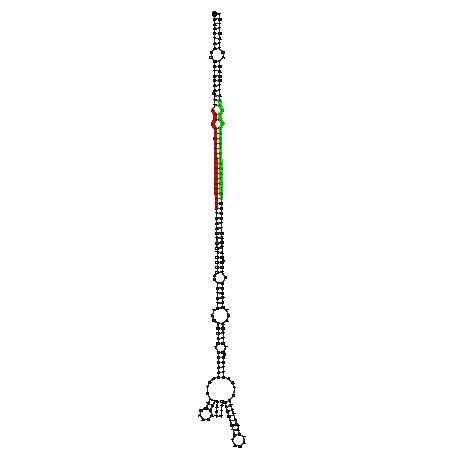

Supplement: Additional file 4 — Predicted secondary structures of pre-miRNAs of potato-specific miRNAs. Secondary structures of precursors of potato-specific miRNAs were predicted using RNAfold. The mature sequence is highlighted with green colour while star sequence is highlighted with red colour. 5′end is marked by a circle. [file 1471-2229-14-6-S4.zip › Additional file 4/miRNA 160.jpeg]

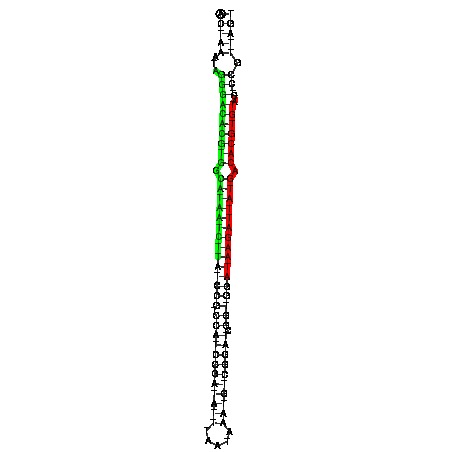

Supplement: Additional file 4 — Predicted secondary structures of pre-miRNAs of potato-specific miRNAs. Secondary structures of precursors of potato-specific miRNAs were predicted using RNAfold. The mature sequence is highlighted with green colour while star sequence is highlighted with red colour. 5′end is marked by a circle. [file 1471-2229-14-6-S4.zip › Additional file 4/miRNA 161.jpeg]

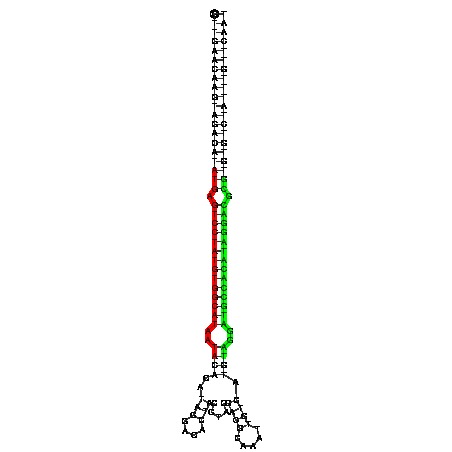

Supplement: Additional file 4 — Predicted secondary structures of pre-miRNAs of potato-specific miRNAs. Secondary structures of precursors of potato-specific miRNAs were predicted using RNAfold. The mature sequence is highlighted with green colour while star sequence is highlighted with red colour. 5′end is marked by a circle. [file 1471-2229-14-6-S4.zip › Additional file 4/miRNA 163.jpeg]

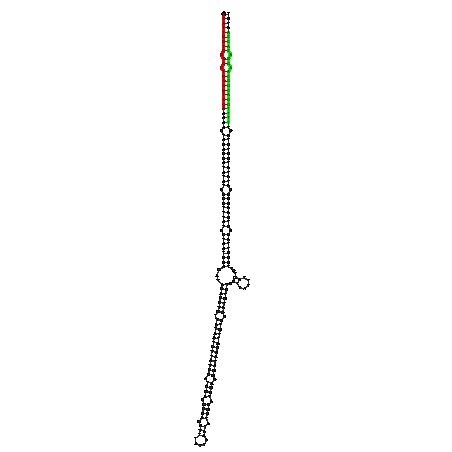

Supplement: Additional file 4 — Predicted secondary structures of pre-miRNAs of potato-specific miRNAs. Secondary structures of precursors of potato-specific miRNAs were predicted using RNAfold. The mature sequence is highlighted with green colour while star sequence is highlighted with red colour. 5′end is marked by a circle. [file 1471-2229-14-6-S4.zip › Additional file 4/miRNA 164.jpeg]

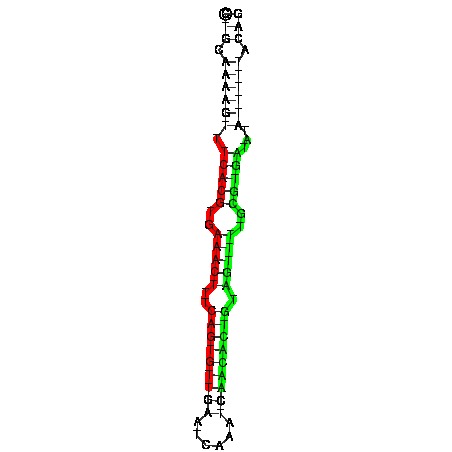

Supplement: Additional file 4 — Predicted secondary structures of pre-miRNAs of potato-specific miRNAs. Secondary structures of precursors of potato-specific miRNAs were predicted using RNAfold. The mature sequence is highlighted with green colour while star sequence is highlighted with red colour. 5′end is marked by a circle. [file 1471-2229-14-6-S4.zip › Additional file 4/miRNA 165.jpeg]

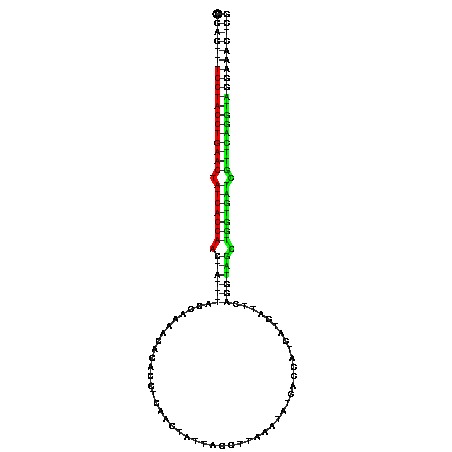

Supplement: Additional file 4 — Predicted secondary structures of pre-miRNAs of potato-specific miRNAs. Secondary structures of precursors of potato-specific miRNAs were predicted using RNAfold. The mature sequence is highlighted with green colour while star sequence is highlighted with red colour. 5′end is marked by a circle. [file 1471-2229-14-6-S4.zip › Additional file 4/miRNA 166.jpeg]

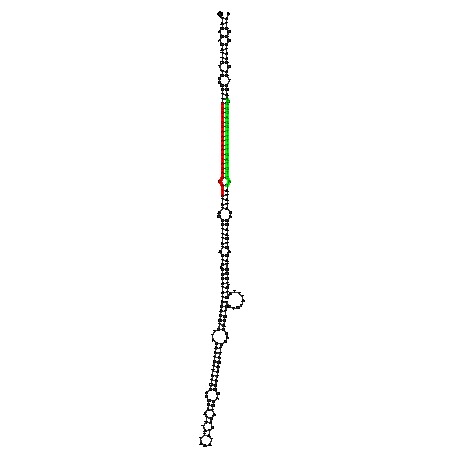

Supplement: Additional file 4 — Predicted secondary structures of pre-miRNAs of potato-specific miRNAs. Secondary structures of precursors of potato-specific miRNAs were predicted using RNAfold. The mature sequence is highlighted with green colour while star sequence is highlighted with red colour. 5′end is marked by a circle. [file 1471-2229-14-6-S4.zip › Additional file 4/miRNA 17.jpeg]

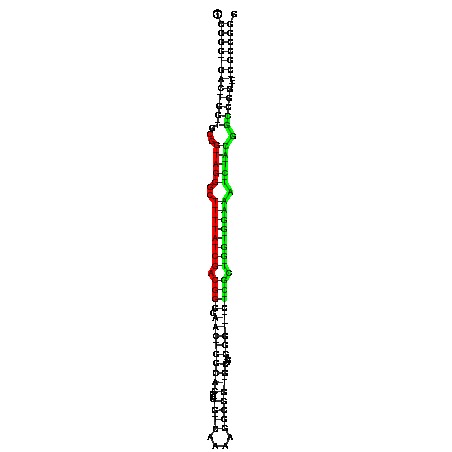

Supplement: Additional file 4 — Predicted secondary structures of pre-miRNAs of potato-specific miRNAs. Secondary structures of precursors of potato-specific miRNAs were predicted using RNAfold. The mature sequence is highlighted with green colour while star sequence is highlighted with red colour. 5′end is marked by a circle. [file 1471-2229-14-6-S4.zip › Additional file 4/miRNA 170.jpeg]

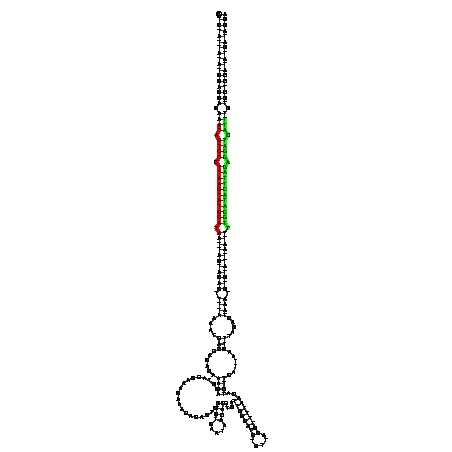

Supplement: Additional file 4 — Predicted secondary structures of pre-miRNAs of potato-specific miRNAs. Secondary structures of precursors of potato-specific miRNAs were predicted using RNAfold. The mature sequence is highlighted with green colour while star sequence is highlighted with red colour. 5′end is marked by a circle. [file 1471-2229-14-6-S4.zip › Additional file 4/miRNA 172.jpeg]

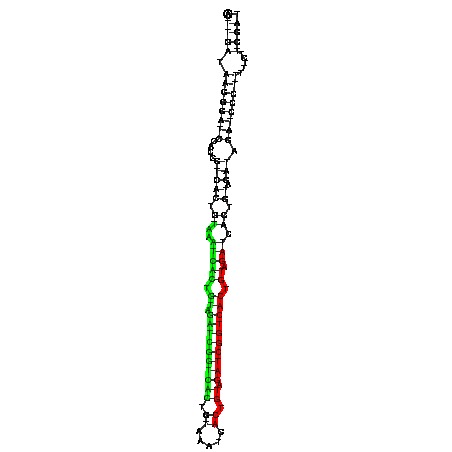

Supplement: Additional file 4 — Predicted secondary structures of pre-miRNAs of potato-specific miRNAs. Secondary structures of precursors of potato-specific miRNAs were predicted using RNAfold. The mature sequence is highlighted with green colour while star sequence is highlighted with red colour. 5′end is marked by a circle. [file 1471-2229-14-6-S4.zip › Additional file 4/miRNA 174.jpeg]

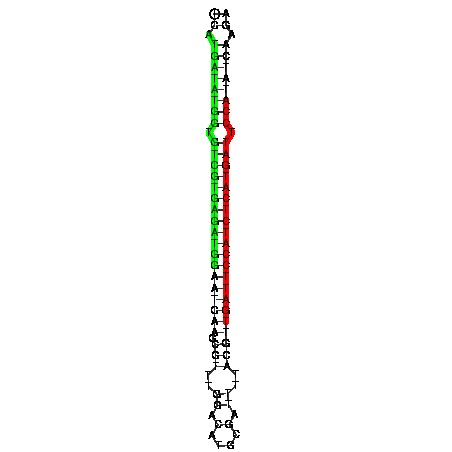

Supplement: Additional file 4 — Predicted secondary structures of pre-miRNAs of potato-specific miRNAs. Secondary structures of precursors of potato-specific miRNAs were predicted using RNAfold. The mature sequence is highlighted with green colour while star sequence is highlighted with red colour. 5′end is marked by a circle. [file 1471-2229-14-6-S4.zip › Additional file 4/miRNA 177.jpeg]

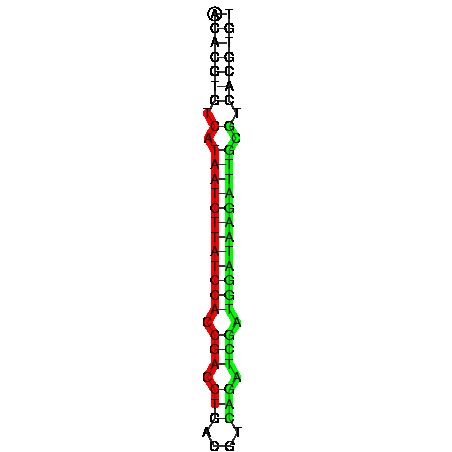

Supplement: Additional file 4 — Predicted secondary structures of pre-miRNAs of potato-specific miRNAs. Secondary structures of precursors of potato-specific miRNAs were predicted using RNAfold. The mature sequence is highlighted with green colour while star sequence is highlighted with red colour. 5′end is marked by a circle. [file 1471-2229-14-6-S4.zip › Additional file 4/miRNA 178.jpeg]

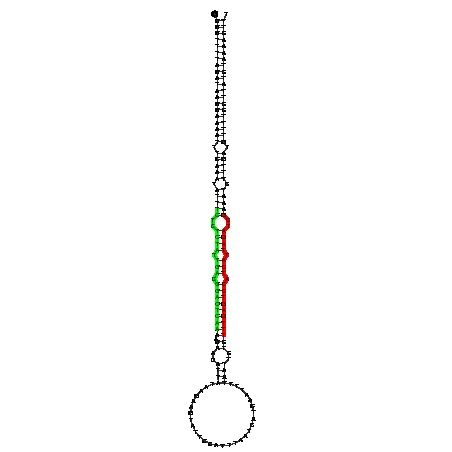

Supplement: Additional file 4 — Predicted secondary structures of pre-miRNAs of potato-specific miRNAs. Secondary structures of precursors of potato-specific miRNAs were predicted using RNAfold. The mature sequence is highlighted with green colour while star sequence is highlighted with red colour. 5′end is marked by a circle. [file 1471-2229-14-6-S4.zip › Additional file 4/miRNA 179.jpeg]

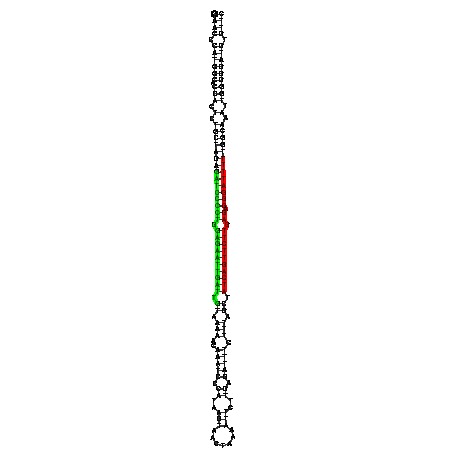

Supplement: Additional file 4 — Predicted secondary structures of pre-miRNAs of potato-specific miRNAs. Secondary structures of precursors of potato-specific miRNAs were predicted using RNAfold. The mature sequence is highlighted with green colour while star sequence is highlighted with red colour. 5′end is marked by a circle. [file 1471-2229-14-6-S4.zip › Additional file 4/miRNA 18.jpeg]

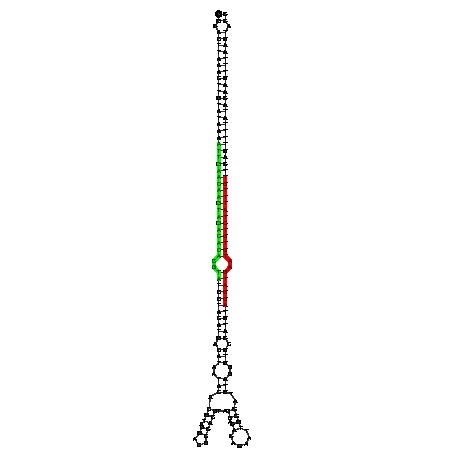

Supplement: Additional file 4 — Predicted secondary structures of pre-miRNAs of potato-specific miRNAs. Secondary structures of precursors of potato-specific miRNAs were predicted using RNAfold. The mature sequence is highlighted with green colour while star sequence is highlighted with red colour. 5′end is marked by a circle. [file 1471-2229-14-6-S4.zip › Additional file 4/miRNA 181.jpeg]

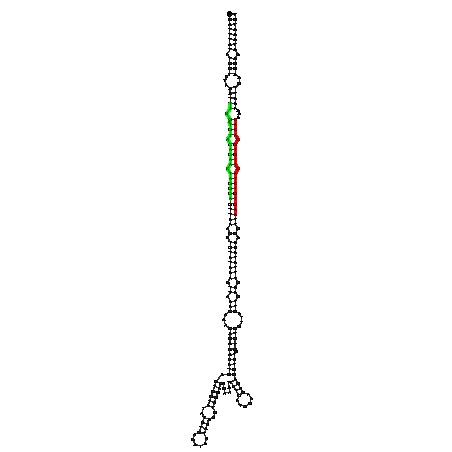

Supplement: Additional file 4 — Predicted secondary structures of pre-miRNAs of potato-specific miRNAs. Secondary structures of precursors of potato-specific miRNAs were predicted using RNAfold. The mature sequence is highlighted with green colour while star sequence is highlighted with red colour. 5′end is marked by a circle. [file 1471-2229-14-6-S4.zip › Additional file 4/miRNA 182.jpeg]

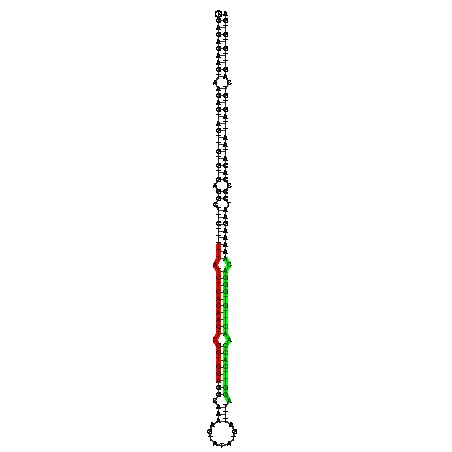

Supplement: Additional file 4 — Predicted secondary structures of pre-miRNAs of potato-specific miRNAs. Secondary structures of precursors of potato-specific miRNAs were predicted using RNAfold. The mature sequence is highlighted with green colour while star sequence is highlighted with red colour. 5′end is marked by a circle. [file 1471-2229-14-6-S4.zip › Additional file 4/miRNA 185.jpeg]

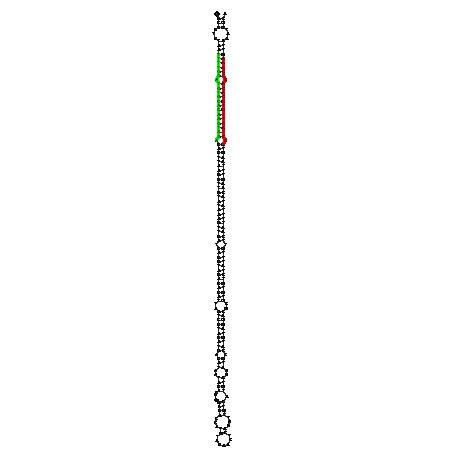

Supplement: Additional file 4 — Predicted secondary structures of pre-miRNAs of potato-specific miRNAs. Secondary structures of precursors of potato-specific miRNAs were predicted using RNAfold. The mature sequence is highlighted with green colour while star sequence is highlighted with red colour. 5′end is marked by a circle. [file 1471-2229-14-6-S4.zip › Additional file 4/miRNA 186.jpeg]

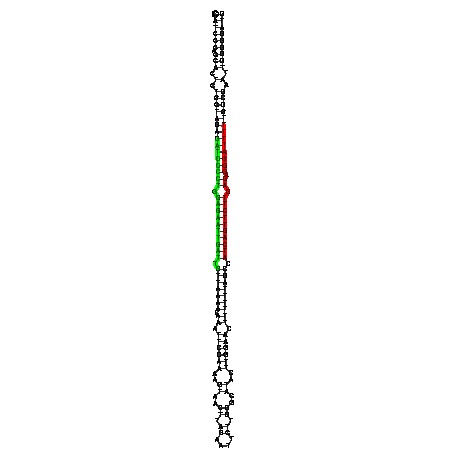

Supplement: Additional file 4 — Predicted secondary structures of pre-miRNAs of potato-specific miRNAs. Secondary structures of precursors of potato-specific miRNAs were predicted using RNAfold. The mature sequence is highlighted with green colour while star sequence is highlighted with red colour. 5′end is marked by a circle. [file 1471-2229-14-6-S4.zip › Additional file 4/miRNA 19.jpeg]

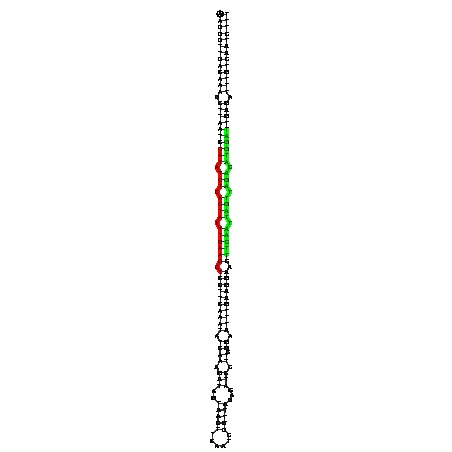

Supplement: Additional file 4 — Predicted secondary structures of pre-miRNAs of potato-specific miRNAs. Secondary structures of precursors of potato-specific miRNAs were predicted using RNAfold. The mature sequence is highlighted with green colour while star sequence is highlighted with red colour. 5′end is marked by a circle. [file 1471-2229-14-6-S4.zip › Additional file 4/miRNA 191.jpeg]

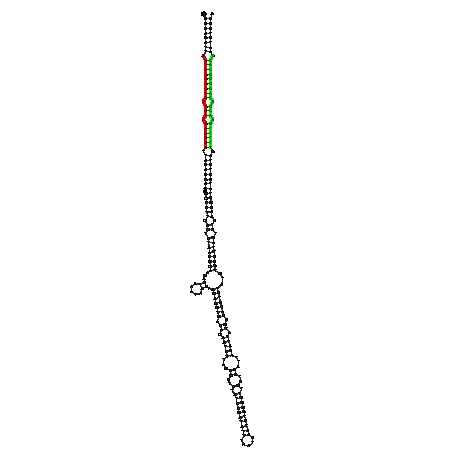

Supplement: Additional file 4 — Predicted secondary structures of pre-miRNAs of potato-specific miRNAs. Secondary structures of precursors of potato-specific miRNAs were predicted using RNAfold. The mature sequence is highlighted with green colour while star sequence is highlighted with red colour. 5′end is marked by a circle. [file 1471-2229-14-6-S4.zip › Additional file 4/miRNA 192.jpeg]

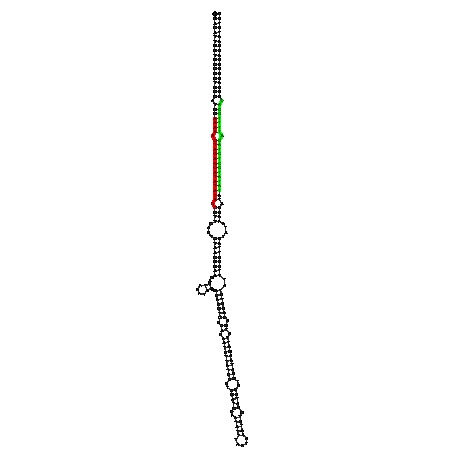

Supplement: Additional file 4 — Predicted secondary structures of pre-miRNAs of potato-specific miRNAs. Secondary structures of precursors of potato-specific miRNAs were predicted using RNAfold. The mature sequence is highlighted with green colour while star sequence is highlighted with red colour. 5′end is marked by a circle. [file 1471-2229-14-6-S4.zip › Additional file 4/miRNA 193.jpeg]

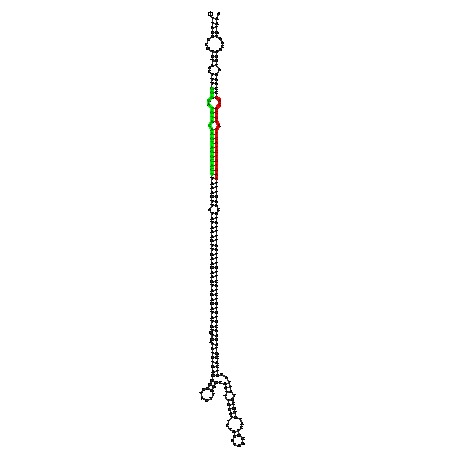

Supplement: Additional file 4 — Predicted secondary structures of pre-miRNAs of potato-specific miRNAs. Secondary structures of precursors of potato-specific miRNAs were predicted using RNAfold. The mature sequence is highlighted with green colour while star sequence is highlighted with red colour. 5′end is marked by a circle. [file 1471-2229-14-6-S4.zip › Additional file 4/miRNA 195.jpeg]

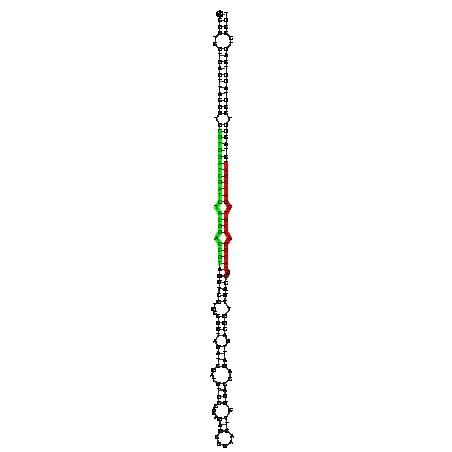

Supplement: Additional file 4 — Predicted secondary structures of pre-miRNAs of potato-specific miRNAs. Secondary structures of precursors of potato-specific miRNAs were predicted using RNAfold. The mature sequence is highlighted with green colour while star sequence is highlighted with red colour. 5′end is marked by a circle. [file 1471-2229-14-6-S4.zip › Additional file 4/miRNA 196.jpeg]

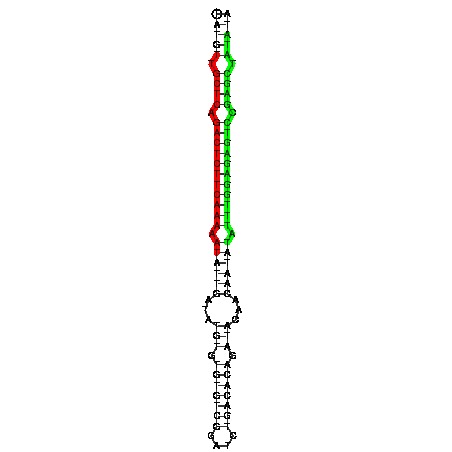

Supplement: Additional file 4 — Predicted secondary structures of pre-miRNAs of potato-specific miRNAs. Secondary structures of precursors of potato-specific miRNAs were predicted using RNAfold. The mature sequence is highlighted with green colour while star sequence is highlighted with red colour. 5′end is marked by a circle. [file 1471-2229-14-6-S4.zip › Additional file 4/miRNA 198.jpeg]

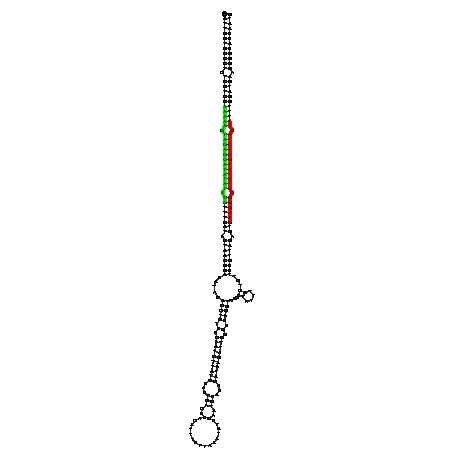

Supplement: Additional file 4 — Predicted secondary structures of pre-miRNAs of potato-specific miRNAs. Secondary structures of precursors of potato-specific miRNAs were predicted using RNAfold. The mature sequence is highlighted with green colour while star sequence is highlighted with red colour. 5′end is marked by a circle. [file 1471-2229-14-6-S4.zip › Additional file 4/miRNA 199.jpeg]

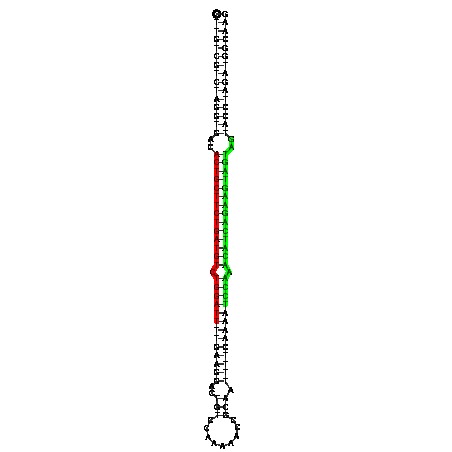

Supplement: Additional file 4 — Predicted secondary structures of pre-miRNAs of potato-specific miRNAs. Secondary structures of precursors of potato-specific miRNAs were predicted using RNAfold. The mature sequence is highlighted with green colour while star sequence is highlighted with red colour. 5′end is marked by a circle. [file 1471-2229-14-6-S4.zip › Additional file 4/miRNA 2.jpeg]

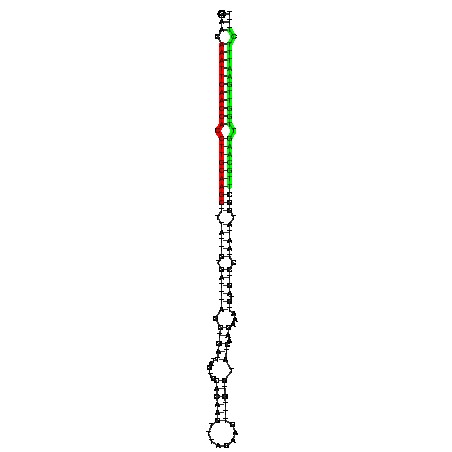

Supplement: Additional file 4 — Predicted secondary structures of pre-miRNAs of potato-specific miRNAs. Secondary structures of precursors of potato-specific miRNAs were predicted using RNAfold. The mature sequence is highlighted with green colour while star sequence is highlighted with red colour. 5′end is marked by a circle. [file 1471-2229-14-6-S4.zip › Additional file 4/miRNA 20.jpeg]

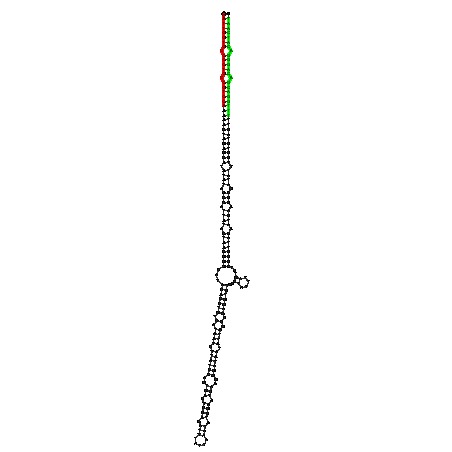

Supplement: Additional file 4 — Predicted secondary structures of pre-miRNAs of potato-specific miRNAs. Secondary structures of precursors of potato-specific miRNAs were predicted using RNAfold. The mature sequence is highlighted with green colour while star sequence is highlighted with red colour. 5′end is marked by a circle. [file 1471-2229-14-6-S4.zip › Additional file 4/miRNA 200.jpeg]

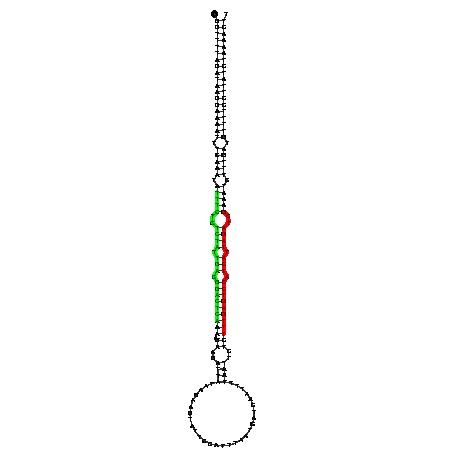

Supplement: Additional file 4 — Predicted secondary structures of pre-miRNAs of potato-specific miRNAs. Secondary structures of precursors of potato-specific miRNAs were predicted using RNAfold. The mature sequence is highlighted with green colour while star sequence is highlighted with red colour. 5′end is marked by a circle. [file 1471-2229-14-6-S4.zip › Additional file 4/miRNA 201.jpeg]

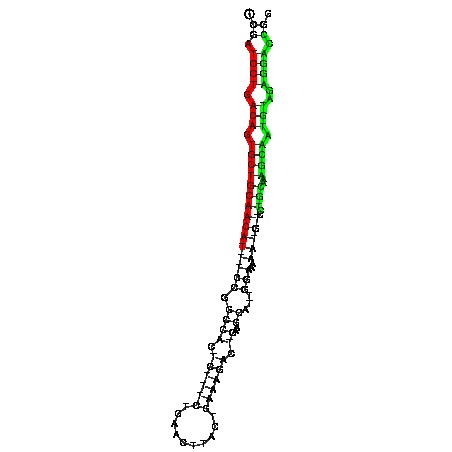

Supplement: Additional file 4 — Predicted secondary structures of pre-miRNAs of potato-specific miRNAs. Secondary structures of precursors of potato-specific miRNAs were predicted using RNAfold. The mature sequence is highlighted with green colour while star sequence is highlighted with red colour. 5′end is marked by a circle. [file 1471-2229-14-6-S4.zip › Additional file 4/miRNA 203.jpeg]

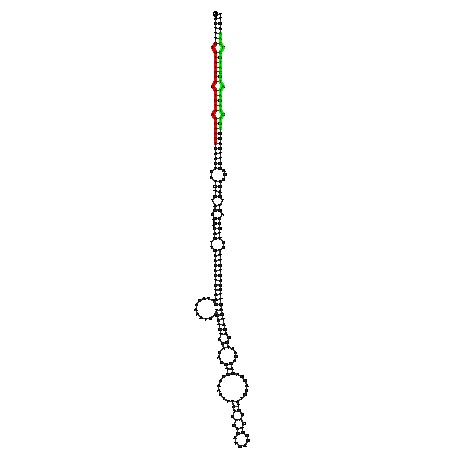

Supplement: Additional file 4 — Predicted secondary structures of pre-miRNAs of potato-specific miRNAs. Secondary structures of precursors of potato-specific miRNAs were predicted using RNAfold. The mature sequence is highlighted with green colour while star sequence is highlighted with red colour. 5′end is marked by a circle. [file 1471-2229-14-6-S4.zip › Additional file 4/miRNA 204.jpeg]

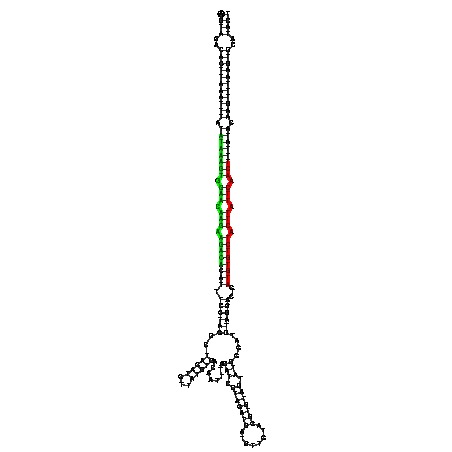

Supplement: Additional file 4 — Predicted secondary structures of pre-miRNAs of potato-specific miRNAs. Secondary structures of precursors of potato-specific miRNAs were predicted using RNAfold. The mature sequence is highlighted with green colour while star sequence is highlighted with red colour. 5′end is marked by a circle. [file 1471-2229-14-6-S4.zip › Additional file 4/miRNA 205.jpeg]

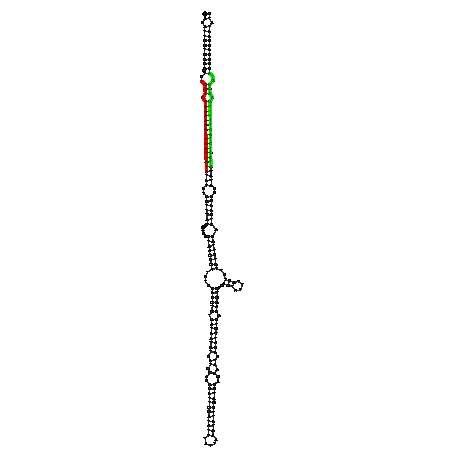

Supplement: Additional file 4 — Predicted secondary structures of pre-miRNAs of potato-specific miRNAs. Secondary structures of precursors of potato-specific miRNAs were predicted using RNAfold. The mature sequence is highlighted with green colour while star sequence is highlighted with red colour. 5′end is marked by a circle. [file 1471-2229-14-6-S4.zip › Additional file 4/miRNA 206.jpeg]

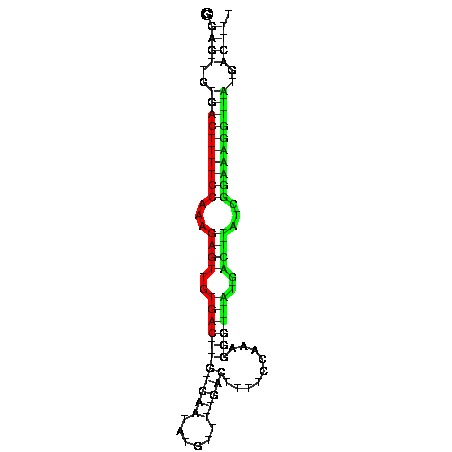

Supplement: Additional file 4 — Predicted secondary structures of pre-miRNAs of potato-specific miRNAs. Secondary structures of precursors of potato-specific miRNAs were predicted using RNAfold. The mature sequence is highlighted with green colour while star sequence is highlighted with red colour. 5′end is marked by a circle. [file 1471-2229-14-6-S4.zip › Additional file 4/miRNA 207.jpeg]

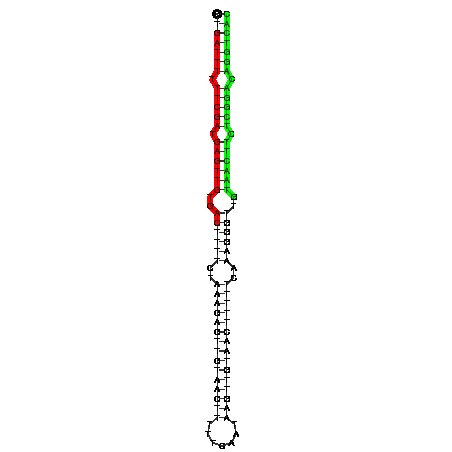

Supplement: Additional file 4 — Predicted secondary structures of pre-miRNAs of potato-specific miRNAs. Secondary structures of precursors of potato-specific miRNAs were predicted using RNAfold. The mature sequence is highlighted with green colour while star sequence is highlighted with red colour. 5′end is marked by a circle. [file 1471-2229-14-6-S4.zip › Additional file 4/miRNA 208.jpeg]

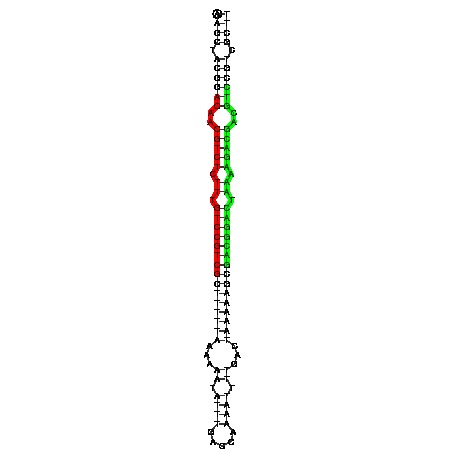

Supplement: Additional file 4 — Predicted secondary structures of pre-miRNAs of potato-specific miRNAs. Secondary structures of precursors of potato-specific miRNAs were predicted using RNAfold. The mature sequence is highlighted with green colour while star sequence is highlighted with red colour. 5′end is marked by a circle. [file 1471-2229-14-6-S4.zip › Additional file 4/miRNA 209.jpeg]

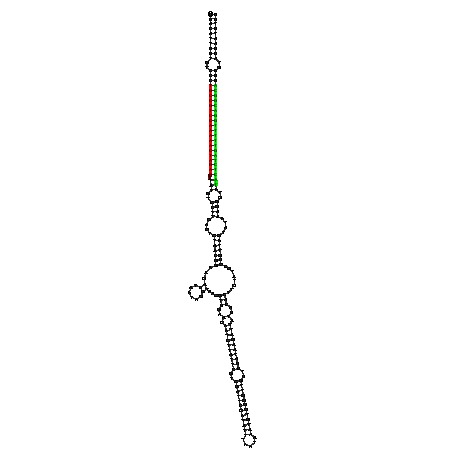

Supplement: Additional file 4 — Predicted secondary structures of pre-miRNAs of potato-specific miRNAs. Secondary structures of precursors of potato-specific miRNAs were predicted using RNAfold. The mature sequence is highlighted with green colour while star sequence is highlighted with red colour. 5′end is marked by a circle. [file 1471-2229-14-6-S4.zip › Additional file 4/miRNA 21.jpeg]

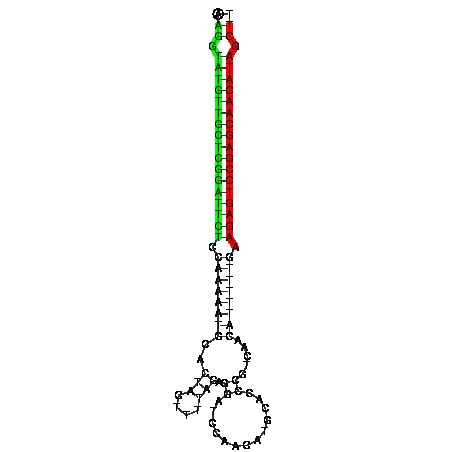

Supplement: Additional file 4 — Predicted secondary structures of pre-miRNAs of potato-specific miRNAs. Secondary structures of precursors of potato-specific miRNAs were predicted using RNAfold. The mature sequence is highlighted with green colour while star sequence is highlighted with red colour. 5′end is marked by a circle. [file 1471-2229-14-6-S4.zip › Additional file 4/miRNA 210.jpeg]

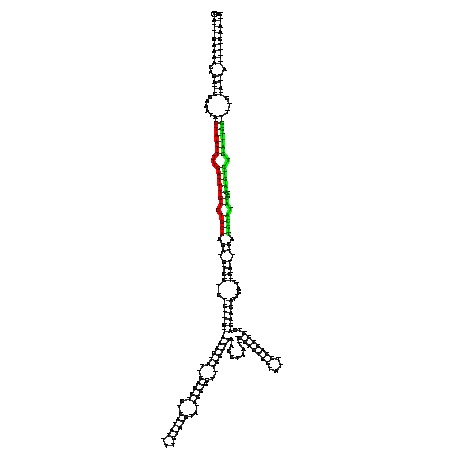

Supplement: Additional file 4 — Predicted secondary structures of pre-miRNAs of potato-specific miRNAs. Secondary structures of precursors of potato-specific miRNAs were predicted using RNAfold. The mature sequence is highlighted with green colour while star sequence is highlighted with red colour. 5′end is marked by a circle. [file 1471-2229-14-6-S4.zip › Additional file 4/miRNA 211.jpeg]

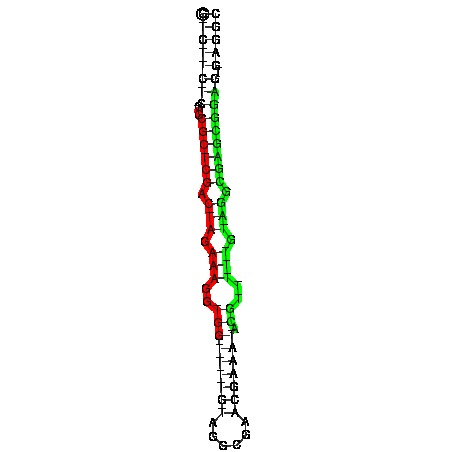

Supplement: Additional file 4 — Predicted secondary structures of pre-miRNAs of potato-specific miRNAs. Secondary structures of precursors of potato-specific miRNAs were predicted using RNAfold. The mature sequence is highlighted with green colour while star sequence is highlighted with red colour. 5′end is marked by a circle. [file 1471-2229-14-6-S4.zip › Additional file 4/miRNA 212.jpeg]

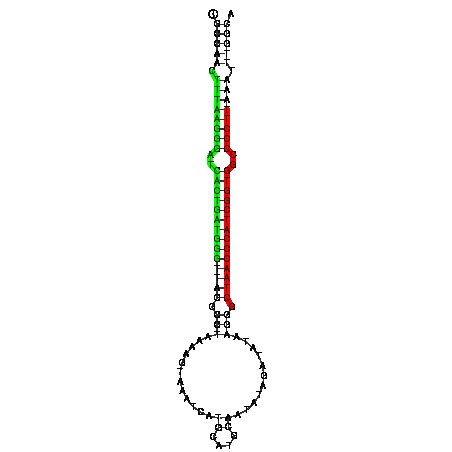

Supplement: Additional file 4 — Predicted secondary structures of pre-miRNAs of potato-specific miRNAs. Secondary structures of precursors of potato-specific miRNAs were predicted using RNAfold. The mature sequence is highlighted with green colour while star sequence is highlighted with red colour. 5′end is marked by a circle. [file 1471-2229-14-6-S4.zip › Additional file 4/miRNA 213.jpeg]

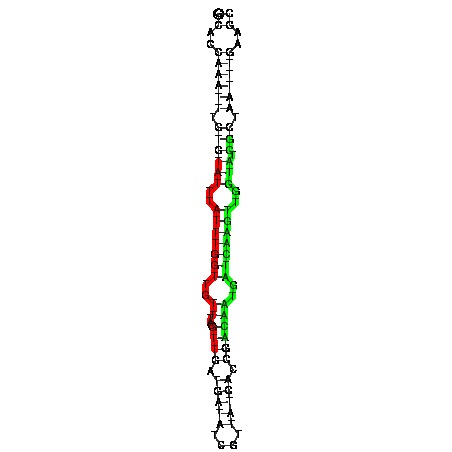

Supplement: Additional file 4 — Predicted secondary structures of pre-miRNAs of potato-specific miRNAs. Secondary structures of precursors of potato-specific miRNAs were predicted using RNAfold. The mature sequence is highlighted with green colour while star sequence is highlighted with red colour. 5′end is marked by a circle. [file 1471-2229-14-6-S4.zip › Additional file 4/miRNA 214.jpeg]

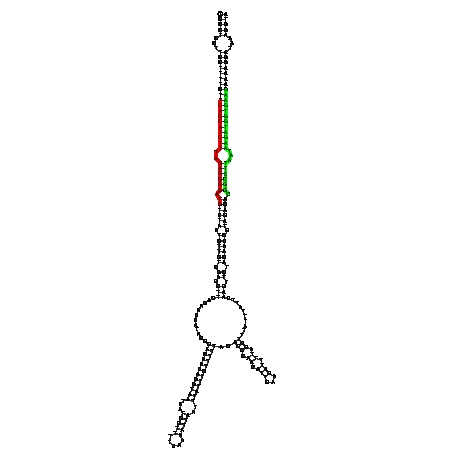

Supplement: Additional file 4 — Predicted secondary structures of pre-miRNAs of potato-specific miRNAs. Secondary structures of precursors of potato-specific miRNAs were predicted using RNAfold. The mature sequence is highlighted with green colour while star sequence is highlighted with red colour. 5′end is marked by a circle. [file 1471-2229-14-6-S4.zip › Additional file 4/miRNA 216.jpeg]

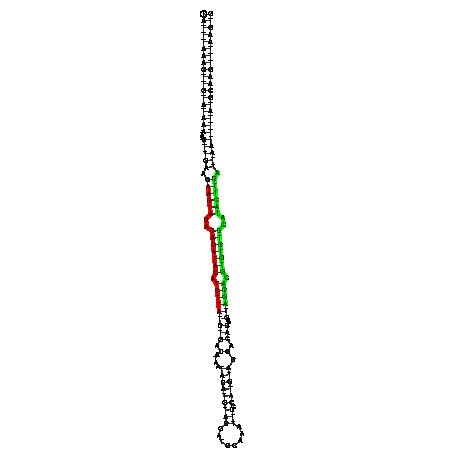

Supplement: Additional file 4 — Predicted secondary structures of pre-miRNAs of potato-specific miRNAs. Secondary structures of precursors of potato-specific miRNAs were predicted using RNAfold. The mature sequence is highlighted with green colour while star sequence is highlighted with red colour. 5′end is marked by a circle. [file 1471-2229-14-6-S4.zip › Additional file 4/miRNA 217.jpeg]

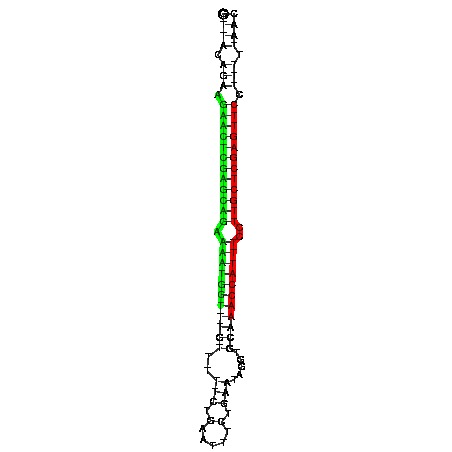

Supplement: Additional file 4 — Predicted secondary structures of pre-miRNAs of potato-specific miRNAs. Secondary structures of precursors of potato-specific miRNAs were predicted using RNAfold. The mature sequence is highlighted with green colour while star sequence is highlighted with red colour. 5′end is marked by a circle. [file 1471-2229-14-6-S4.zip › Additional file 4/miRNA 22.jpeg]

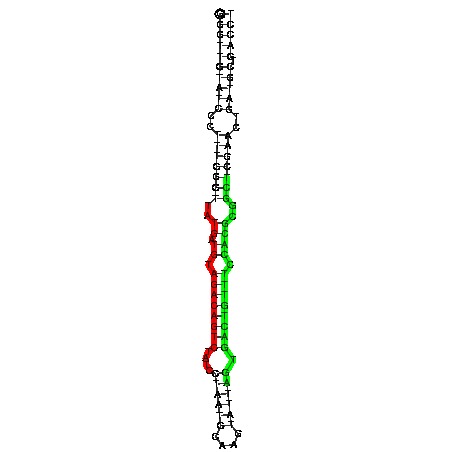

Supplement: Additional file 4 — Predicted secondary structures of pre-miRNAs of potato-specific miRNAs. Secondary structures of precursors of potato-specific miRNAs were predicted using RNAfold. The mature sequence is highlighted with green colour while star sequence is highlighted with red colour. 5′end is marked by a circle. [file 1471-2229-14-6-S4.zip › Additional file 4/miRNA 220.jpeg]

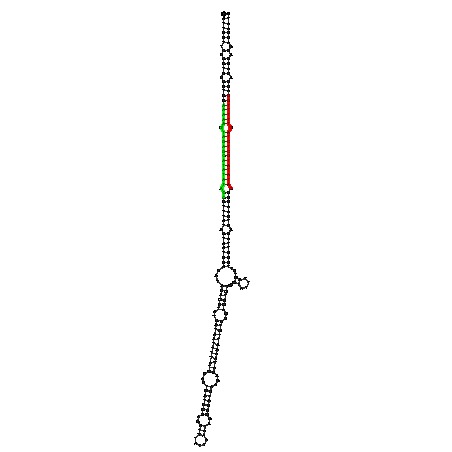

Supplement: Additional file 4 — Predicted secondary structures of pre-miRNAs of potato-specific miRNAs. Secondary structures of precursors of potato-specific miRNAs were predicted using RNAfold. The mature sequence is highlighted with green colour while star sequence is highlighted with red colour. 5′end is marked by a circle. [file 1471-2229-14-6-S4.zip › Additional file 4/miRNA 223.jpeg]

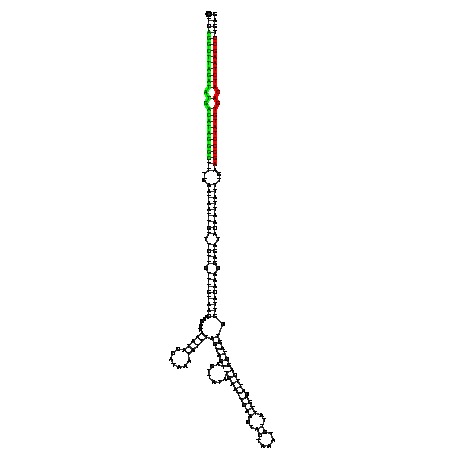

Supplement: Additional file 4 — Predicted secondary structures of pre-miRNAs of potato-specific miRNAs. Secondary structures of precursors of potato-specific miRNAs were predicted using RNAfold. The mature sequence is highlighted with green colour while star sequence is highlighted with red colour. 5′end is marked by a circle. [file 1471-2229-14-6-S4.zip › Additional file 4/miRNA 224.jpeg]

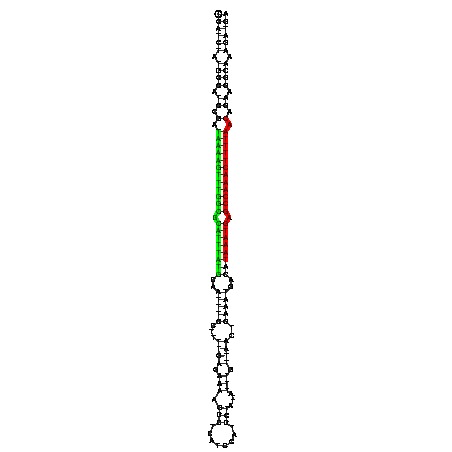

Supplement: Additional file 4 — Predicted secondary structures of pre-miRNAs of potato-specific miRNAs. Secondary structures of precursors of potato-specific miRNAs were predicted using RNAfold. The mature sequence is highlighted with green colour while star sequence is highlighted with red colour. 5′end is marked by a circle. [file 1471-2229-14-6-S4.zip › Additional file 4/miRNA 23.jpeg]

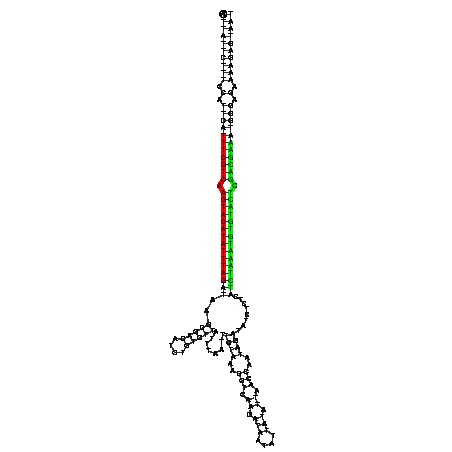

Supplement: Additional file 4 — Predicted secondary structures of pre-miRNAs of potato-specific miRNAs. Secondary structures of precursors of potato-specific miRNAs were predicted using RNAfold. The mature sequence is highlighted with green colour while star sequence is highlighted with red colour. 5′end is marked by a circle. [file 1471-2229-14-6-S4.zip › Additional file 4/miRNA 26.jpeg]

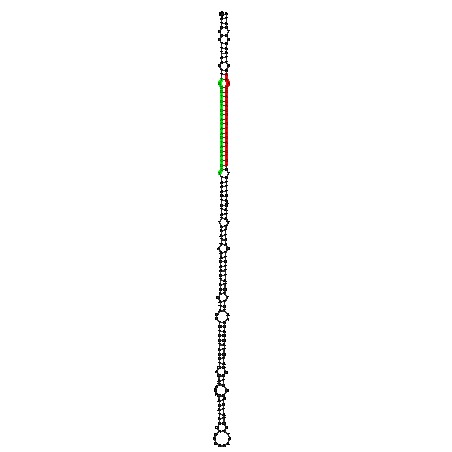

Supplement: Additional file 4 — Predicted secondary structures of pre-miRNAs of potato-specific miRNAs. Secondary structures of precursors of potato-specific miRNAs were predicted using RNAfold. The mature sequence is highlighted with green colour while star sequence is highlighted with red colour. 5′end is marked by a circle. [file 1471-2229-14-6-S4.zip › Additional file 4/miRNA 27.jpeg]

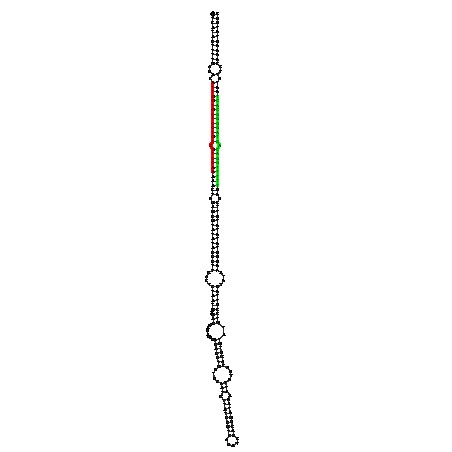

Supplement: Additional file 4 — Predicted secondary structures of pre-miRNAs of potato-specific miRNAs. Secondary structures of precursors of potato-specific miRNAs were predicted using RNAfold. The mature sequence is highlighted with green colour while star sequence is highlighted with red colour. 5′end is marked by a circle. [file 1471-2229-14-6-S4.zip › Additional file 4/miRNA 28.jpeg]

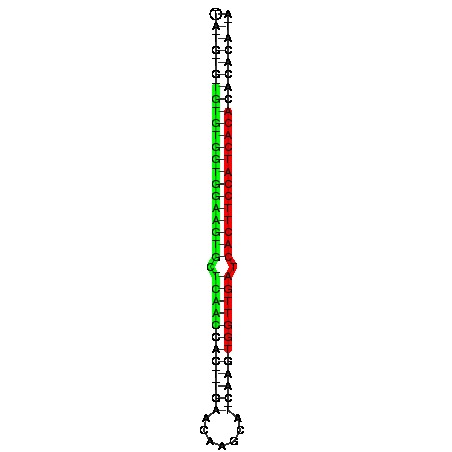

Supplement: Additional file 4 — Predicted secondary structures of pre-miRNAs of potato-specific miRNAs. Secondary structures of precursors of potato-specific miRNAs were predicted using RNAfold. The mature sequence is highlighted with green colour while star sequence is highlighted with red colour. 5′end is marked by a circle. [file 1471-2229-14-6-S4.zip › Additional file 4/miRNA 29.jpeg]

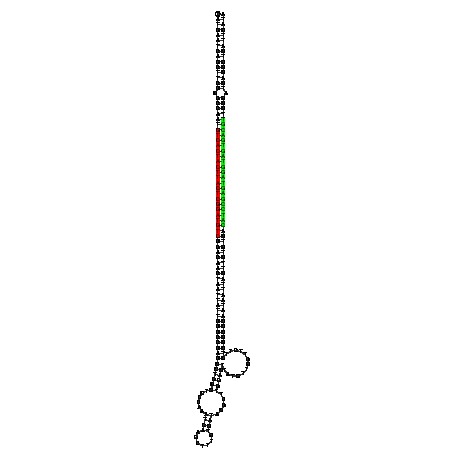

Supplement: Additional file 4 — Predicted secondary structures of pre-miRNAs of potato-specific miRNAs. Secondary structures of precursors of potato-specific miRNAs were predicted using RNAfold. The mature sequence is highlighted with green colour while star sequence is highlighted with red colour. 5′end is marked by a circle. [file 1471-2229-14-6-S4.zip › Additional file 4/miRNA 30.jpeg]

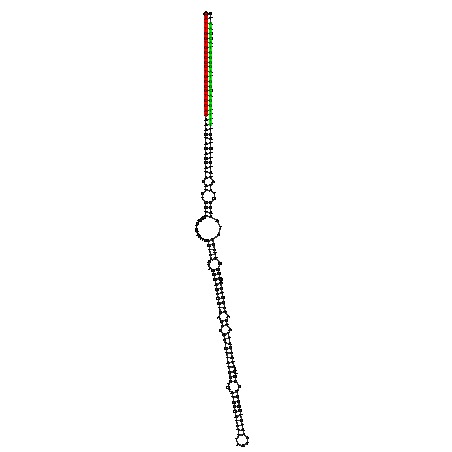

Supplement: Additional file 4 — Predicted secondary structures of pre-miRNAs of potato-specific miRNAs. Secondary structures of precursors of potato-specific miRNAs were predicted using RNAfold. The mature sequence is highlighted with green colour while star sequence is highlighted with red colour. 5′end is marked by a circle. [file 1471-2229-14-6-S4.zip › Additional file 4/miRNA 31.jpeg]

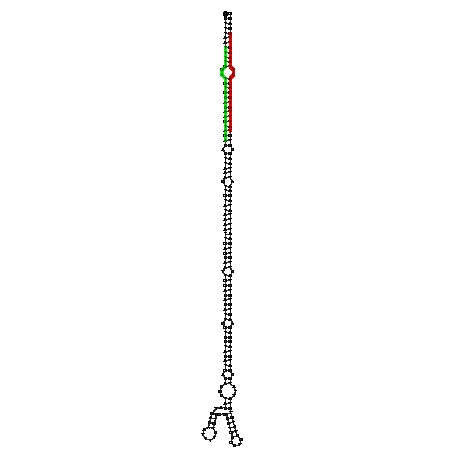

Supplement: Additional file 4 — Predicted secondary structures of pre-miRNAs of potato-specific miRNAs. Secondary structures of precursors of potato-specific miRNAs were predicted using RNAfold. The mature sequence is highlighted with green colour while star sequence is highlighted with red colour. 5′end is marked by a circle. [file 1471-2229-14-6-S4.zip › Additional file 4/miRNA 32.jpeg]

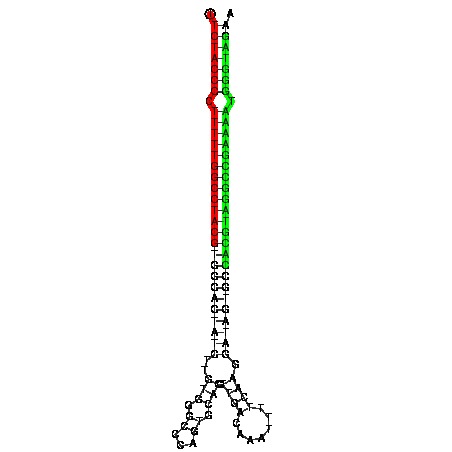

Supplement: Additional file 4 — Predicted secondary structures of pre-miRNAs of potato-specific miRNAs. Secondary structures of precursors of potato-specific miRNAs were predicted using RNAfold. The mature sequence is highlighted with green colour while star sequence is highlighted with red colour. 5′end is marked by a circle. [file 1471-2229-14-6-S4.zip › Additional file 4/miRNA 33.jpeg]

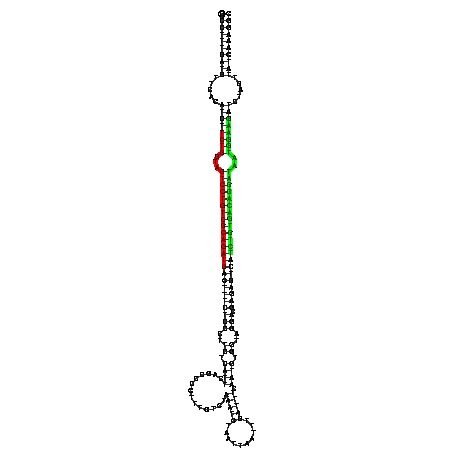

Supplement: Additional file 4 — Predicted secondary structures of pre-miRNAs of potato-specific miRNAs. Secondary structures of precursors of potato-specific miRNAs were predicted using RNAfold. The mature sequence is highlighted with green colour while star sequence is highlighted with red colour. 5′end is marked by a circle. [file 1471-2229-14-6-S4.zip › Additional file 4/miRNA 34.jpeg]

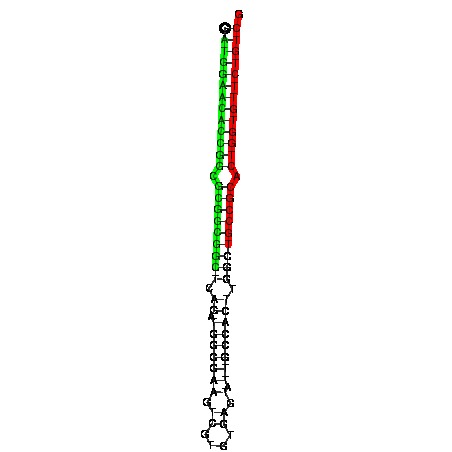

Supplement: Additional file 4 — Predicted secondary structures of pre-miRNAs of potato-specific miRNAs. Secondary structures of precursors of potato-specific miRNAs were predicted using RNAfold. The mature sequence is highlighted with green colour while star sequence is highlighted with red colour. 5′end is marked by a circle. [file 1471-2229-14-6-S4.zip › Additional file 4/miRNA 35.jpeg]

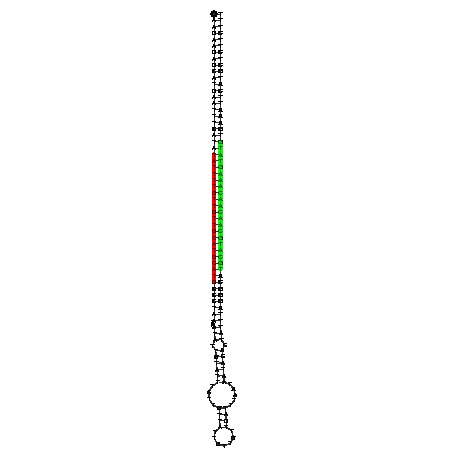

Supplement: Additional file 4 — Predicted secondary structures of pre-miRNAs of potato-specific miRNAs. Secondary structures of precursors of potato-specific miRNAs were predicted using RNAfold. The mature sequence is highlighted with green colour while star sequence is highlighted with red colour. 5′end is marked by a circle. [file 1471-2229-14-6-S4.zip › Additional file 4/miRNA 36.jpeg]

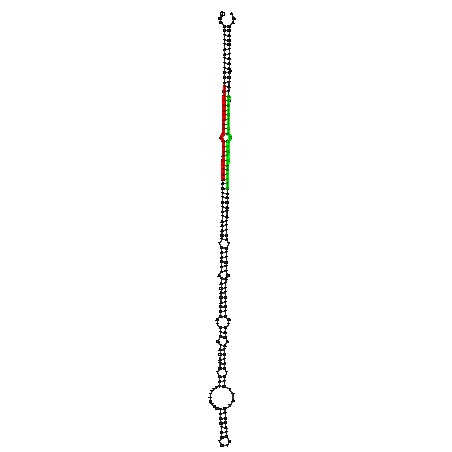

Supplement: Additional file 4 — Predicted secondary structures of pre-miRNAs of potato-specific miRNAs. Secondary structures of precursors of potato-specific miRNAs were predicted using RNAfold. The mature sequence is highlighted with green colour while star sequence is highlighted with red colour. 5′end is marked by a circle. [file 1471-2229-14-6-S4.zip › Additional file 4/miRNA 37.jpeg]

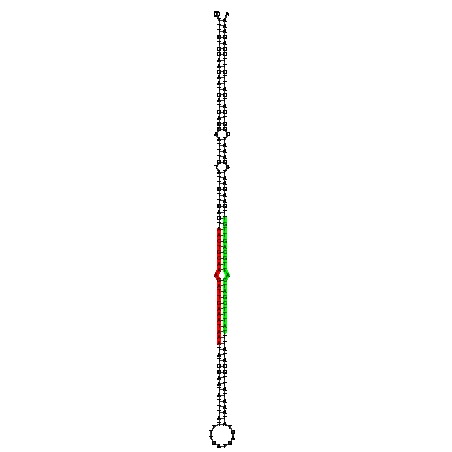

Supplement: Additional file 4 — Predicted secondary structures of pre-miRNAs of potato-specific miRNAs. Secondary structures of precursors of potato-specific miRNAs were predicted using RNAfold. The mature sequence is highlighted with green colour while star sequence is highlighted with red colour. 5′end is marked by a circle. [file 1471-2229-14-6-S4.zip › Additional file 4/miRNA 38.jpeg]

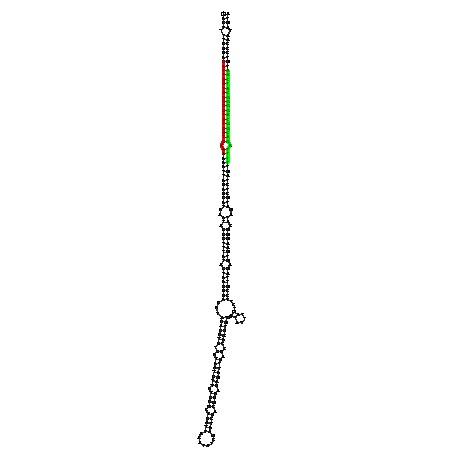

Supplement: Additional file 4 — Predicted secondary structures of pre-miRNAs of potato-specific miRNAs. Secondary structures of precursors of potato-specific miRNAs were predicted using RNAfold. The mature sequence is highlighted with green colour while star sequence is highlighted with red colour. 5′end is marked by a circle. [file 1471-2229-14-6-S4.zip › Additional file 4/miRNA 39.jpeg]

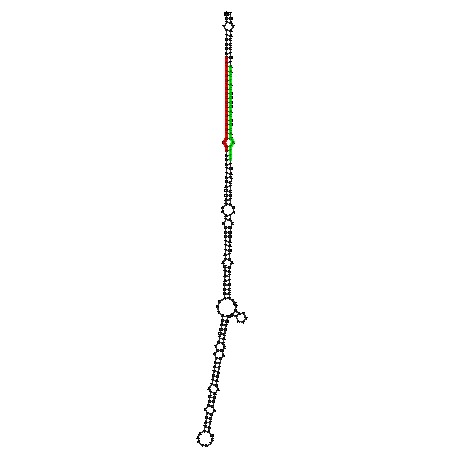

Supplement: Additional file 4 — Predicted secondary structures of pre-miRNAs of potato-specific miRNAs. Secondary structures of precursors of potato-specific miRNAs were predicted using RNAfold. The mature sequence is highlighted with green colour while star sequence is highlighted with red colour. 5′end is marked by a circle. [file 1471-2229-14-6-S4.zip › Additional file 4/miRNA 40.jpeg]
